# Complete blood count reference intervals from a healthy adult urban population in Kenya

**DOI:** 10.1371/journal.pone.0198444

**Published:** 2018-06-07

**Authors:** Geoffrey Omuse, Daniel Maina, Jane Mwangi, Caroline Wambua, Kiran Radia, Alice Kanyua, Elizabeth Kagotho, Mariza Hoffman, Peter Ojwang, Zul Premji, Kiyoshi Ichihara, Rajiv Erasmus

**Affiliations:** 1 Department of Pathology, Aga Khan University Hospital Nairobi, Nairobi, Kenya; 2 Division of Chemical Pathology, Department of Pathology, Stellenbosch University, Cape Town, South Africa; 3 PathCare Kenya Limited, Nairobi, Kenya; 4 Karen Hospital, Nairobi, Kenya; 5 Department of Pathology, Maseno University, Maseno, Kenya; 6 Formerly of Muhimbili University of Health and Allied Sciences, Dar es Salaam, Tanzania; 7 Faculty of Health Sciences, Yamaguchi University Graduate School of Medicine, Ube, Japan; Inselspital Universitatsspital Bern, SWITZERLAND

## Abstract

**Background:**

There are racial, ethnic and geographical differences in complete blood count (CBC) reference intervals (RIs) and therefore it is necessary to establish RIs that are population specific. Several studies have been carried out in Africa to derive CBC RIs but many were not conducted with the rigor recommended for RI studies hence limiting the adoption and generalizability of the results.

**Method:**

By use of a Beckman Coulter ACT 5 DIFF CP analyser, we measured CBC parameters in samples collected from 528 healthy black African volunteers in a largely urban population. The latent abnormal values exclusion (LAVE) method was used for secondary exclusion of individuals who may have had sub-clinical diseases. The RIs were derived by both parametric and non-parametric methods with and without LAVE for comparative purposes.

**Results:**

Haemoglobin (Hb) levels were lower while platelet counts were higher in females across the 4 age stratifications. The lower limits for Hb and red blood cell parameters significantly increased after applying the LAVE method which eliminated individuals with latent anemia and inflammation. We adopted RIs by parametric method because 90% confidence intervals of the RI limits were invariably narrower than those by the non-parametric method. The male and female RIs for Hb after applying the LAVE method were 14.5–18.7 g/dL and 12.0–16.5 g/dL respectively while the platelet count RIs were 133–356 and 152–443 x10^3^ per μL respectively.

**Conclusion:**

Consistent with other studies from Sub-Saharan Africa, Hb and neutrophil counts were lower than Caucasian values. Our finding of higher Hb and lower eosinophil counts compared to other studies conducted in rural Kenya most likely reflects the strict recruitment criteria and healthier reference population after secondary exclusion of individuals with possible sub-clinical diseases.

## Introduction

Reference intervals (RIs) play an important role in guiding the interpretation of laboratory results. However, several factors influence RIs with the notable sources of variation being age, sex, race, body mass index (BMI) and ethnicity [1,2]. Use of inappropriate RIs can result in misclassification of patients and subsequent mismanagement [3]. For this reason, it is recommended that laboratories determine RIs that are appropriate for the population they serve or at the very least verify any proposed RIs [4].

Few studies have been carried out in Africa to derive RIs and these have been done mainly while conducting HIV related clinical trials since adopting inappropriate RIs may result in unnecessary exclusion of potential trial volunteers and makes assessment of laboratory adverse events difficult. It is known that there are racial differences in complete blood count (CBC) parameters. Beutler *et al*. clearly demonstrated that African-Americans had lower haemoglobin (Hb), haematocrit (Hct), white blood cell count (WBC) and absolute granulocyte count compared to European-Americans but higher lymphocyte counts [5]. Karita *et al*. carried out a study to determine RIs for common haematology and chemistry tests in Kenya, Uganda, Zambia and Rwanda. The study found lower Hct, Hb, WBC and neutrophil counts compared to RIs from the United States (US) while eosinophil counts were found to be higher [6]. A similar study was carried out in Kericho, a rural area situated in the highlands of the Great Rift Valley in Kenya. The derived RIs for males and females were 8.3–11.3g/dL and 5.9–10.0 g/dL respectively. A number of participants were found to have relatively low mean corpuscular volume (MCV) and haemoglobin (MCH), values consistent with iron deficiency especially in women of child bearing age. Lower neutrophil and higher eosinophil counts compared to Caucasians were also found [7]. A genetic deletion of the Duffy antigen receptor for chemokines (DARC-null genotype), a receptor for *Plasmodium vivax*, is thought to contribute to the benign neutropenia seen in Africans and African-Americans [8]. The eosinophilia is thought to be related to increased exposure to environmental allergens and possibly parasite infections but there isn’t strong evidence to support this hypothesis.

These studies demonstrate the need to establish population specific RIs. However, establishment of RIs should follow a rigorous process of identifying reference individuals, standardization of sample handling and analysis as well as the use of appropriate statistical methods [9]. Despite all attempts to ensure that only healthy individuals are included in a RI study, this can never be achieved due to the presence of subclinical disease in subjectively healthy individuals. Reference individuals are also quite heterogeneous in their state of health and there isn’t a perfect standard to define health or normality [10]. Statistical methods such as latent abnormal values exclusion (LAVE) have been devised in order to secondarily exclude healthy individuals participating in an RI study but who have multiple related laboratory test values outside their RIs suggesting the possibility of sub-clinical diseases [11].

A criticism of RI studies especially those done in economically disadvantaged areas is that the recruited population may not necessarily be representative of an ideal reference population. There are different motivators to volunteer in a study and these can result in a selection bias hence compromising the external validity of the study results [12,13]. We set out to determine RIs for complete blood counts (CBCs) in Kenya using data from carefully selected healthy individuals recruited as part of a multicenter, multi-country, global RI study conducted by the Committee of Reference Intervals and Decision Limits (C-RIDL) under the auspices of the International Federation of Clinical Chemistry (IFCC). Further, we compared our RIs with those derived from similar studies in sub-Saharan Africa (SSA).

## Methods

### Setting

The study was carried out in Kenyan urban towns located in the counties of Nairobi, Thika, Kiambu, Nakuru and Kisii. Recruitment was largely done from colleges, universities, churches, hospitals, corporations and shopping malls. Awareness of the study was created through use of posters, flyers, emails, church announcements and engaging administrators in the various organizations.

### Recruitment

A strict recruitment criteria was used when enrolling individuals for the study as stipulated in the published study protocol and standard operating procedures [9]. Briefly, subjectively healthy black African adults aged 18–65 years who had undergone an overnight fast were recruited between January and October 2015. No financial inducements were given, however, snacks were provided to those who participated. Recruitment was stratified into 4 age groups: 20–29, 30–39, 40–49 and 50–65 years with a similar distribution of males and females in each age strata. Exclusion criteria included individuals with a body mass index (BMI) greater than 35 kg/m^2^, consumption of ethanol greater than or equal to 70 g per day [equivalent to 5 alcoholic drinks], smoking more than 20 tobacco cigarettes per day, taking regular medication for a chronic disease (diabetes mellitus, hypertension, hyperlipidemia, allergic disorders, depression), recent (less than 15 days) recovery from acute illness, injury or surgery requiring hospitalization, known carrier state of Hepatitis B, Hepatitis C or HIV, pregnant or within 1 year after childbirth. Written informed consent was sought from each participant after giving a written and verbal explanation of the study. Individuals with any chronic disease were excluded except for the age group 50–65 years where individuals with well controlled hypertension taking no more than 2 drugs were recruited. All participants had measurements of blood pressure (BP), abdominal circumference and BMI done.

### Pre-analytical sample handling

CBC samples were collected by a trained phlebotomist using an evacuated tube system comprising a sterile multi-sample needle, needle holder and plastic evacuated tubes containing ethylene diamine tetra-acetic acid (EDTA) (BD Vacutainer^®^ Blood Collection Tube, US). The samples were transported at refrigerated temperature and analyzed within 12 hours after collection. Serum and plasma samples requiring centrifugation were spun within 4 hours after collection and stored at -80°C until shipment on dry ice to the reference laboratory in South Africa for analysis of tests that were part of the global RI study. All samples arrived in the reference laboratory frozen and were only thawed once prior to sample analysis.

### Sample analysis

All the sample analysis for the biochemistries and immunoassays were performed in the PathCare reference laboratory in Cape Town, South Africa, which is an International Organization for Standardization (ISO) 15189 accredited laboratory. All participating countries in Africa had their general biochemistry and immunoassays done in this laboratory so as to standardize analysis, ensure alignment and comparison of results by using a common panel of sera across all participating laboratories globally. The CBC parameters included red blood cell count (RBC), Hb, Hct, MCV, MCH, mean corpuscular hemoglobin concentration (MCHC), red cell distribution width (RDW), WBC, platelet (PLT), leukocyte differential counts of neutrophil (Neu), lymphocyte (Lym), monocyte (Mon), eosinophil (Eos), basophil (Bas). The differential counts were recorded as both % of WBC and absolute count (abs). The CBC was analysed using a Beckman Coulter ACT 5 DIFF CP analyser (Brea, California, US) in the PathCare Kenya laboratory based in Nairobi which is also ISO 15189 accredited by the South African National Accreditation Service (SANAS).

Iron (Fe), transferrin (Tf), albumin (Alb), and ultrasensitive c-reactive protein (hsCRP) were analysed using a Beckman Coulter AU5800 analyser while ferritin levels were determined using a Beckman Coulter DXI analyser. The globulin fraction (Glb) was calculated by subtracting Alb from total protein (TP). These non-CBC parameters were used as part of reference tests in the LAVE procedure for secondary exclusion of individuals with latent anemia or inflammation.

### Ethics approval

The study was approved by the Aga Khan University Hospital Nairobi, Health research ethics committee (2014/REC-46) and was carried out in accordance with the Declaration of Helsinki.

### Statistical analysis

The sample size from each country participating in the global RI study was set at a minimum of 500 (male and female: 250 × 2), so that country-specific RIs would be obtained in a more reproducible manner. This number was deemed adequate to make between-country comparisons of test results with a power of detecting a difference of two means equivalent to 0.25 times the standard deviation comprising the RI (SD _RI_), which corresponds to a bias of 0.25 times between-individual variation, allowing errors of α less than 0.05 and β less than 0.2 in the statistical hypothesis testing done separately for each sex. According to the CLSI guideline [4], at least 120 individuals are required to determine RIs using non-parametric methods for each defined population group and for parameters not influenced by sex (60 females and 60 males). For parameters that are influenced by sex, the minimum recommended number is 240 reference individuals (120 females and 120 males). Therefore, the sample size of 500 well exceeds what is recommended by the CLSI and allows derivation of RIs with higher precision (narrower 90% confidence intervals of the RI limits, or LL and UL). For comparative purposes, RIs were determined using both parametric and non-parametric methods before and after applying the LAVE method as described by Ichihara *et al*. [14,15]. Briefly, for the non-parametric method, the reference values coinciding with the 2.5^th^ and 97.5^th^ percentiles after arranging the data in ascending order were used to identify the RI lower and upper limits (LL and UL). For the parametric method, the data was transformed into a Gaussian form by using a modification of the original Box-Cox power transformation formula as described by Ichihara [16], ascertaining the mean and SD, and finally determining the RI as the mean ± 1.96SD, which corresponds to the central 95% limits or LL and UL under transformed scale. Then, the limits are reverse transformed to get the LL and UL in the original scale.

The LAVE method is an iterative optimization procedure which is applied in a situation where mutually related analytes were tested simultaneously. Initially, RIs are determined for each analyte independently using either a parametric (mean ± 1.96 SD after Gaussian transformation) or non-parametric (mid 95% range) method. From the second computation, any individual who has two or more results outside the RIs derived in the previous computation among the reference tests (see below) is excluded. This process was repeated six times at which point the RIs were nearly stable. Of note is that need for exclusion of a reference value is determined using a set of pre-defined reference tests but not the test whose RI is being determined [11]. In this study we chose the following 11 analytes as the reference tests: Alb, Glb, hsCRP, Fe, Tf, ferritin, Hb, Hct, MCV, WBC, and PLT which can help identify individuals with latent anemia and/or inflammation.

For judging the need to partition reference values by sex and age, we computed the standard deviation ratio (SDR) which represents a ratio of between-subgroup SD (variation of the subgroup means from grand mean) to between-individual SD (approximately 1/4 the width of RI). We performed 2-level nested ANOVA to compute between-sex SD and between-age group SD after partitioning age as 18–29 years, 30–39 years, 40–49 years and 50–65 years. The SDR for between-sex SD (SDRsex) and for between-age SD (SDRage) were computed as a ratio to residual SD (or between-individual SD). Since between-age variation changes by sex, we also computed SDRage for each sex by one way ANOVA. We considered SDR ≥ 0.40 as a guide for judging the need for partitioning reference values by sex or age [11]. For analysing the relationship between Hb and PLT, we performed least-square linear regression analysis and the degree of the association was expressed by Pearson’s correlation coefficient. We quantified the magnitude of change in RI lower limit (LL) and upper limit (UL) before and after applying the LAVE procedure using the formulas SDR-LL = |LL+ − LL-|/ {(UL+ − LL+)/3.92} and SDR-UL = |UL+ − UL-|/ {(UL+ − LL+)/3.92} where LL+, UL+ are the RI limits with application of LAVE, while LL-, UL- are RI limits without application of LAVE [17]. We set 0.3 as a critical value to define a significant change in LL or UL after application of LAVE. Analysis was carried out using a general purpose statistical software, StatFlex version 6.0 (Artech Inc., Osaka, Japan).

## Results

### 1. Volunteers recruited

**Fig 1:**
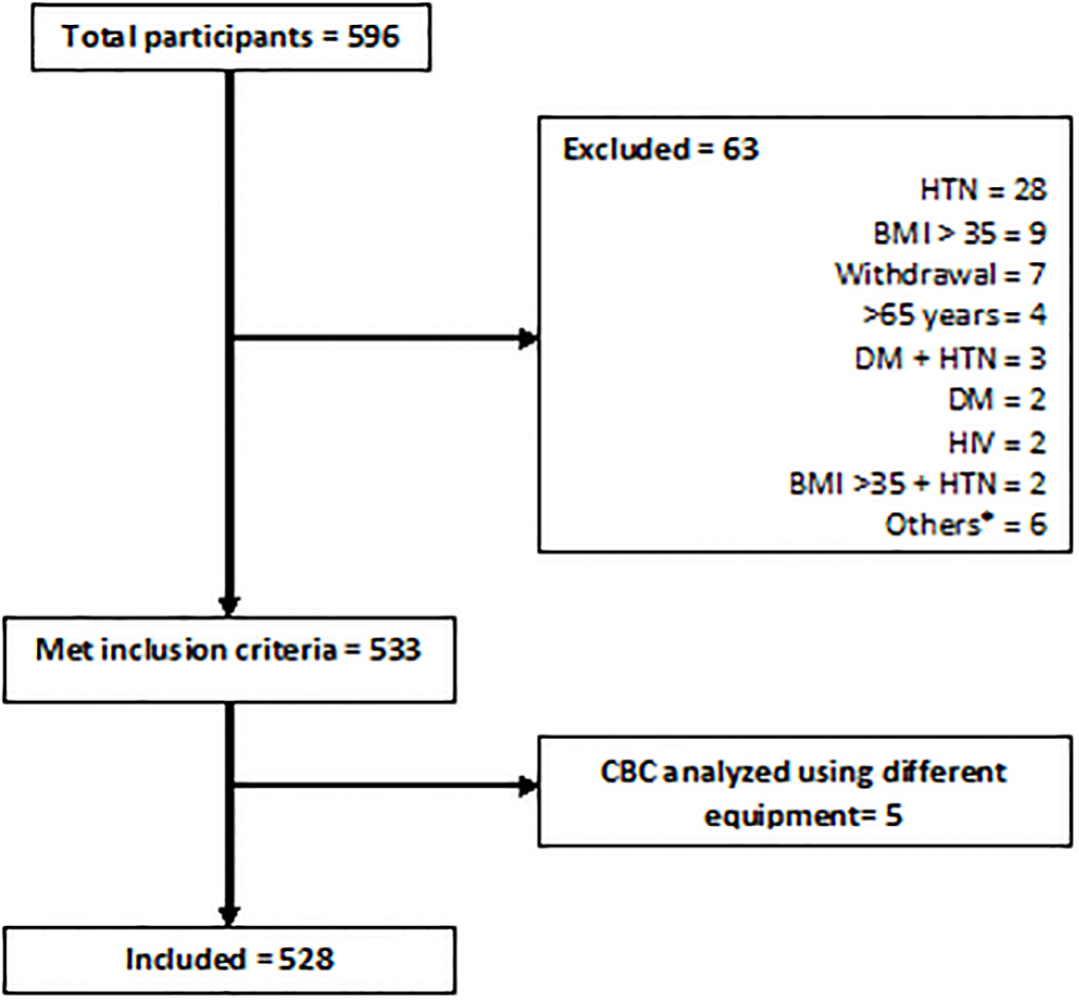
Flow diagram for recruitment. BMI: body mass index, CBC: complete blood count, DM: diabetes mellitus, HIV: Human Immunodeficiency Virus, HTN: hypertension *On antibiotics, blood donation in past 3 months, on treatment for hypothyroidism, > 65 years, > 65 years and hypertensive, rheumatic heart disease, prostate cancer.

A total of 596 individuals volunteered to participate in the study out of which 63 were excluded for various reasons as shown in Fig 1. Out of 533 eligible participants, only 528 had CBC results available with 254 (48.1%) being males. Five individuals were excluded because CBCs were performed at a different laboratory using different equipment.

The median age for the included participants was 39 years with the youngest and oldest being 18 and 65 years respectively as shown in Table 1. The number of participants in the four age groups were 134 (18–29 years), 136 (30–39 years), 131 (40–49 years) and 127 (50–65 years). The number of smokers was low with only 2 (0.7%) and 13 (5.1%) women and men respectively.

**Table 1:** Descriptive characteristics of participants (Median, 2.5~97.5 percentile).

Parameter	Male (n = 255)	Female (n = 273)	Total (n = 528)
Age (years)	38.0 (21.0~64.0)	39.0 (20.0~61.7)	39.0 (20.0~63.0)
BMI (kg/m^2^)	24.9 (18.3~33.6)	26.1 (18.5~34.9)	25.5 (18.3~34.2)
SBP (mmHg)	81.0 (61.0~99.0)	79.0 (61.0~98.7)	80.0 (61.0~99.0)
DBP (mmHg)	127.0 (103.9~151.3)	118.0 (96.3~156.0)	123.5 (98.7~155.0)

BMI: body mass index, DBP: diastolic blood pressure, SBP: systolic blood pressure

### 2. Sex and age related changes in reference values

Using a cut-off of 0.4, SDRs revealed that sex was a significant source of variation for RBC count, Hb, Hct, PLT count, absolute monocyte count and percentage while age was a significant source of variation only for absolute monocyte count and percentage in females as shown in Table 2. For all other CBC parameters, age and sex did not appear to be major sources of variation hence we chose not to partition their RIs.

**Table 2:** Complete blood count standard deviation ratios for age and sex.

Item	SDR-sex	SDR-age	SDR-age M	SDR-age F
RBC	**1.02**	0.05	0.08	0.00
Hb	**1.28**	0.10	0.15	0.00
Hct	**1.28**	0.14	0.22	0.00
MCV	0.00	0.00	0.00	0.17
MCH	0.04	0.00	0.00	0.10
MCHC	0.19	0.00	0.00	0.15
RDW	0.13	0.00	0.00	0.00
WBC	0.00	0.13	0.12	0.14
Neu %	0.34	0.16	0.18	0.14
Lym %	0.16	0.08	0.19	0.00
Mon %	**0.46**	0.39	0.33	**0.53**
Eos %	0.13	0.14	0.14	0.13
Bas %	0.27	0.00	0.00	0.05
Neu Abs	0.22	0.00	0.00	0.04
Lym Abs	0.00	0.19	0.21	0.17
Mon Abs	0.33	**0.42**	0.34	**0.53**
Eos Abs	0.12	0.17	0.22	0.07
Bas Abs	0.20	0.08	0.00	0.20
PLT	**0.41**	0.00	0.00	0.00

%: Percentage, RBC: red blood cell count, Hb: haemoglobin, MCV: mean corpuscular volume, MCH: mean corpuscular haemoglobin, MCHC: mean corpuscular haemoglobin concentration, RDW: red cell distribution width, WBC: white blood cell count, Neu: neutrophil, Lym: lymphocyte, Mon: monocyte, Eos: eosinophil, Bas: basophil, Abs: absolute count, Plt: platelet count, SDR: standard deviation ratio, M: male, F: female. SDRs > 0.4 are in bold

**Fig 2:**
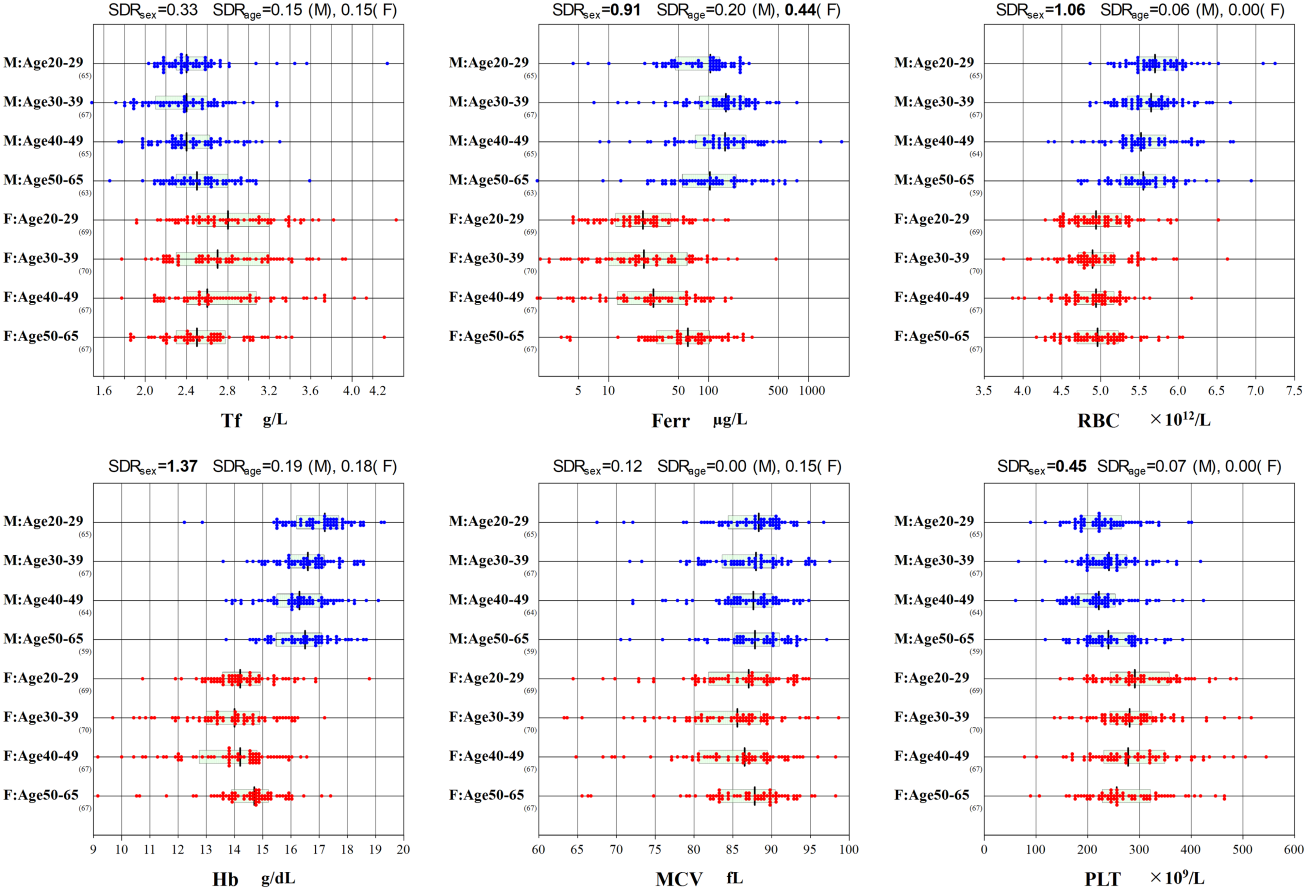
Sex and age-related changes for representative parameters. F: female, Ferr: ferritin, Hb: haemoglobin, M: male, MCV: mean corpuscular volume, Plt: platelet count, RBC: red blood cell count, SDR: standard deviation ratio, Tf: transferrin.

Reference values (RVs) of Hb, Hct and RBC were considerably lower in female participants compared to males while PLT counts were higher in female participants. For the non CBC parameters, RVs of serum iron and ferritin were lower in female participants but those of transferrin were higher as shown in Fig 2.

**Fig 3:**
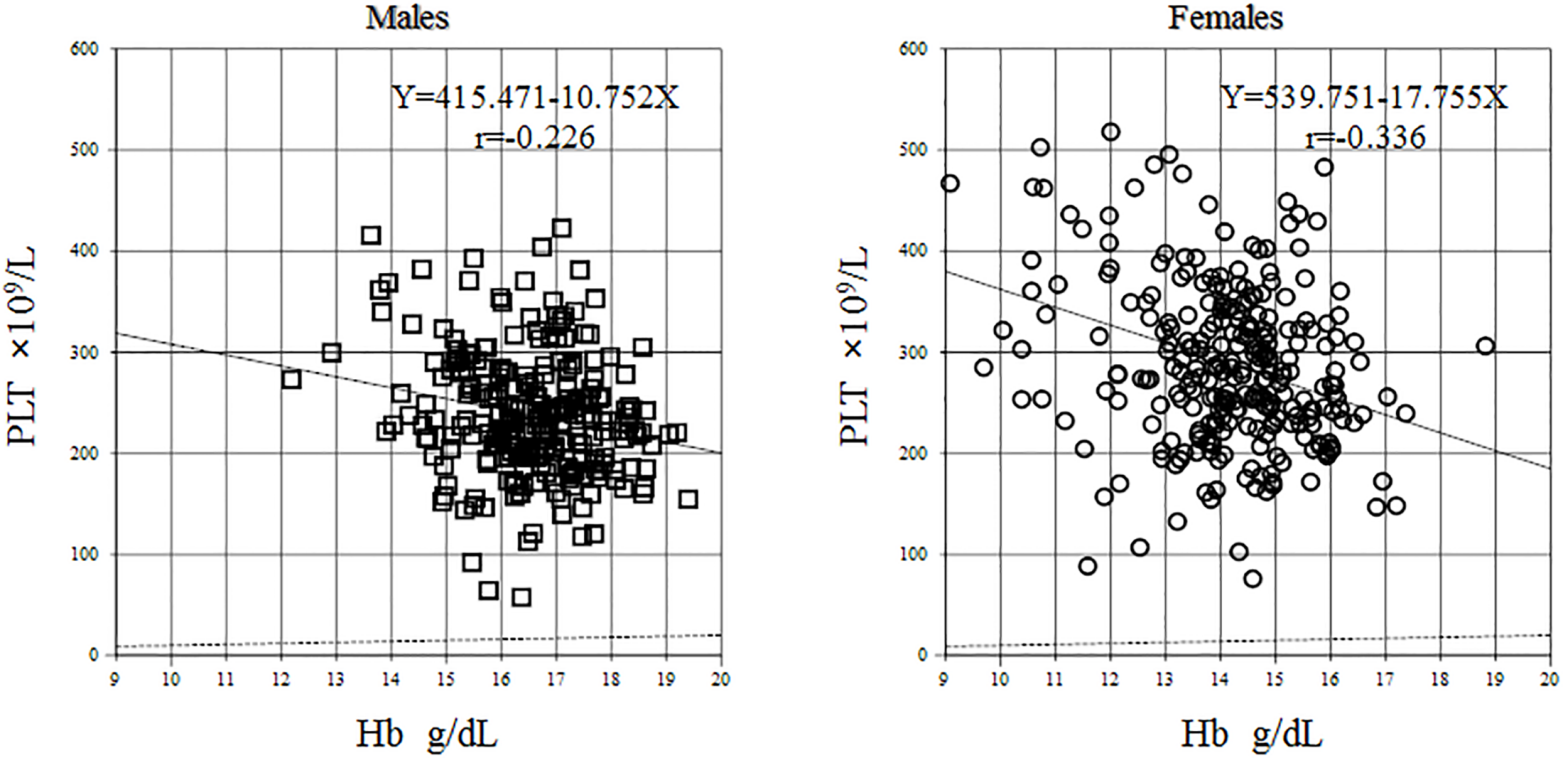
Relationship between platelet count and haemoglobin level. Hb: haemoglobin, Plt: platelet.

There was a negative correlation between Hb level and platelet count with the magnitude of the correlation coefficient being greater in females (r = −0.336) than compared to males (r = −0.226) as shown in Fig 3.

### 3. Derivation of reference intervals

**Fig 4:**
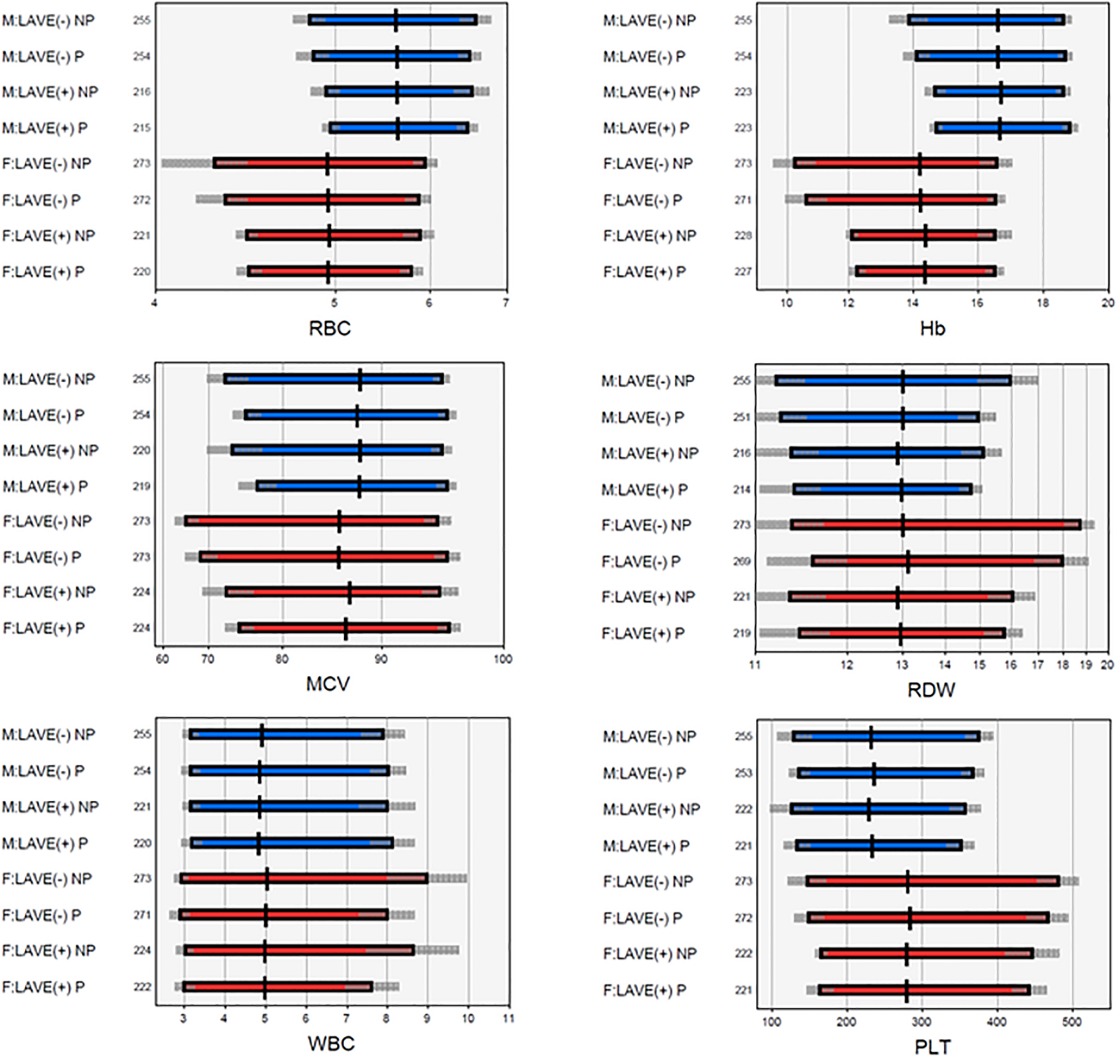
Distribution of reference values and reference intervals for selected CBC parameter. F: female, Hb: haemoglobin, LAVE: latent abnormal value exclusion, M: male, MCV: mean corpuscular volume, Plt: platelet count, RBC: red blood cell count, RDW: red cell distribution width, WBC: white blood cells.

Without application of the LAVE method, RIs derived using the non-parametric method generally showed wider ranges and 90% CIs of the RI limits compared to the parametric method. This tendency was more prominently seen in female participants especially for RBC, Hb, Hct, MCV, MCH, RDW and PLT as shown in the graphical representation of RIs in Fig 4 and S1 Fig.

The LAVE procedure had a greater effect in females where it significantly raised the LLs and narrowed the RIs for Hb, Hct, MCV, MCH and MCHC compared to males where the LAVE resulted in a minor change in the LLs of RBC parameters. S1 Table shows the SDRs for the change in LLs and ULs after application of the LAVE method. An SDR greater than 0.3 was considered a significant change in LL or UL by use of the LAVE method. On the other hand, the LAVE method didn’t significantly alter LLs for WBC parameters but reduced ULs for absolute basophil and neutrophil counts.

The parametrically derived female RI for Hb before applying the LAVE method was 10.6–16.5 g/dL but changed to 12–16.5 g/dL after applying the method. In males, the LAVE method changed the parametrically derived Hb RI from 14.2–18.7 g/dL to 14.5–18.7 g/dL. RIs for RBC related parameters were consistently lower in females as shown in Table 3. RIs for PLT were consistently higher in females with or without the LAVE method as shown in Table 3, S2 Fig and S2 Table. The RIs that we chose to adopt based on whether sex was a major source of variation are highlighted in bold in Table 3.

**Table 3:** Parametric complete blood count reference intervals after latent abnormal value exclusion.

	Male + Female		Male		Female	
Item	N	LL- UL	N	LL-UL	N	LL-UL
RBC (x10^12/L)	463	4.41–6.48	226	**4.94–6.52**	229	**4.31–5.76**
Hb (g/dL)	470	12.8–19.0	232	**14.5–18.7**	236	**12.0–16.5**
Hct (L/L)	471	0.38–0.55	232	**0.43–0.55**	237	**0.36–0.49**
MCV (fl)	466	**75.7–95.6**	228	76.5–95.5	232	73.4–95.8
MCH (pg)	462	**24.8–32.8**	227	25.1–32.8	230	24.4–32.7
MCHC (g/dL)	461	**32.2–35.2**	227	32.4–35.4	230	32.0–35.0
RDW (%)	460	**11.3–15.2**	225	11.3–14.7	228	11.4–15.8
WBC (x10^9/L)	464	**3.08–7.83**	229	3.13–8.10	232	2.89–7.72
Neu (%)	463	**28.0–63.3**	227	27.4–60.3	230	29.5–65.4
Lym (%)	463	**27.2–60.0**	227	28.2–60.3	230	25.5–59.3
Mon (%)	461	3.4–13.3	226	**3.5–14.3**	229	**3.2–11.0**
Eos (%)	455	**1.1–11.9**	225	1.2–11.8	227	0.8–9.4
Bas (%)	456	**0.30–1.10**	224	0.40–1.20	228	0.30–1.00
Neu (x10^9/L)	460	**1.05–4.08**	227	1.02–3.92	229	1.07–4.42
Lym (x10^9/L)	461	**1.29–3.40**	226	1.36–3.58	230	1.22–3.24
Mon (x10^9/L)	462	0.14–0.74	227	**0.15–0.76**	229	**0.14–0.68**
Eos (x10^9/L)	460	**0.04–0.59**	226	0.05–0.64	228	0.04–0.49
Bas (x10^9/L)	461	**0.01–0.07**	226	0.01–0.08	224	0.01–0.06
PLT (x10^9/L)	464	144–409	231	**133–356**	232	**152–443**

%: percentage, LL: lower limit, UL: upper limit, RBC: red blood cell count, Hb: haemoglobin, MCV: mean corpuscular volume, MCH: mean corpuscular haemoglobin, MCHC: mean corpuscular haemoglobin concentration, RDW: red cell distribution width, WBC: white blood cell count, Neu: neutrophil, Lym: lymphocyte, Mon: monocyte, Eos: eosinophil, Bas: basophil, Abs: absolute count, Plt: platelet count. Recommended RIs are shown in bold.

**Fig 5:**
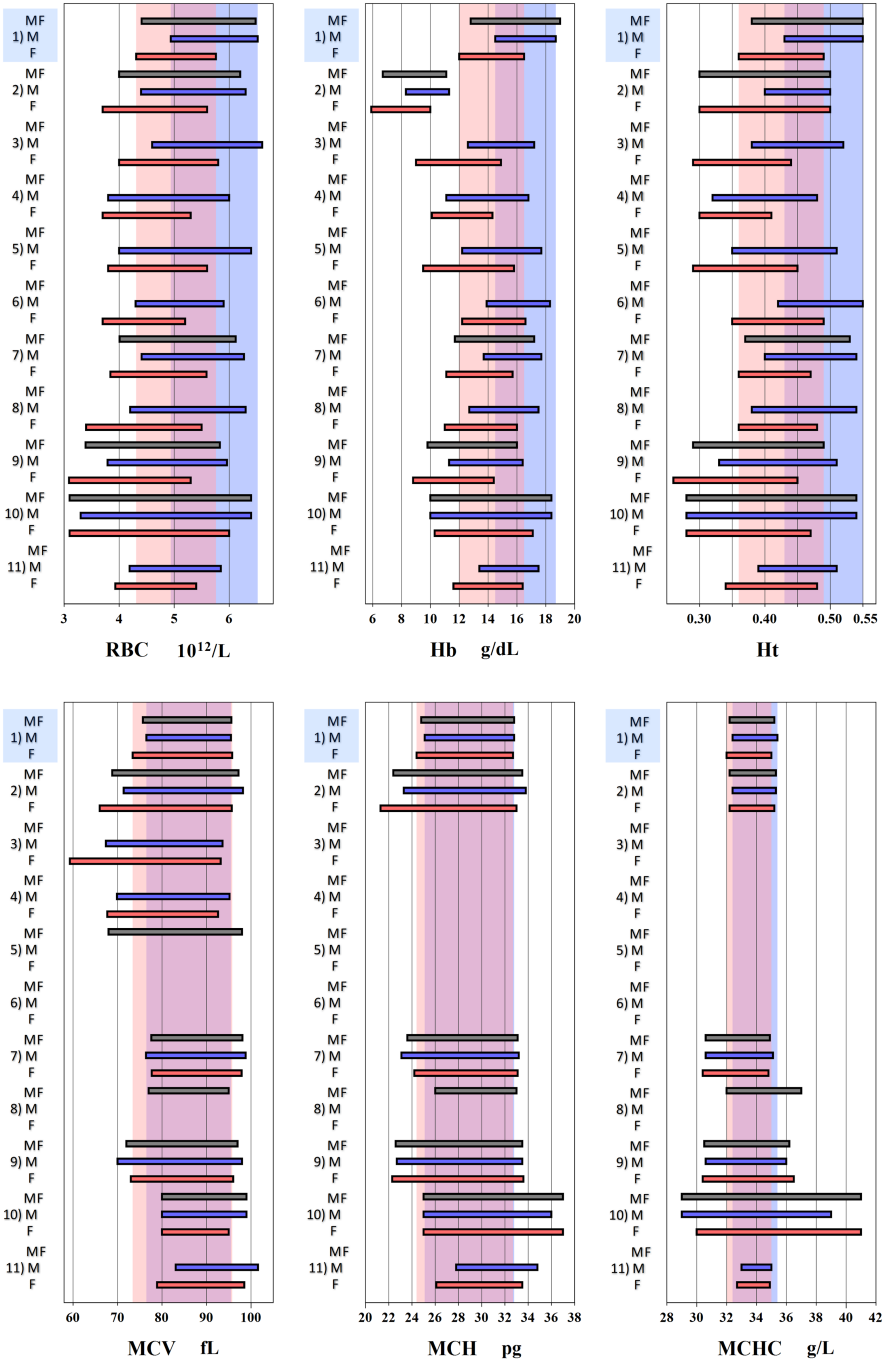
Graphical comparison of reference intervals (RIs) for RBC related parameters. Three bars for each study represent RIs for male+female (MF) in grey, male (M) in blue, and female (F) in red. RIs of this study is shown on top of each panel. The background shades in blue and pink represent RIs of this study for males and females. LL: lower limit, UL: upper limit, RBC: red blood cell count, Hb: haemoglobin, Ht: haematocrit, MCV: mean corpuscular volume, MCH: mean corpuscular haemoglobin, MCHC: mean corpuscular haemoglobin concentration ^1^**Exclusion criteria**: BMI > 35 kg/m^2^, consumption of ethanol ≥ 70 g per day, smoking more than 20 tobacco cigarettes per day, chronic illness, recent recovery from acute illness, injury or surgery requiring hospitalization, known carrier state of HBV, HCV or HIV, pregnant or within 1 year after child birth. **CBC analyser**: Beckman Coulter ACT 5 DIFF CP analyser (Brea, California, US). ^2^**Exclusion criteria**: Febrile, pregnant, HIV seropositive, screen positive for syphilis and malaria. **CBC analyser**: ACT 5Diff CP instrument (Beckman Coulter, Fullerton, CA, USA) [7]. ^3^**Exclusion criteri**a: HIV positive, pregnant. **CBC analyser**: Coulter ACT 5Diff CP analyser (Beckman Coulter, France) [18]. ^4^**Exclusion criteria**: HIV positive, moribund, mentally ill, institutionalized persons, missing personal or laboratory data. **CBC analyser**: Act 5 Diff instrument (Beckman Coulter) [19]. ^5^**Exclusion criteria**: Acutely ill, significant findings on physical examination or if laboratory tests revealed that they were pregnant, HIV antibody positive, had evidence of hepatitis B or C infection or suspected syphilis. **CBC analyser**: Beckman Coulter AcT 5 diff CP (Beckman Coulter, USA) [6]. ^6^**Exclusion criteria**: HIV positive, presence of any illness as defined by the World Health Organization staging systems for HIV infection and disease. **CBC analyser**: Coulter counter T540 [20]. ^7^**Exclusion criteria**: HIV positive, pregnant or on medication, body temperature ≥37.5°C or if clinical assessment revealed other signs or symptoms of disease that could influence the laboratory parameters of interest. **CBC analyser**: Sysmex KX-21N analyser (Sysmex Corp., Kobe, Japan) [21]. ^8^**Exclusion criteria**: Alcohol abuse, medication, smoking, pregnant, breastfeeding, on oral contraception, on menses. **CBC analyser**: Coulter AcT 5diff and Sysmex KX-21N (Sysmex Corporation, Kobe, Japan) [22]. ^9^**Exclusion criteria**: Acute or chronic respiratory, cardiovascular, gastrointestinal, hepatic or genitourinary conditions, blood donation or transfusion within the past 3 months, hospitalisation within past 1 month, any findings that would compromise laboratory parameters, pregnant or lactating mothers. **CBC analyser**: Micros 60 analysers (Horiba-ABX, Montpellier, France) [23]. ^10^**Exclusion criteria**: HIV, HBV, and HCV viral infection, malaria, abnormal haemoglobin electrophoresis screening, presence of hypochromia. **CBC analyser**: Sysmex SF-3000 (Sysmex, Kobe, Japan) [24]. ^11^**Exclusion criteria**: HIV, current clinical symptoms, immunosuppressive or corticosteroid medication, chemotherapy, hospitalizations, surgery or blood transfusions in the six months prior to screening, splenomegaly, pregnant, Hb < 12 g/dL. Menstruating women returned in 2 weeks. **CBC analyser**: Beckman Coulter LH 750 (Beckman Coulter, Fullerton, CA, USA) [25].

**Fig 6:**
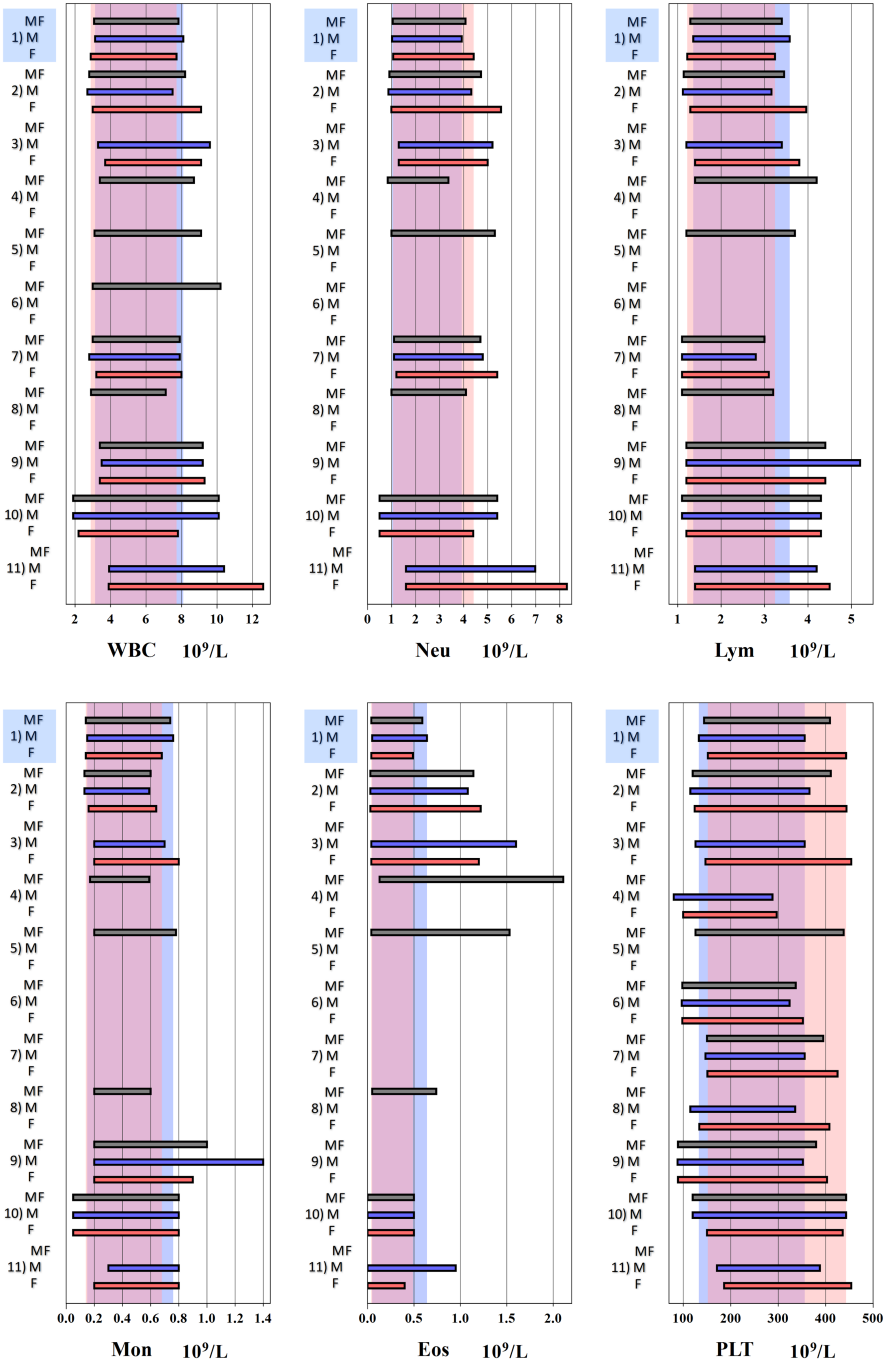
Graphical comparison of reference intervals (RIs) for WBC related parameters and platelets. Three bars for each study represent RIs for male+female (MF) in grey, male (M) in blue, and female (F) in red. RIs of this study is shown on top of each panel. The background shades in blue and pink represent RIs of this study for males and females. WBC: white blood cell count, Neu: Neutrophil, Lym: lymphocyte, Mon: monocyte, Eos: eosinophil, abs-absolute count, Plt-platelet count ^1^**Exclusion criteria**: BMI > 35 kg/m^2^, consumption of ethanol ≥ 70 g per day, smoking more than 20 tobacco cigarettes per day, chronic illness, recent recovery from acute illness, injury or surgery requiring hospitalization, known carrier state of HBV, HCV or HIV, pregnant or within 1 year after child birth. **CBC analyser**: Beckman Coulter ACT 5 DIFF CP analyser (Brea, California, US). ^2^**Exclusion criteria**: Febrile, pregnant, HIV seropositive, screen positive for syphilis and malaria. **CBC analyser**: ACT 5Diff CP instrument (Beckman Coulter, Fullerton, CA, USA) [7]. ^3^**Exclusion criteri**a: HIV positive, pregnant. **CBC analyser**: Coulter ACT 5Diff CP analyser (Beckman Coulter, France) [18]. ^4^**Exclusion criteria**: HIV positive, moribund, mentally ill, institutionalized persons, missing personal or laboratory data. **CBC analyser**: Act 5 Diff instrument (Beckman Coulter) [19]. ^5^**Exclusion criteria**: Acutely ill, significant findings on physical examination or if laboratory tests revealed that they were pregnant, HIV antibody positive, had evidence of hepatitis B or C infection or suspected syphilis. **CBC analyser**: Beckman Coulter AcT 5 diff CP (Beckman Coulter, USA) [6]. ^6^**Exclusion criteria**: HIV positive, presence of any illness as defined by the World Health Organization staging systems for HIV infection and disease. **CBC analyser**: Coulter counter T540 [20]. ^7^**Exclusion criteria**: HIV positive, pregnant or on medication, body temperature ≥37.5°C or if clinical assessment revealed other signs or symptoms of disease that could influence the laboratory parameters of interest. **CBC analyser**: Sysmex KX-21N analyser (Sysmex Corp., Kobe, Japan) [21]. ^8^**Exclusion criteria**: Alcohol abuse, medication, smoking, pregnant, breastfeeding, on oral contraception, on menses. **CBC analyser**: Coulter AcT 5diff and Sysmex KX-21N (Sysmex Corporation, Kobe, Japan) [22]. ^9^**Exclusion criteria**: Acute or chronic respiratory, cardiovascular, gastrointestinal, hepatic or genitourinary conditions, blood donation or transfusion within the past 3 months, hospitalisation within past 1 month, any findings that would compromise laboratory parameters, pregnant or lactating mothers. **CBC analyser**: Micros 60 analysers (Horiba-ABX, Montpellier, France) [23]. ^10^**Exclusion criteria**: HIV, HBV, and HCV viral infection, malaria, abnormal haemoglobin electrophoresis screening, presence of hypochromia. **CBC analyser**: Sysmex SF-3000 (Sysmex, Kobe, Japan) [24]. ^11^**Exclusion criteria**: HIV, current clinical symptoms, immunosuppressive or corticosteroid medication, chemotherapy, hospitalizations, surgery or blood transfusions in the six months prior to screening, splenomegaly, pregnant, Hb < 12 g/dL. Menstruating women returned in 2 weeks. **CBC analyser**: Beckman Coulter LH 750 (Beckman Coulter, Fullerton, CA, USA) [25].

A graphical representation of CBC RIs for selected studies carried out in SSA is given in Figs 5 and 6 to enable comparison with our derived RIs. A summary of the same is provided as S3 and S4 Tables.

## Discussion

Several studies carried out in Africa have reported marked differences in some CBC parameters when compared to Caucasian populations and even between different populations across Africa as shown in S3 and S4 Tables. A fairly consistent finding has been the lower RIs for absolute neutrophil counts in black African populations which is thought to be associated with the DARC-null genotype, an evolutionary adaptation thought to make Africans less susceptible to *Plasmodium vivax* infections [8]. In contrast to RIs from the US where the absolute neutrophil count RI is 1800–7700 cells/μL [26], the parametrically derived neutrophil count RI for males and females in our study was significantly lower at 1050–4080 cells/μL, which is in keeping with what has been found in other African studies [6,7,21]. Neutrophil count RIs with LLs as low as 500 and 840 cells/uL have been reported in Togo and Uganda respectively as shown in S4 Table.

Studies carried out in largely rural populations in SSA have revealed low Hb levels especially in women. A study done in Kericho which is a rural town in Kenya located in The Great Rift Valley at an altitude of over 2000 metres above sea level derived a Hb lower RI limit in women of 5.9 g/dL [7] while a study by Karita *et al*. that involved 4 countries in SSA found a LL of 9.1 g/dL [27]. It is inconceivable that a Hb of 5.9 g/dL would be accepted as a LL even if clinically one is not presenting with symptoms of anaemia. In the study by Karita *et al*., the Hb values from one of the research sites situated in a university teaching hospital in Nairobi, Kenya were excluded as they were significantly higher than the other study sites. Other than the comparatively higher altitude of Nairobi, the authors hypothesized that the Hbs of participants from Nairobi were higher as they comprised mainly medical doctors and students considered to be healthier due to better nutrition and access to healthcare. For this reason, the RVs from Nairobi were subsequently excluded in the derivation of the consensus RI. This secondary exclusion of ‘healthier’ individuals may partly explain the low RI for Hb they derived [6]. According to the World Health Organization (WHO), a woman above 15 years of age with a Hb that is less than 12 g/dl is considered anaemic [28]. Therefore, a large proportion of women recruited in a number of studies summarized in Fig 5 were anaemic and given the low RIs for MCV and MCH, the anaemia is in keeping with a microcytic hypochromic picture commonly seen in iron deficiency. In SSA iron deficiency in women is likely due to poor nutrition or blood loss as a result of menses or repeated childbirth.

We carefully recruited an urban population for our study using strict recruitment criteria, and derived a RI for Hb in women of 12.0–16.5 g/dL after application of the LAVE method. This RI for women is higher than that reported in most SSA studies but similar to published RIs from the US of 12.0–16.0 g/dL [26]. The UL for the parametrically derived RI for male Hb after LAVE was 18.7 g/dL which is significantly higher than what has been reported from most studies in SSA but similar to the ULs of 18.3 and 18.4 g/dL reported from Ethiopia and Togo respectively [20,24]. This most likely reflects our careful attempt to recruit well-defined healthy individuals and secondarily exclude individuals with possible sub-clinical diseases by use of the LAVE method. This resulted in an increase in the LLs of RBC parameters and narrowing of RIs especially for female participants. This may explain the higher LLs for these parameters in our study compared to other SSA studies that didn’t employ strict recruitment criteria or secondary exclusion. The systematic removal of individuals with possible sub-clinical disease makes our results more generalizable to an urban healthy black African population in Kenya. The high UL for Hb seen especially in males in this study could be attributed to Nairobi’s relatively high altitude of 1700 metres above sea level.

For the comparison of parametric and nonparametric methods, as shown in S2 Table, the nonparametric method generally resulted in a wider RI, and wider 90% CIs of the RI limits. It is attributable to the fact that parametric method has a step of exclusion of values outside mean±3.5SD (probability: 0.00043 or 0.043%) after Gaussian transformation, as shown by a small reduction in data size for the parametric method in most of the CBC parameters. This clearly indicates the presence of extreme values not explained by chance, at the periphery of the distribution. The recommendation by CLSI [4] that the nonparametric method be used in determining RIs was due to the difficulty of the parametric method to ensure successful Gaussian transformation by use of the Box-Cox power transformation formula. However, Ichihara K and Boyd J proved that by use of a modified Box-Cox formula with addition of transformation origin in the original formula led to a dramatic change in goodness-of-fit of the transformation [14]. Besides, the optimal value of power for Gaussian transformation predicted by the parametric method is fairly consistent analyte by analyte [15], a fact supporting the reliability of the parametric method. In fact, for the RVs of all the analytes, the parametric method successfully led to a Gaussian shape judged from values of skewness and kurtosis as well as from the linearity in the probability paper plot (S3 Fig). These are the reasons for our adoption of RIs derived by the parametric method.

A decline in Hb level was associated with an increase in platelet count. Iron deficiency anaemia (IDA) and iron deficient erythropoiesis have been associated with secondary thrombocytosis. Conversely, iron supplementation in iron deficient individuals has been shown to increase Hb levels while reducing platelet count [29]. This inverse relationship between Hb level and platelet count may explain the higher median platelet count in women in this study as well as some of the other published studies from SSA [7,21]. Schloesser *et al*. compared platelet counts in patients with IDA to healthy controls and found an average platelet count of 499,000/μL in IDA patients compared to 242,000/μL in controls [30]. The mechanism behind the relationship between Hb level and platelet count isn’t well understood. Various hypothesis have been put forward including homology between erythropoietin and thrombopoietin resulting in proliferation and differentiation of pluripotent erythroid and megakaryocyte precursors, synergistic action of erythropoietin and thrombopoietin in megakaryocyte maturation and a direct effect of iron on thrombopoiesis [31,32]. Indeed, serum iron and ferritin levels were lower in female participants in our study while transferrin levels were higher, a common finding in iron deficient states. Sex was a significant source of variation in platelet counts (SDRsex = 0.45) in this study and therefore we adopted sex specific RIs for PLT. As shown in Fig 6, women generally have higher platelet counts than men.

The UL for the parametrically derived absolute eosinophil count in our study was 590 cells/ μL which is much lower than that reported by Karita *et al*. (1530 cells/μL) and Kibaya *et al*. (1140 cells/ μL) both of whom did studies in Kenya but recruited largely a rural population [6,7]. It is possible that the higher eosinophil counts in the rural population is due to a bigger burden of parasite infection or greater exposure to environmental allergens. Karita *et al*. mentions that in a sub-study done in Lusaka-Zambia, Entebbe-Uganda and Kigali-Rwanda, up to one third of stool samples had ova or parasites possibly explaining the higher eosinophil counts [6]. Given that our study population was largely comprised of an urban working population, the prevalence of stool parasites would be expected to be low. Measurement of serum IgE levels and evaluation of stool samples would have provided objective evidence to explore this hypothesis.

We used SDRs to determine whether sex or age were significant sources of variation necessitating partitioning of RIs. This is a method widely adopted in the studies by Ichihara *et al*. and is useful when evaluating more than one source of variation, especially when any source of variation consists of more than 2 categories [1,11]. An unexpected finding was the decline in monocyte levels with increase in age for female participants (S1 Fig). With lack of reports on similar observation, no obvious explanation is forthcoming, hence further studies are required to investigate the consistency of this finding and its significance.

RIs are used in developing guidelines for defining laboratory adverse events to be used when enrolling and following up patients in a clinical trial. In many HIV clinical trials, the Division of AIDS (DAIDS), National Institute of Allergy and Infectious Disease/National Institutes of Health adverse event tables are used to evaluate toxicities [33]. As highlighted by Karita *et al*., adopting the 2004 DAIDS grading criteria would inadvertently misclassify many healthy study volunteers as having adverse events at the time of clinical trial recruitment. In the study, 319 (15.2%) individuals would have been misclassified as having a haematology laboratory adverse event which would have resulted in their unnecessary exclusion from the study [6]. Given the marked variability in certain CBC parameters across different populations, population specific RIs should be used when defining laboratory based adverse events for clinical trials. Failure to do so will not only result in inappropriate exclusion or inclusion of study participants but misreporting on the incidence of toxicities.

The major strength of this study is the careful recruitment of healthy individuals with an even distribution of age and sex. Unlike most of the other RI studies conducted in SSA, we had strict exclusion criteria and further carried out secondary exclusion which significantly reduced the influence of individuals with latent anaemia or inflammation. This increases the external validity of the results in so far as extrapolating to an adult urban black African population in Kenya is concerned.

One of the limitations of this study was that sample analysis was done only on a Beckman Coulter analyzer hence the transferability of the RIs is limited if between-analyzer bias exists in test results. Secondly, our recruitment comprised largely an urban population, the vast majority from the capital city Nairobi and surrounding counties whose socio-economic status and lifestyles are different from a rural population. Some of the RIs may therefore not be directly applicable to a rural community. Another limitation is that we relied on self-reporting of chronic illnesses including those of an infectious aetiology. It is possible that some participants may not have provided accurate information and as such may have ended up being included inadvertently. We however tried to minimize the effect of such inclusion by secondarily excluding individuals whose test results suggested the possibility of sub-clinical disease.

### Conclusion

The present study highlights marked differences in certain CBC parameters such as Hb, eosinophil and platelet counts compared to other SSA countries and lower neutrophil counts compared to the US. To the best of our knowledge this is the first study from Africa that has used the LAVE procedure to determine RIs for CBC and we do believe that the secondary exclusion of individuals with possible sub-clinical disease makes our RIs more representative of a healthy urban Kenyan population. Our study results can serve as a reference for laboratories in SSA especially in situations where conducting a formal RI study may not be feasible.

## Supporting information

S1 Fig Age and sex distribution of anaytes. (PDF)

S2 Fig Reference intervals before and after latent abnormal value exclusion. (PDF)

S3 Fig Normalization of analyte data. (PDF)

S1 Table Standard deviation ratios for change in upper and lower limits of reference intervals by use of the LAVE method. (PDF)

S2 Table Complete blood count reference intervals. (PDF)

S3 Table Summary of selected reference interval studies for erythrocyte related parameters in sub-Saharan Africa. (PDF)

S4 Table Summary of selected reference interval studies for platelets and white blood cell related parameters in sub-Saharan Africa. (PDF)
