# Supplementary figures and images for: Complete blood count reference intervals from a healthy adult urban population in Kenya
Source: PLoS One. 2018 Jun 7;13(6):e0198444. doi: 10.1371/journal.pone.0198444 (PMC5991659; doi:10.1371/journal.pone.0198444)

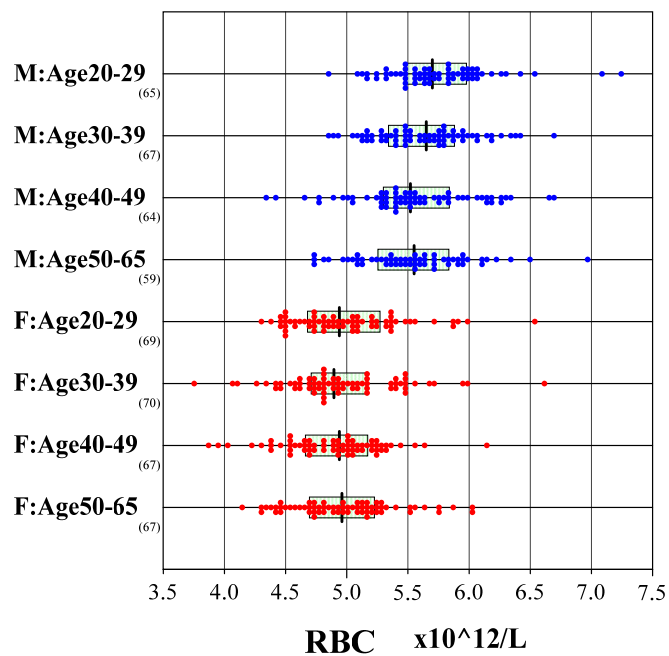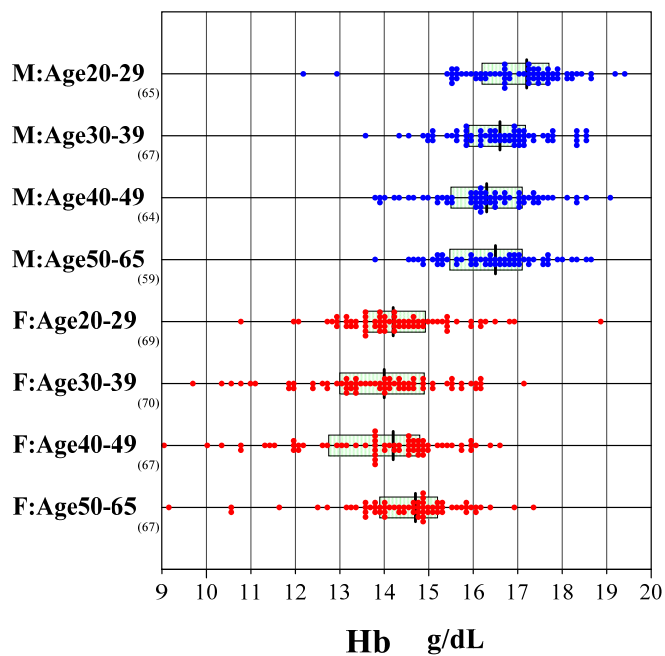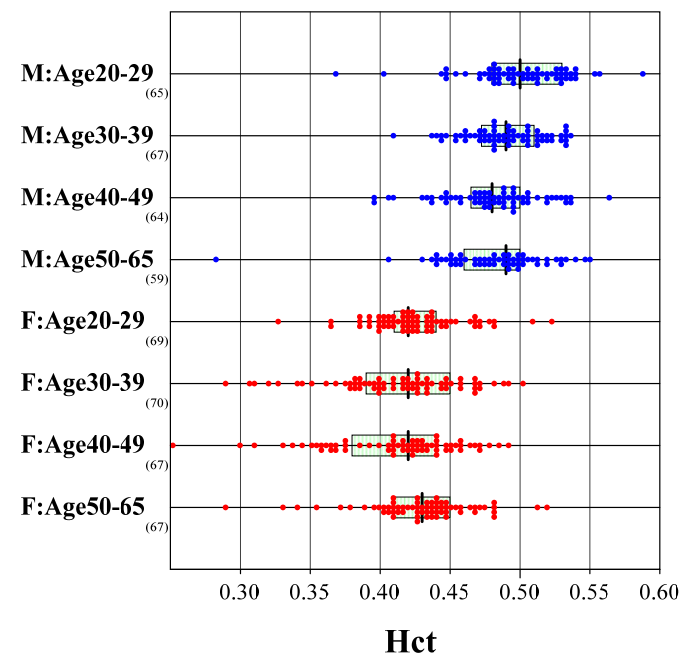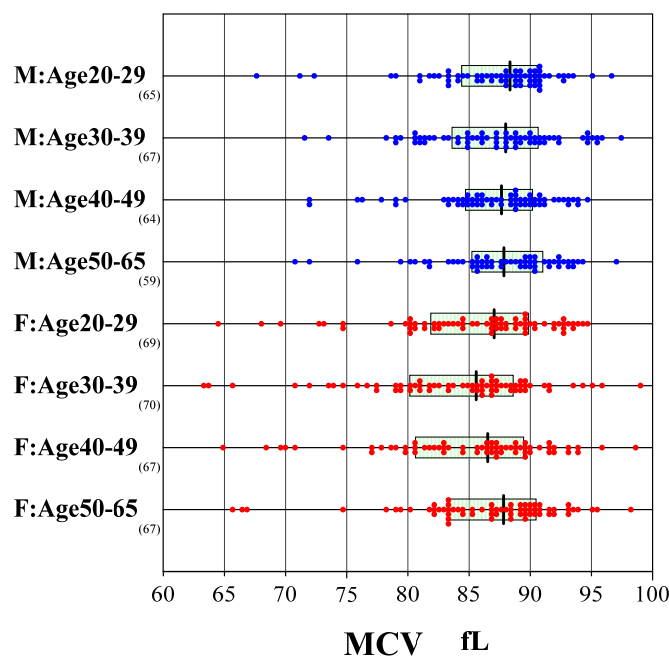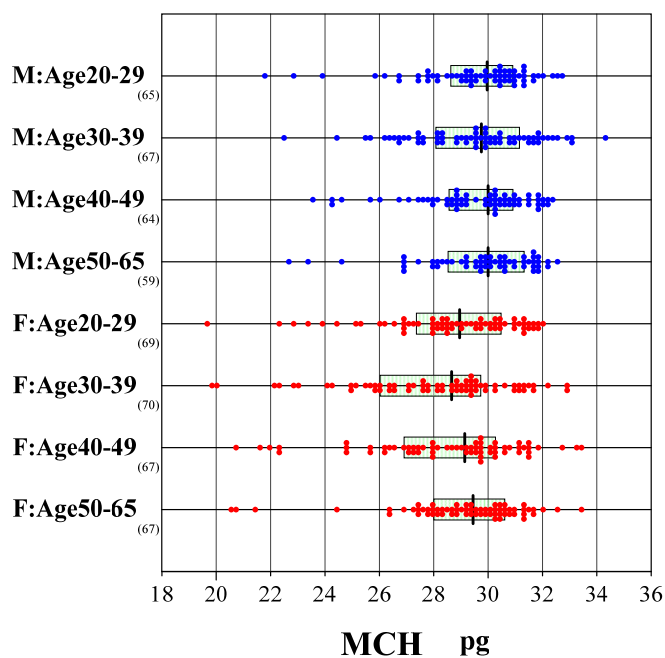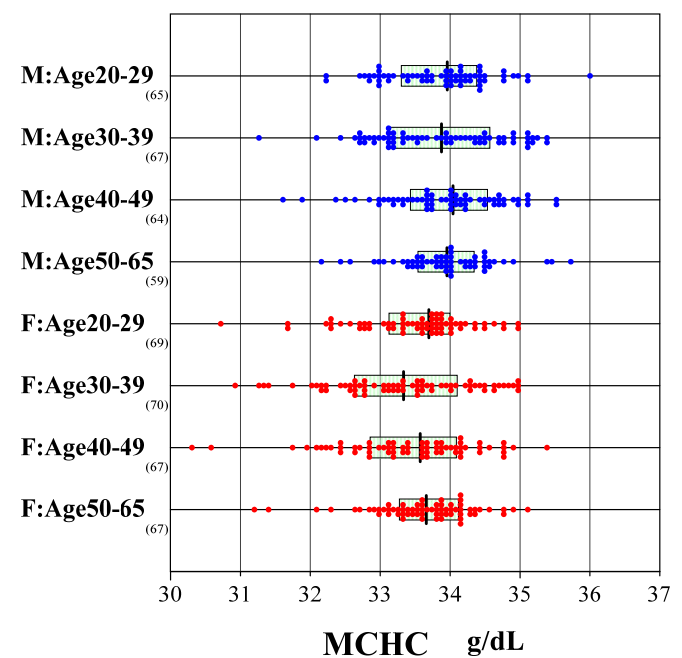

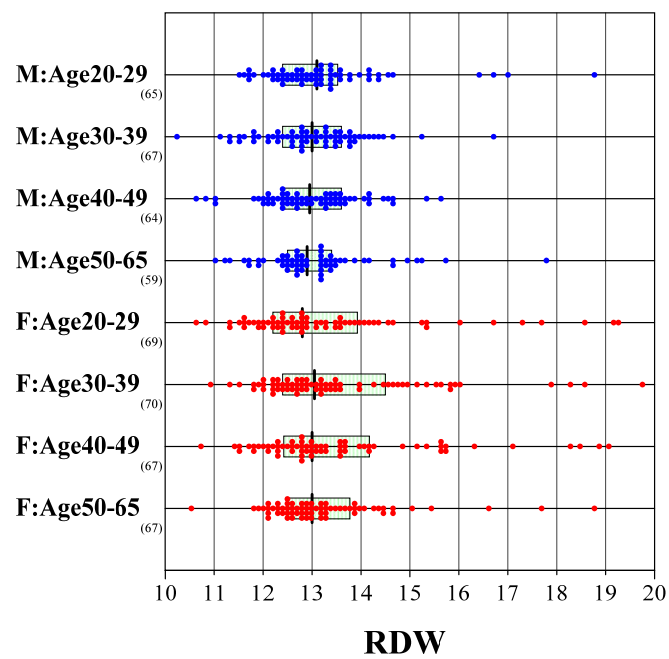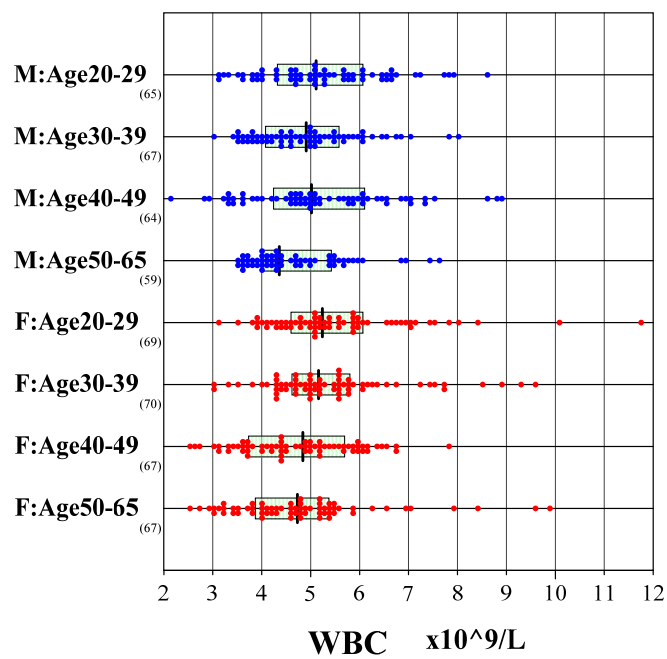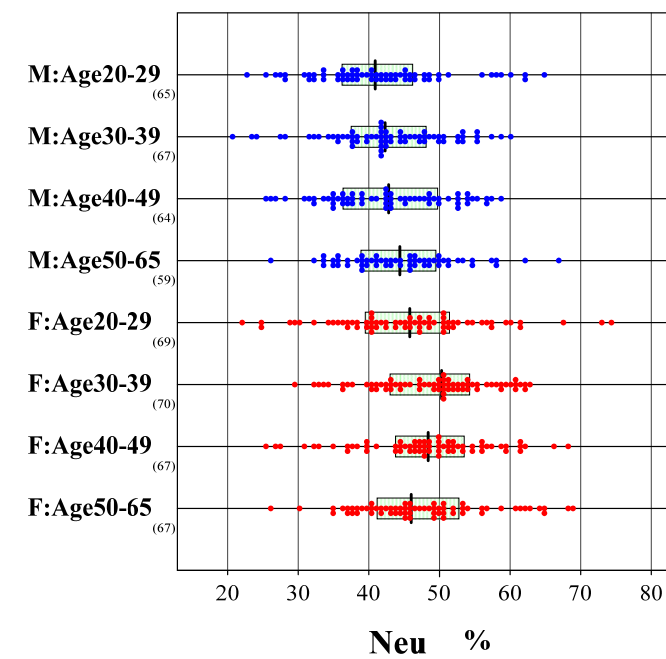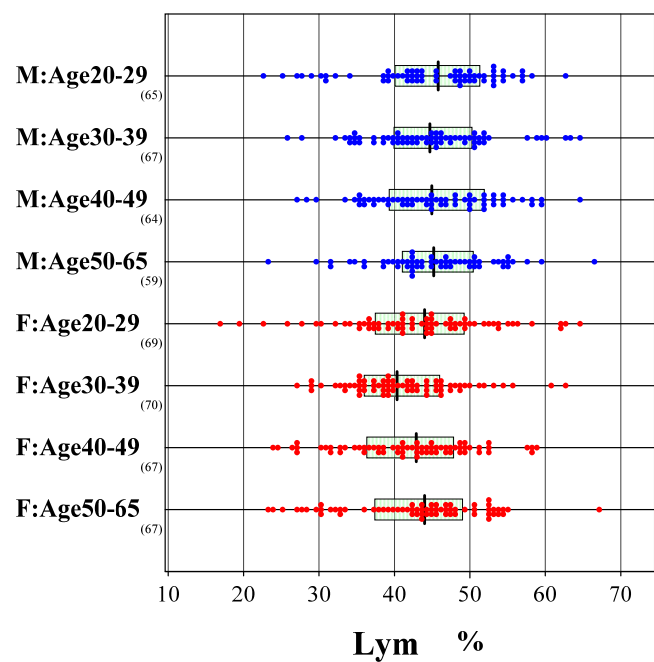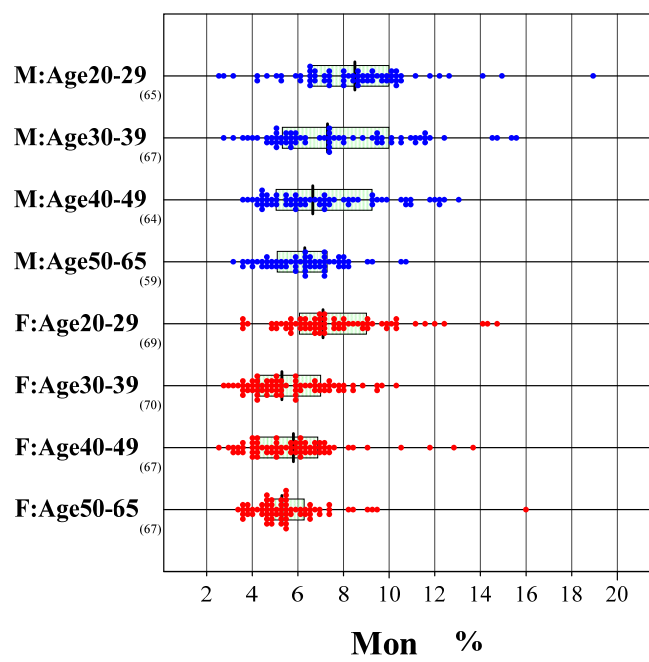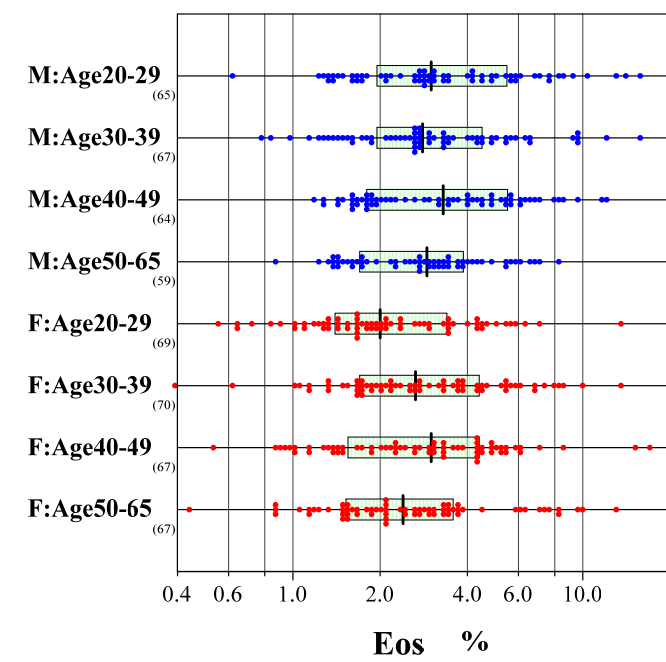

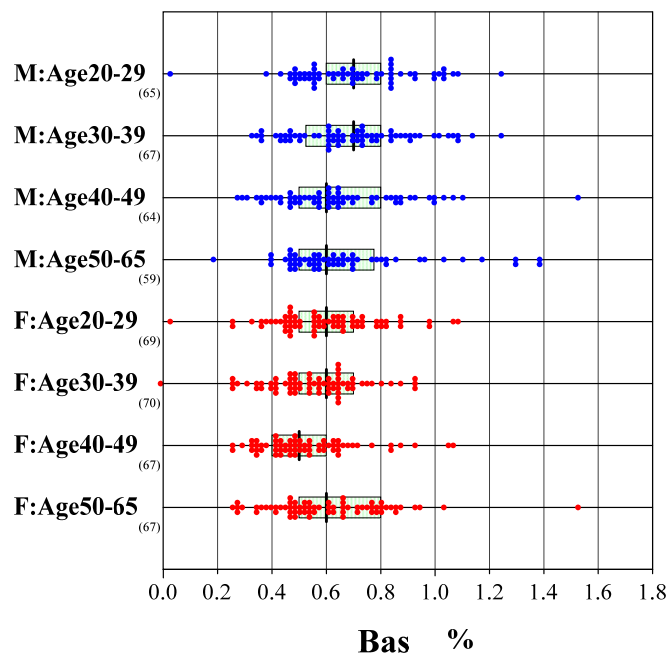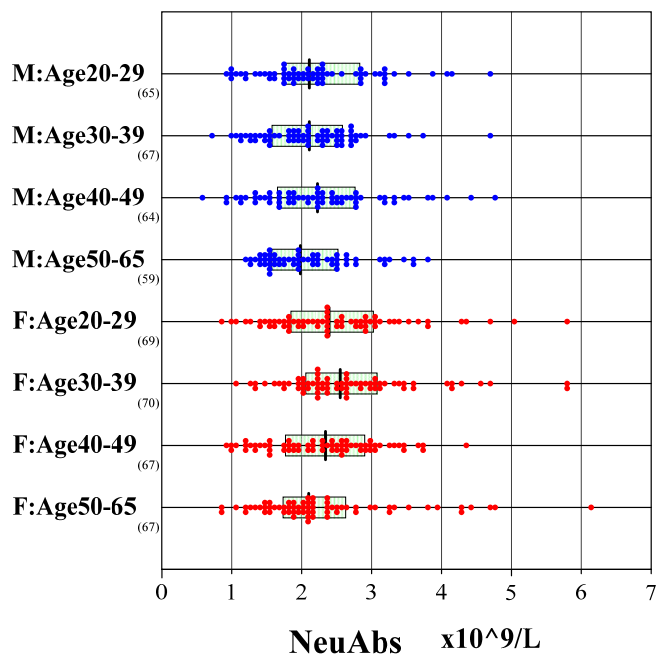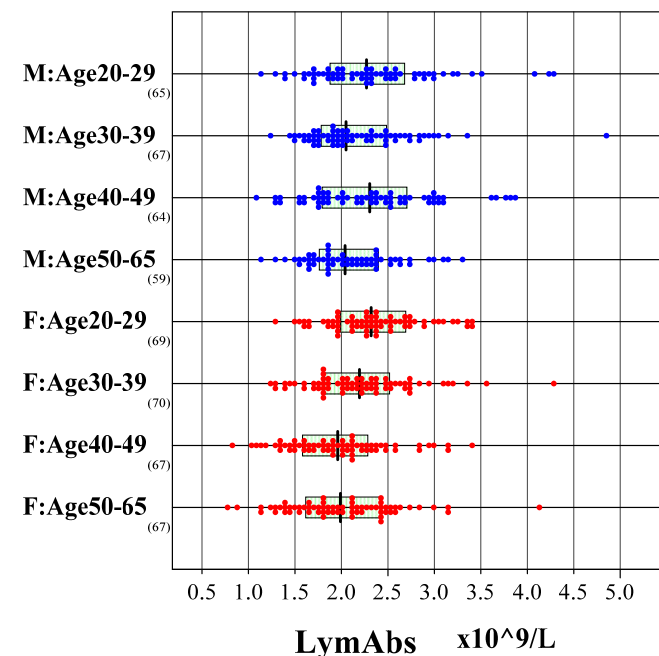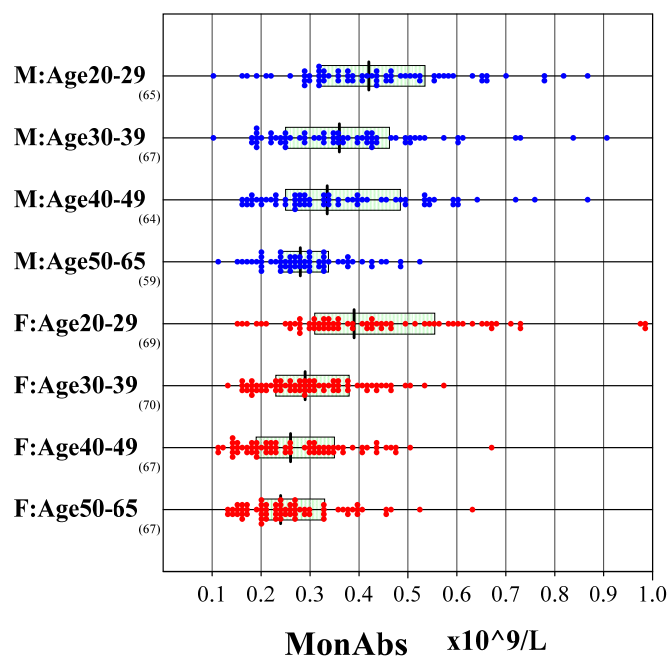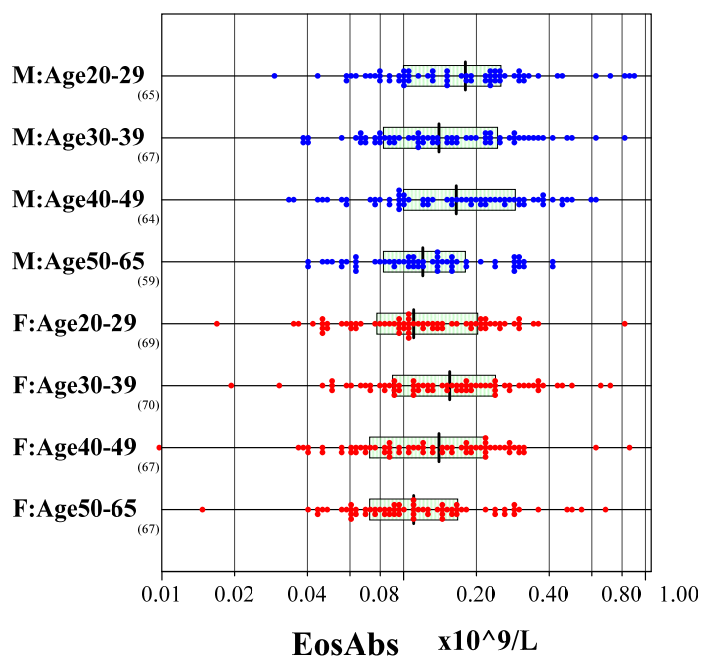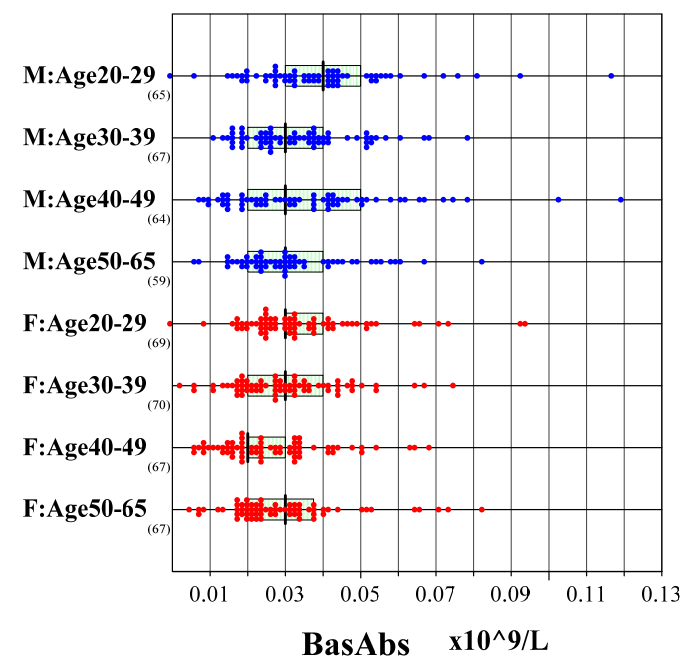

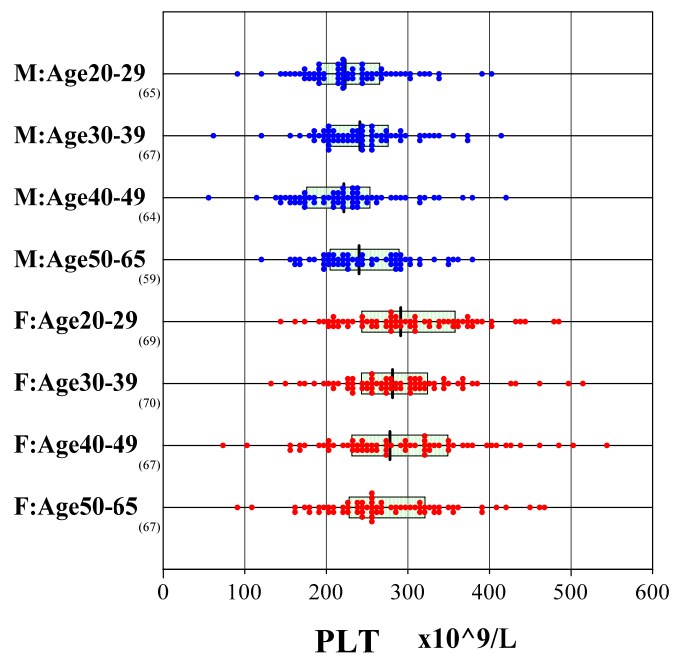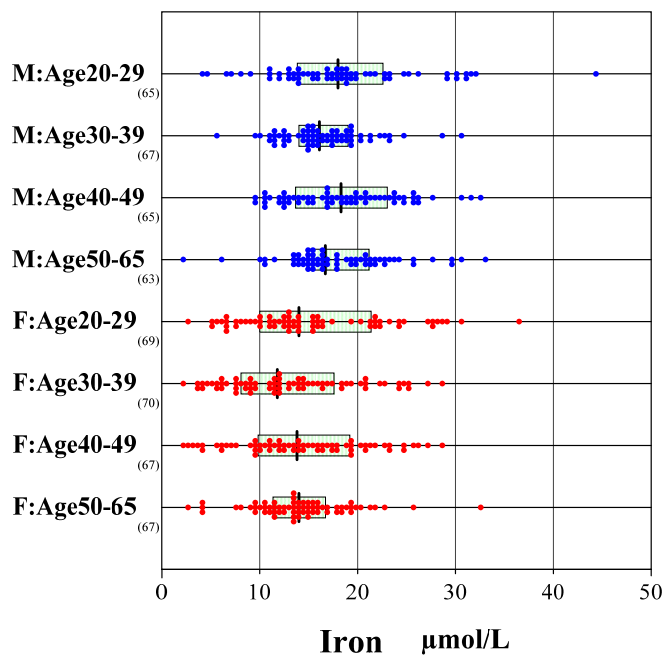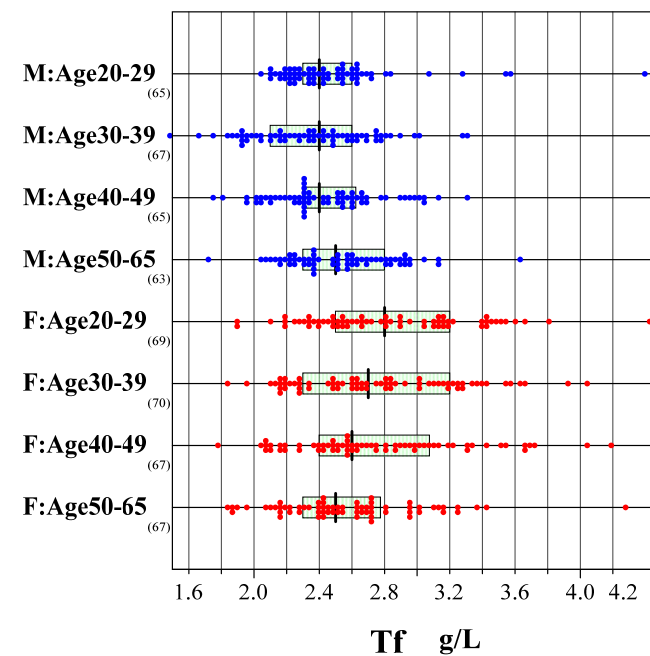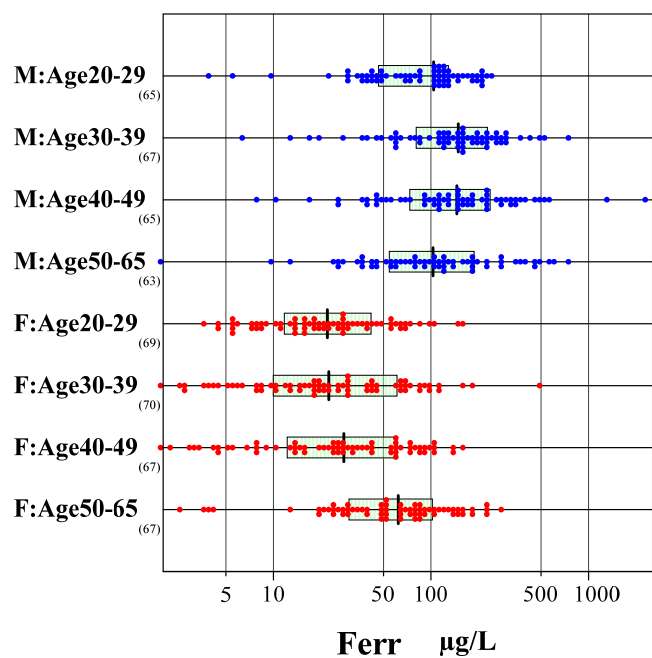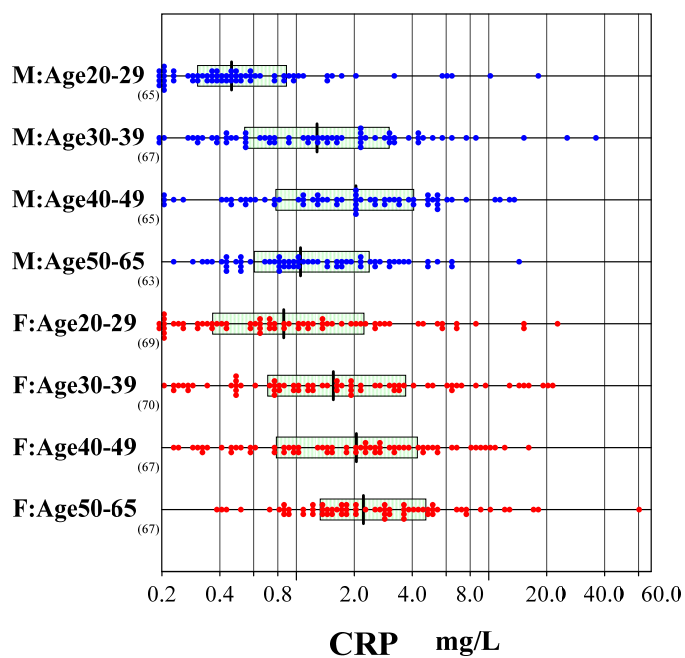

Supplement: S1 Fig — (PDF) [file pone.0198444.s001.pdf]

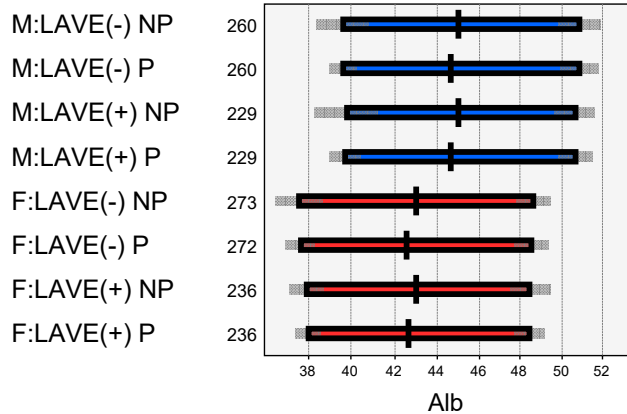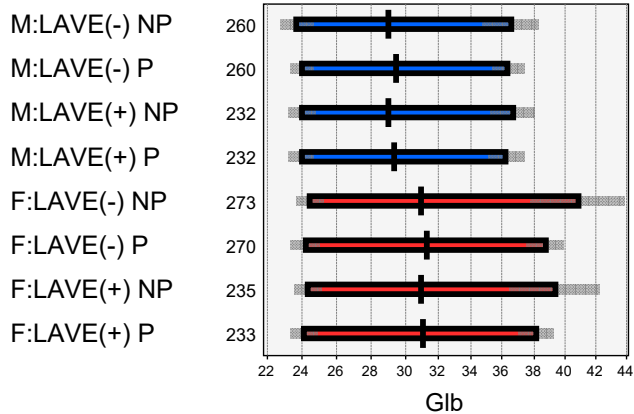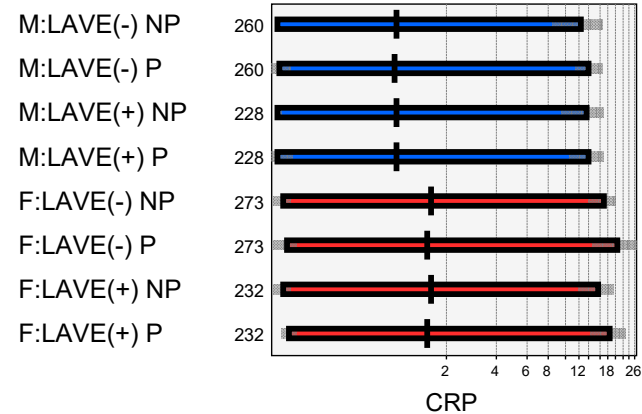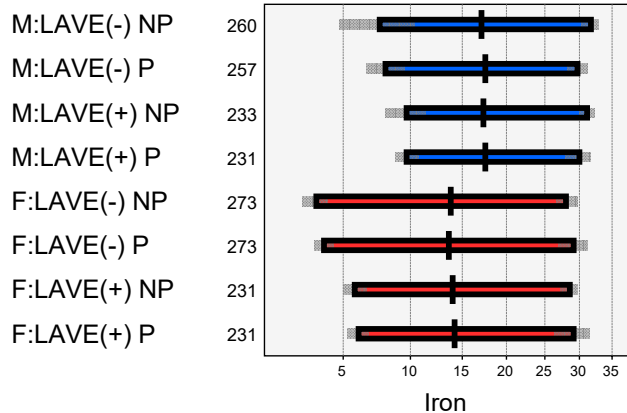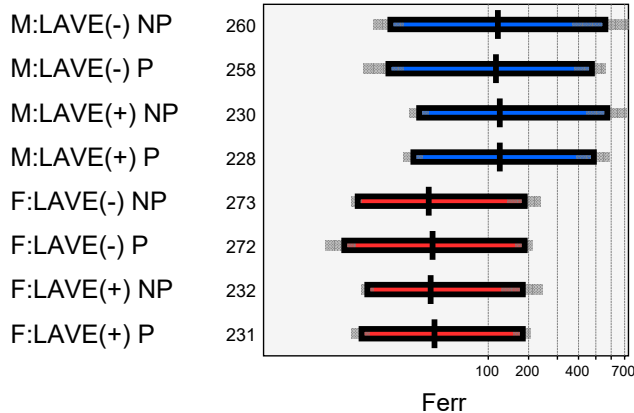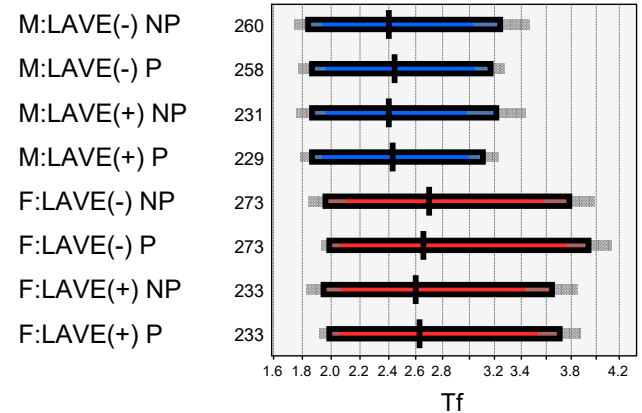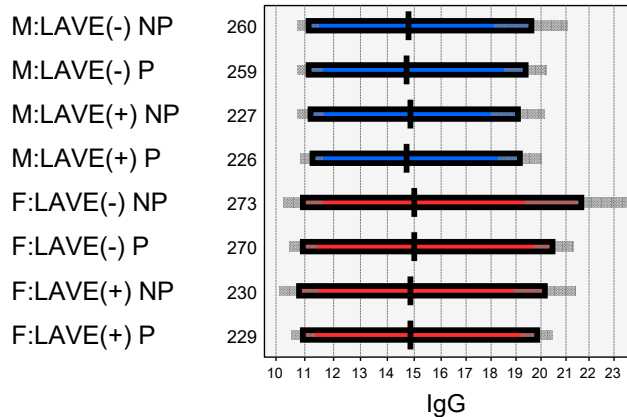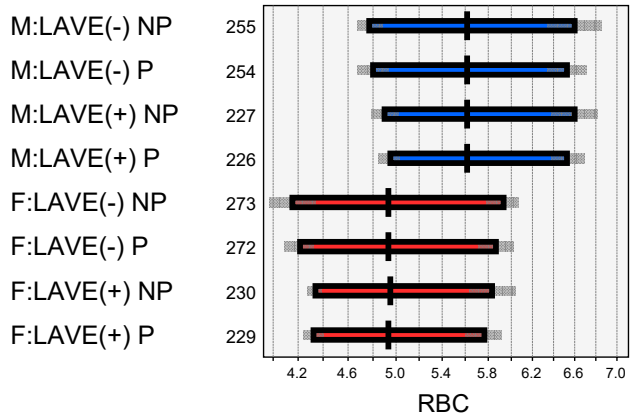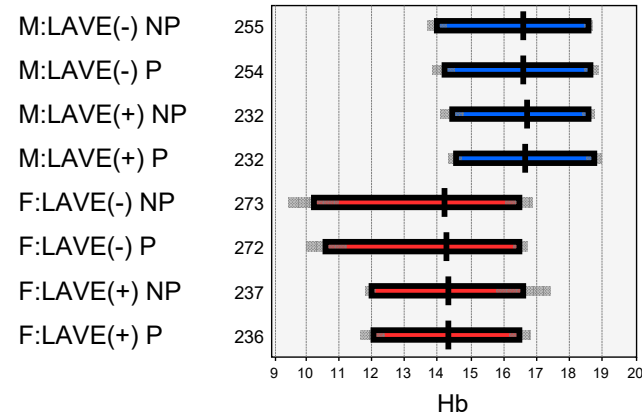

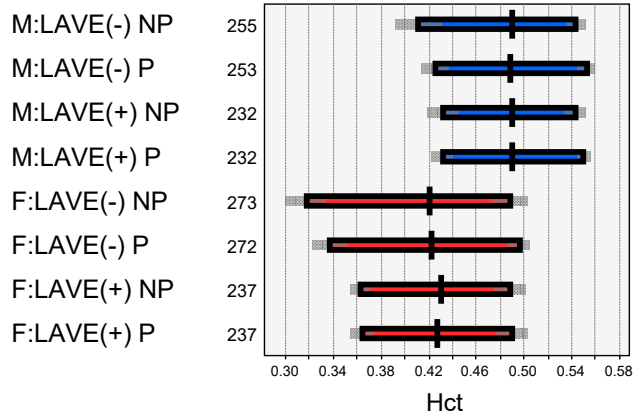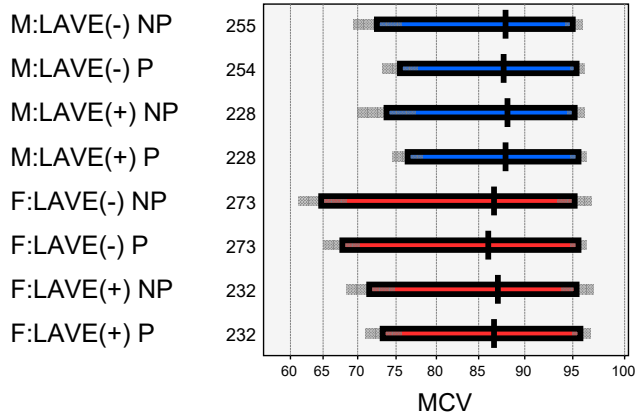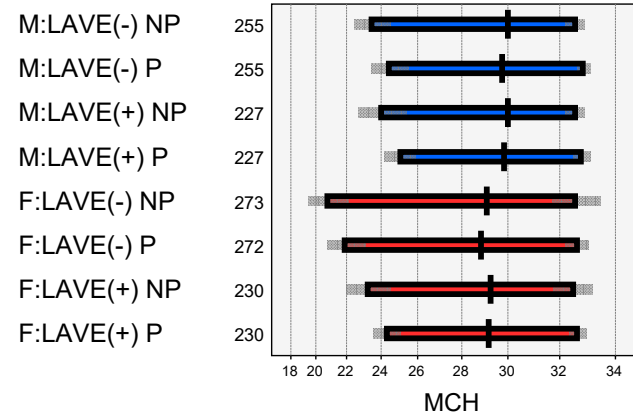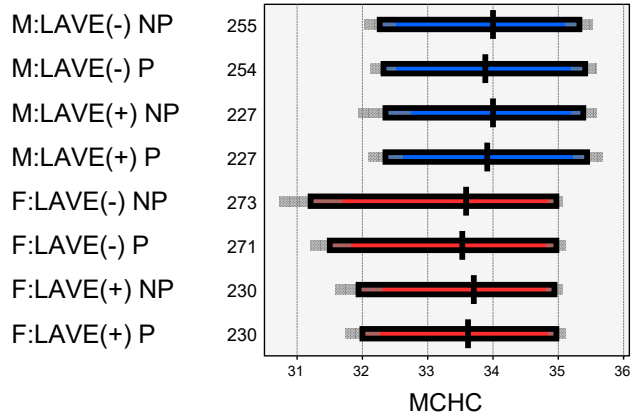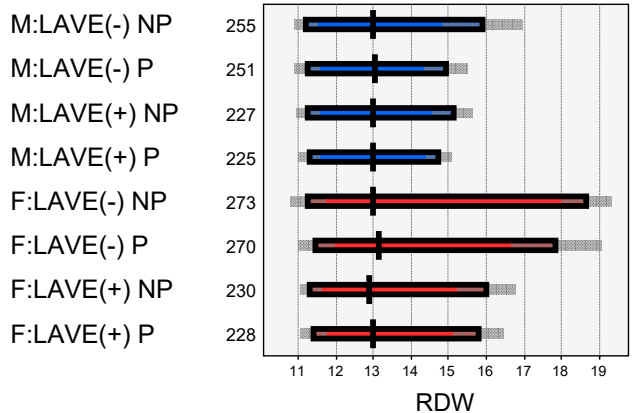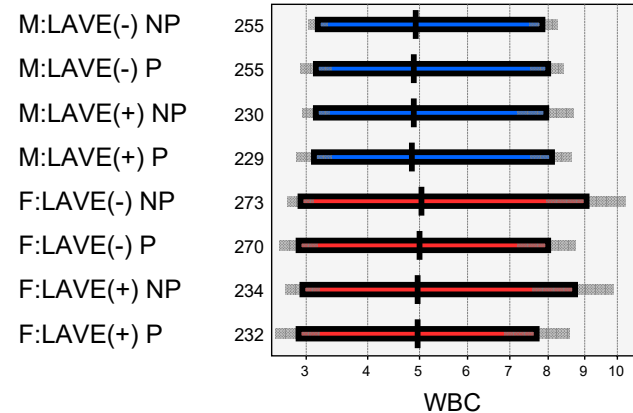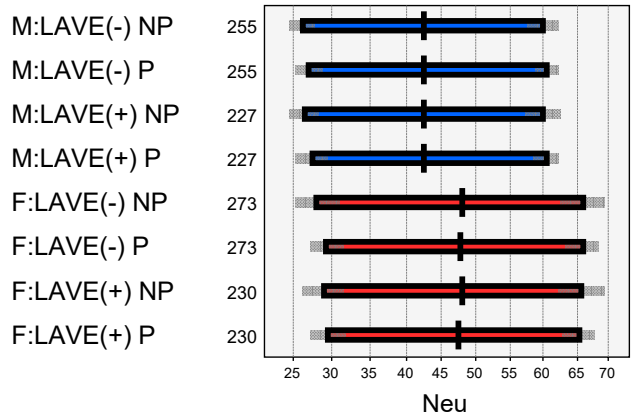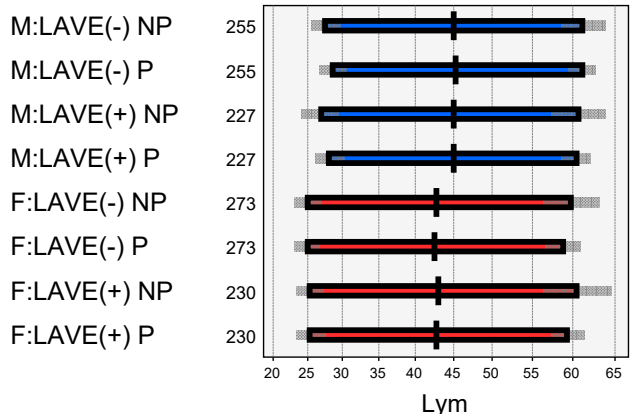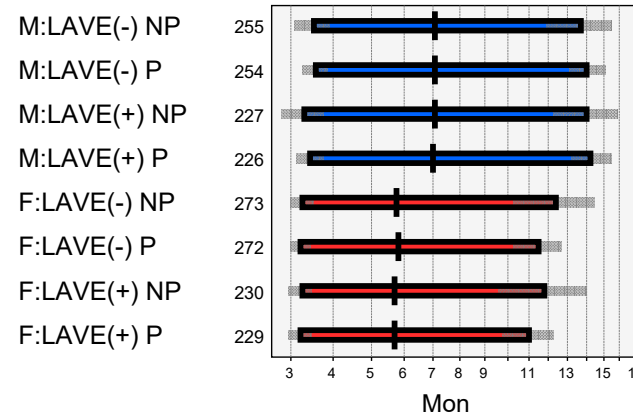

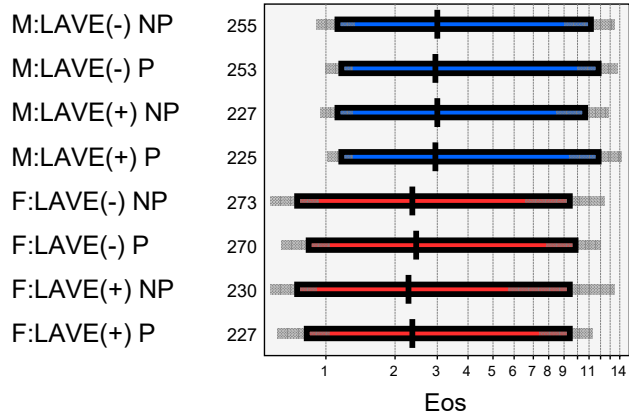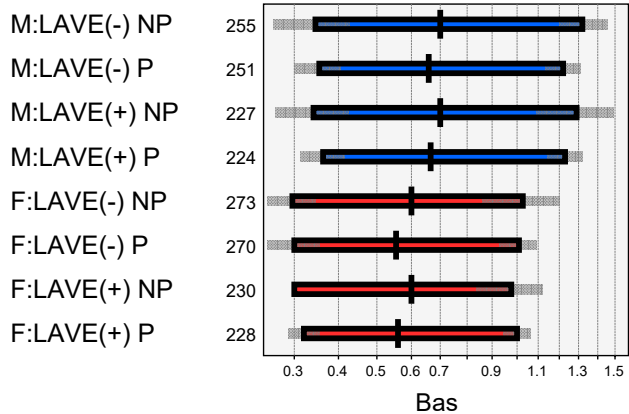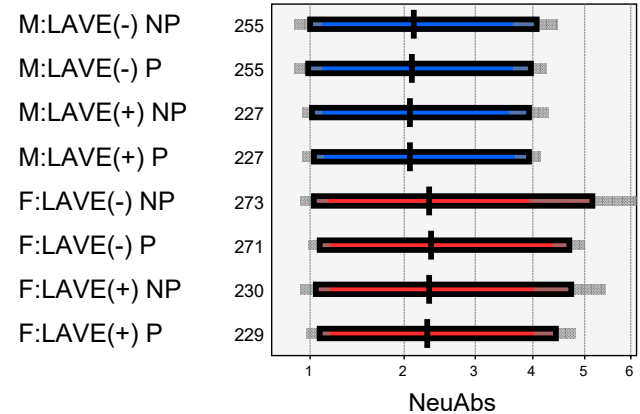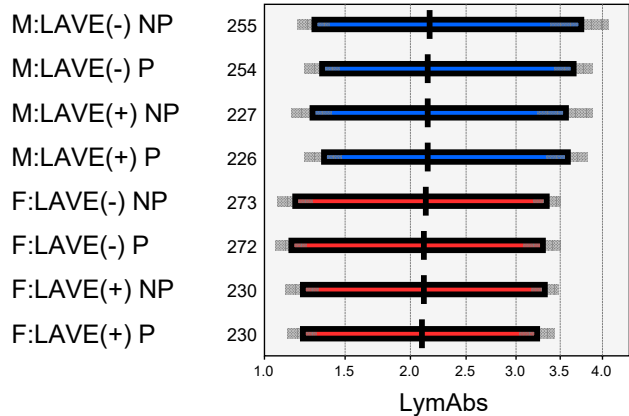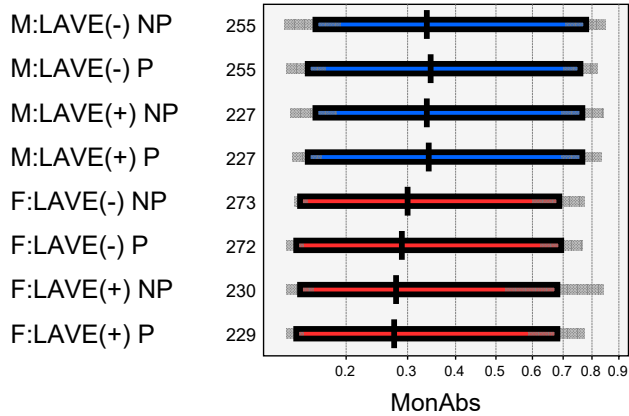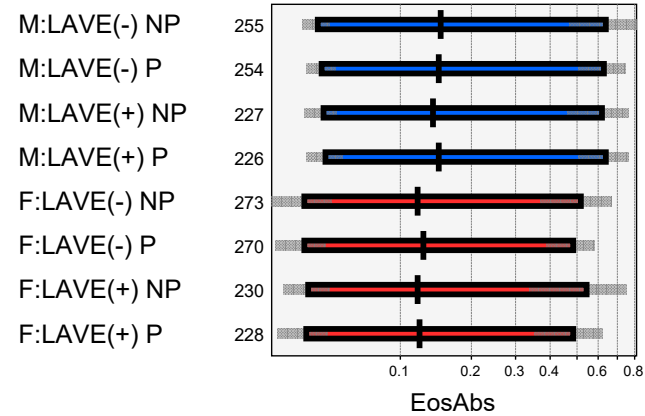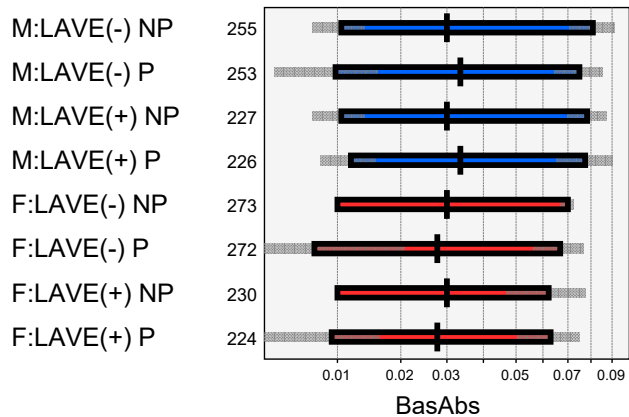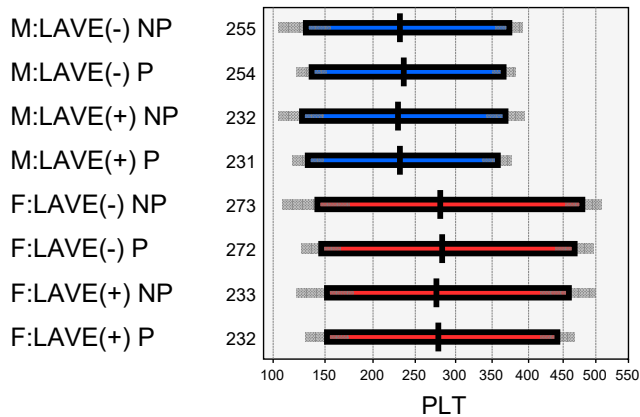

Supplement: S2 Fig — (PDF) [file pone.0198444.s002.pdf]
