# Supplementary material for: Complete blood count reference intervals from a healthy adult urban population in Kenya
Source: PLoS One. 2018 Jun 7;13(6):e0198444. doi: 10.1371/journal.pone.0198444 (PMC5991659; doi:10.1371/journal.pone.0198444)

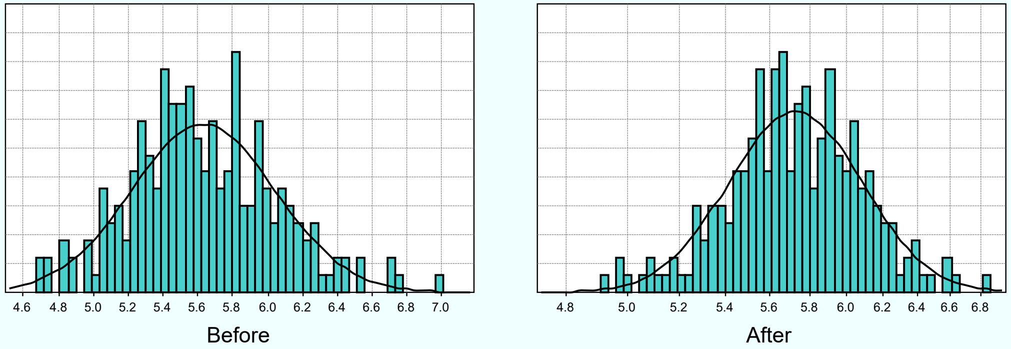

RBC M n=226
Para: 4.94 ~ 5.62 ~ 6.52
Nonpara: 4.90 ~ 5.61 ~ 6.60
Pow=0.614 TPos=4.494
Kurt=2.858 Skew=0.025

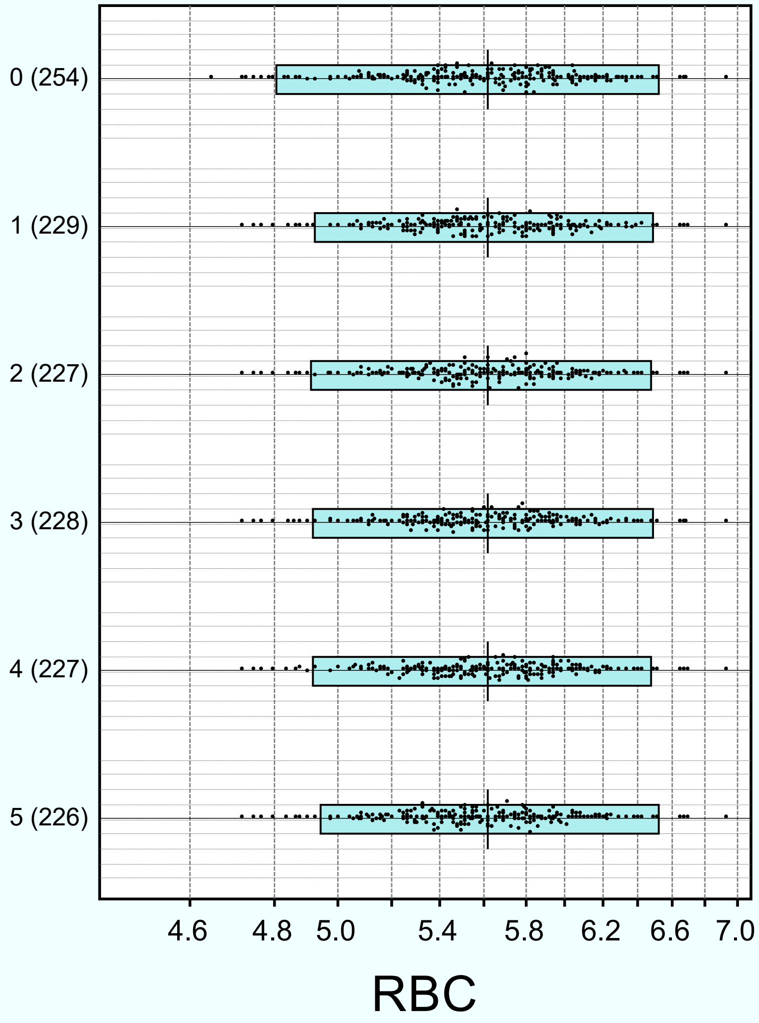

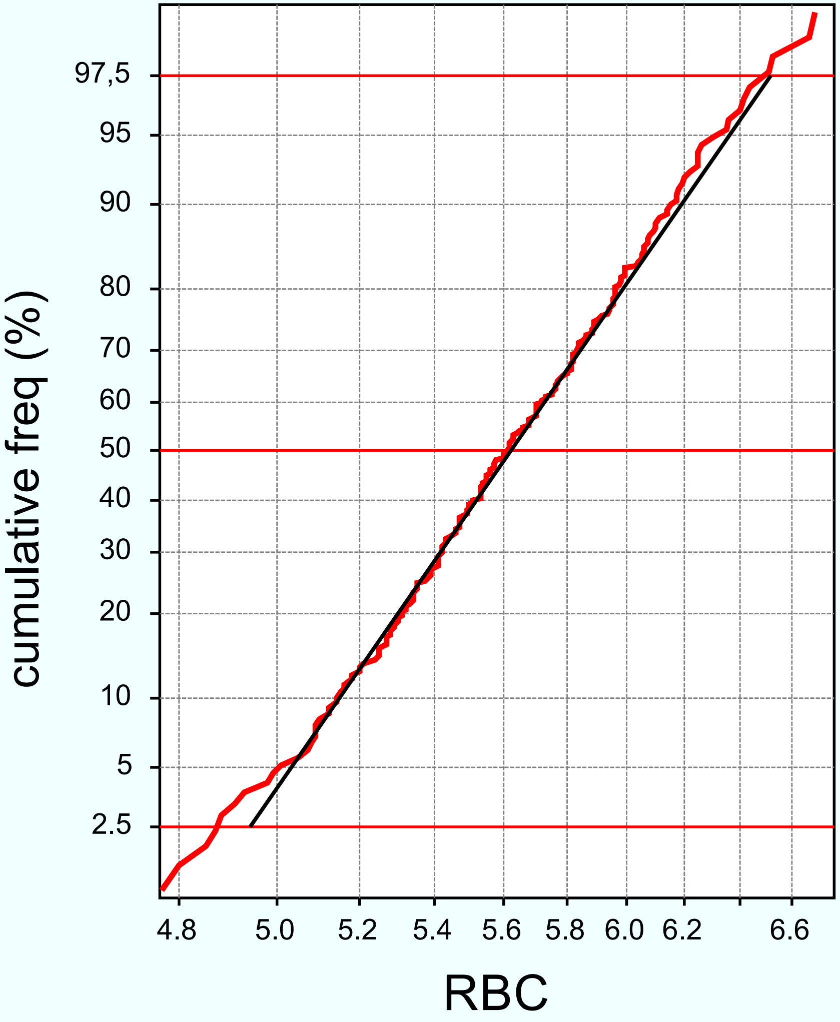

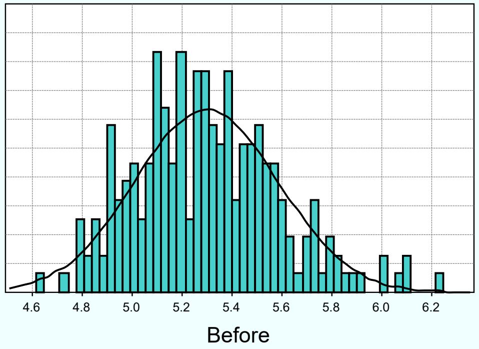

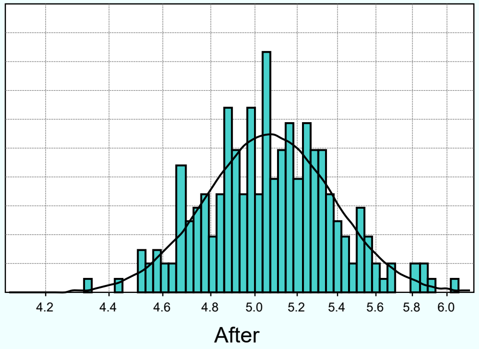

RBC F n=228
Para: 4.31 ~ 4.93 ~ 5.76
Nonpara: 4.33 ~ 4.94 ~ 5.84
Pow=0.656 TPos=3.924
Kurt=2.69 Skew=-0.011

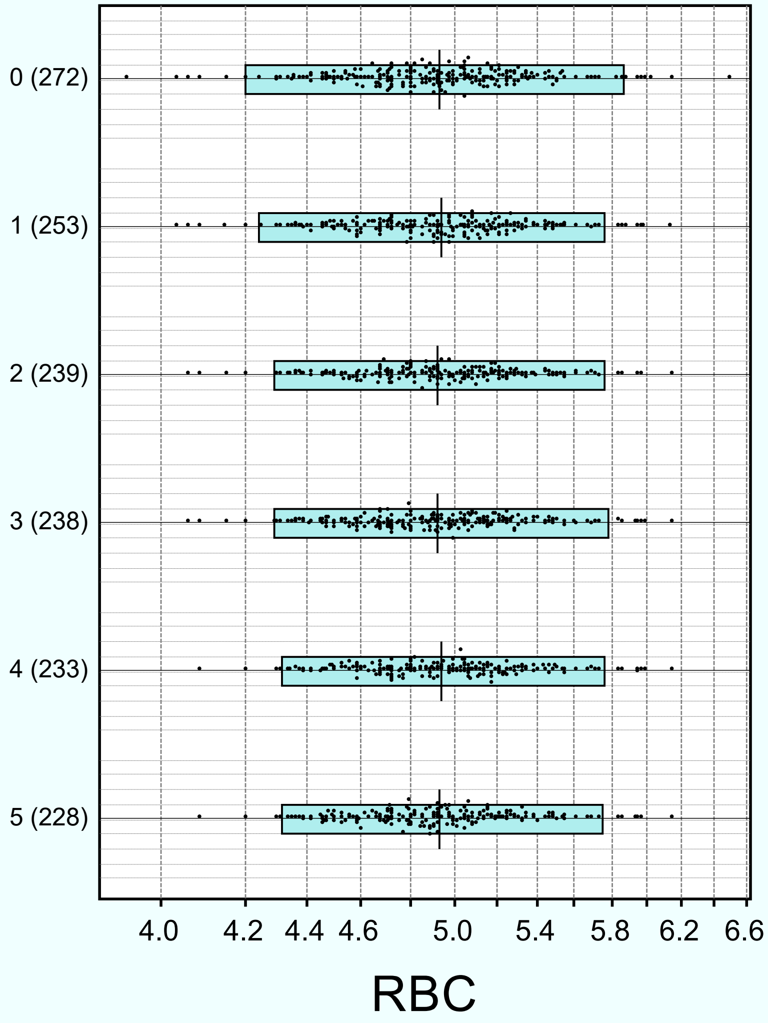

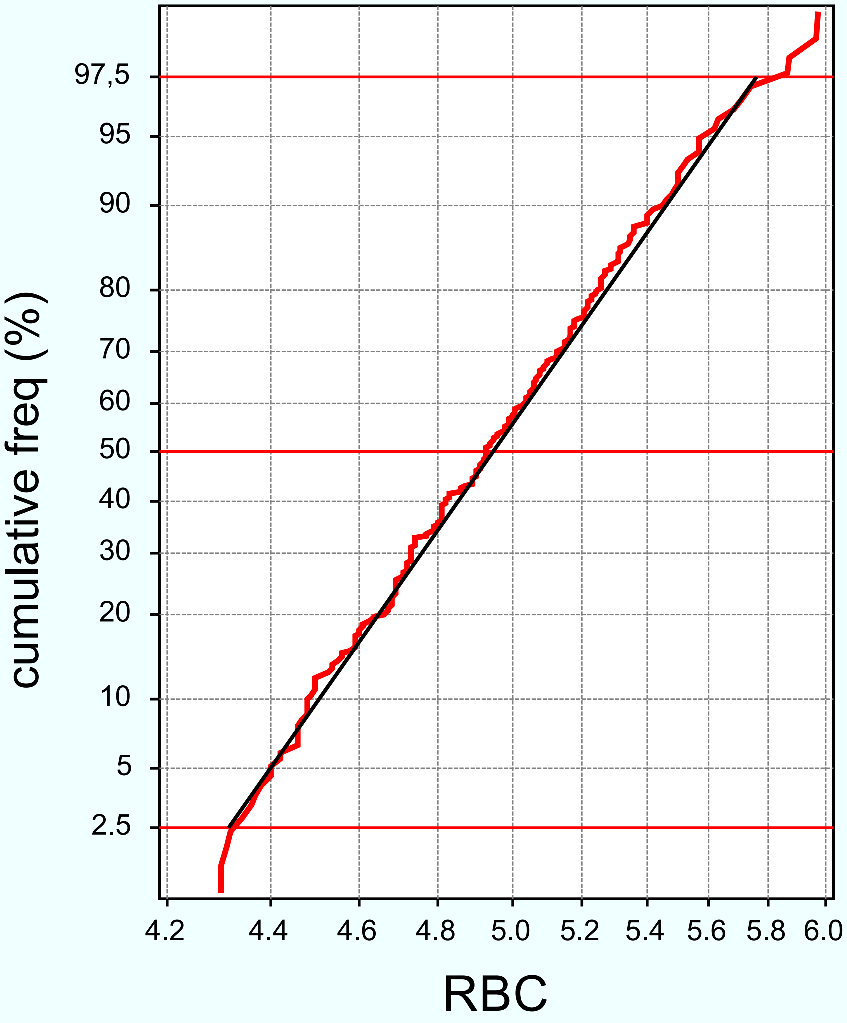

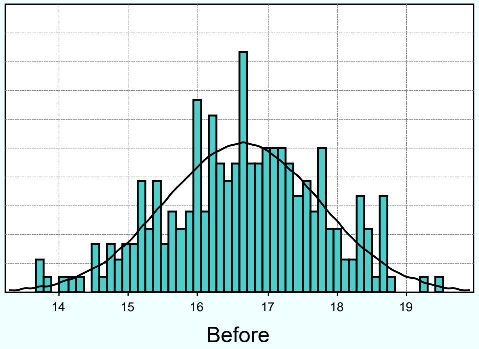

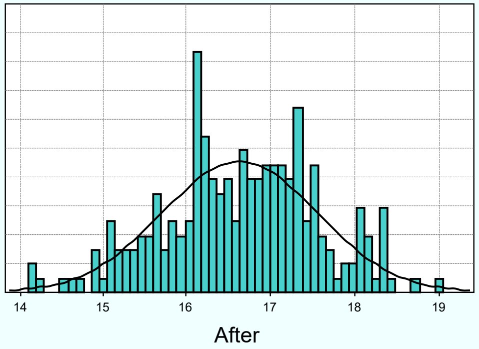

Hb M n=232
Para: 14.54 ~ 16.64 ~ 18.75
Nonpara: 14.43 ~ 16.70 ~ 18.59
Pow=1.03 TPos=12.528
Kurt=2.603 Skew=-0.112

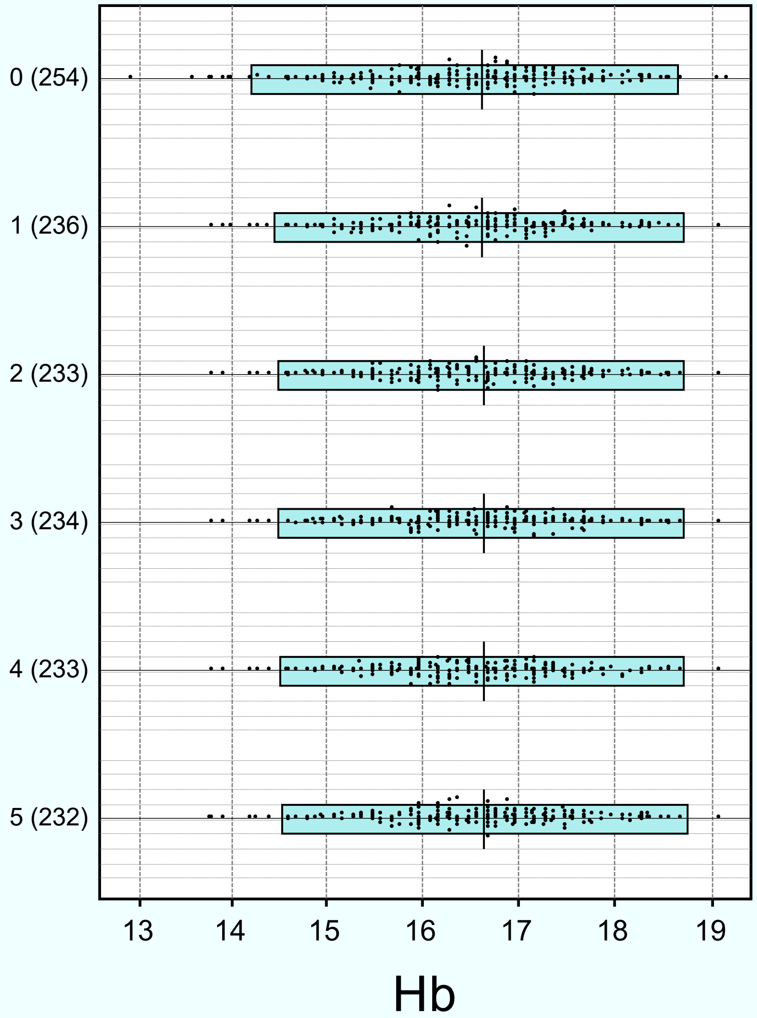

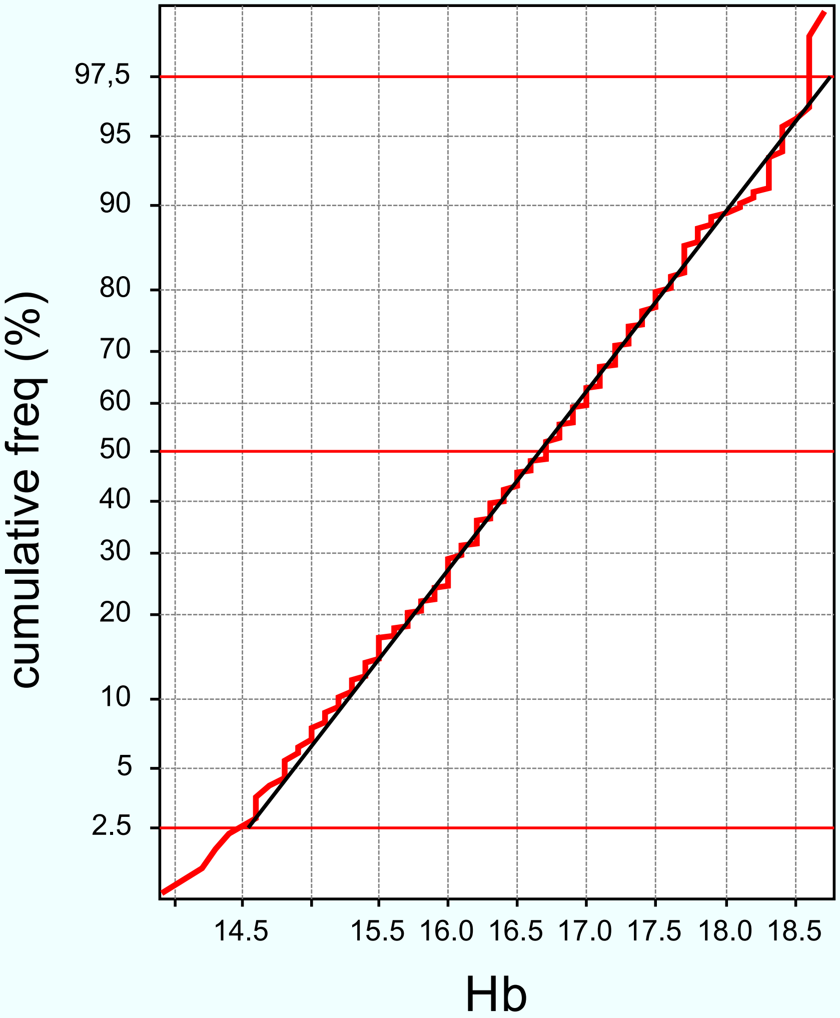

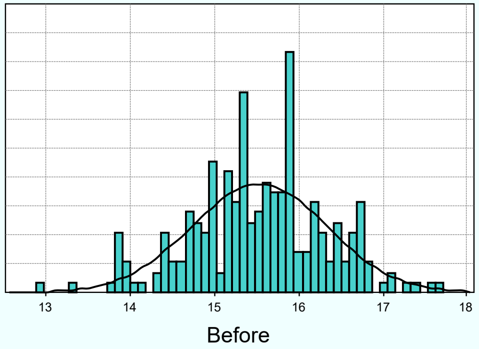

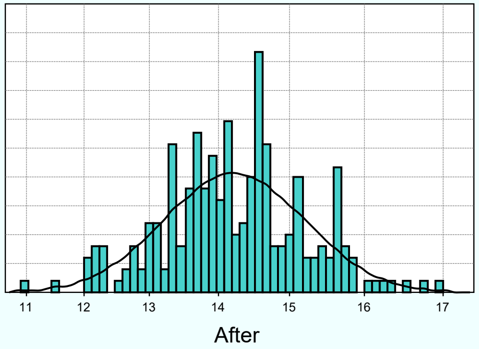

Hb F n=236
Para: 12.01 ~ 14.35 ~ 16.51
Nonpara: 11.97 ~ 14.30 ~ 16.60
Pow=1.189 TPos=9.986
Kurt=2.694 Skew=-0.036

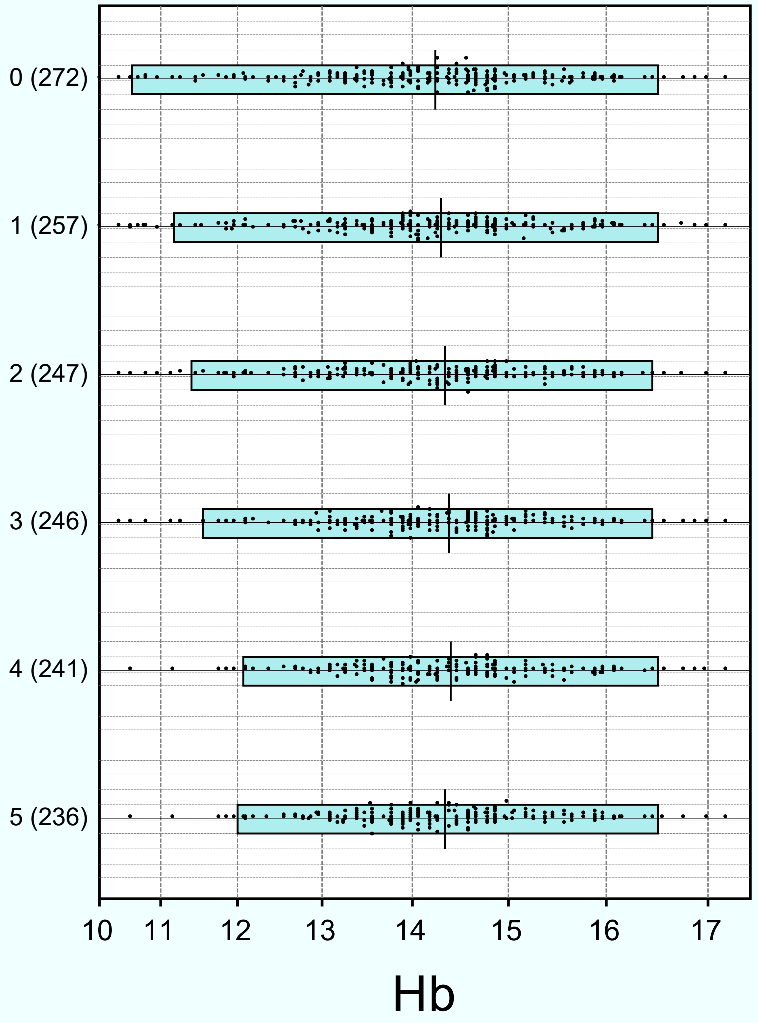

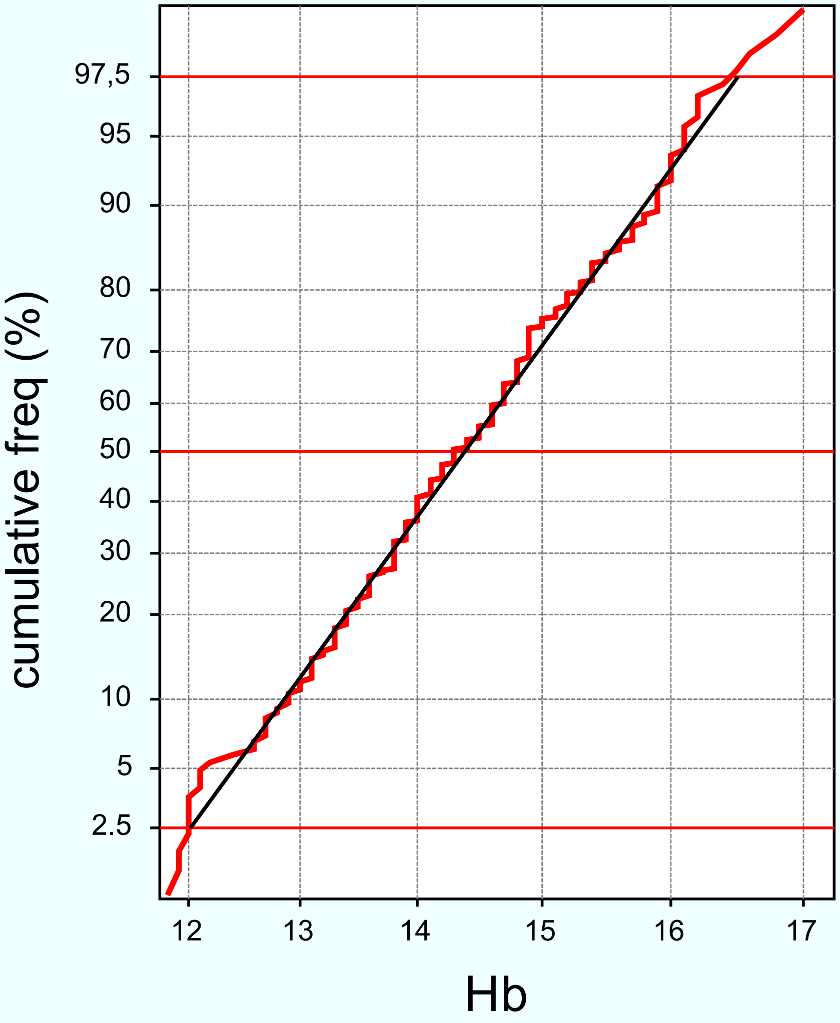

Hct M n=232
Para: 0.431 ~ 0.490 ~ 0.550
Nonpara: 0.433 ~ 0.490 ~ 0.543
Pow=0.98 TPos=0.38
Kurt=2.449 Skew=-0.038

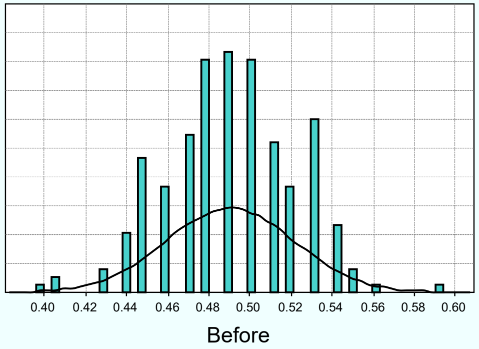

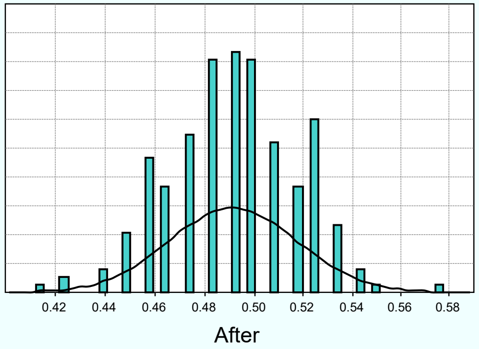

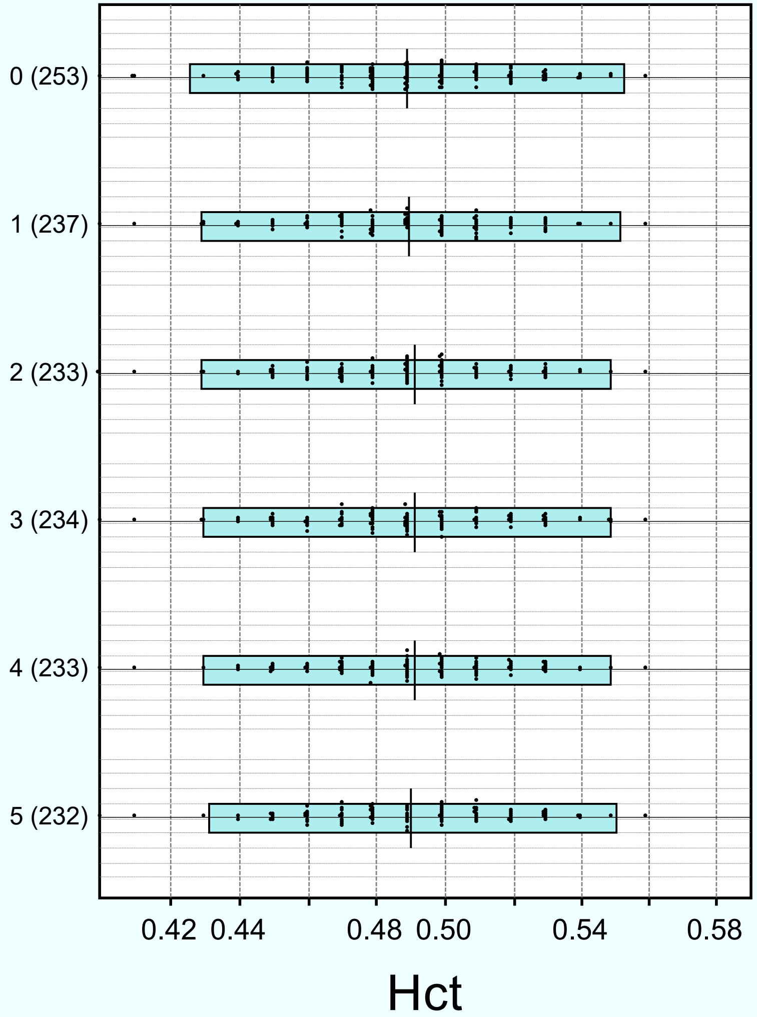

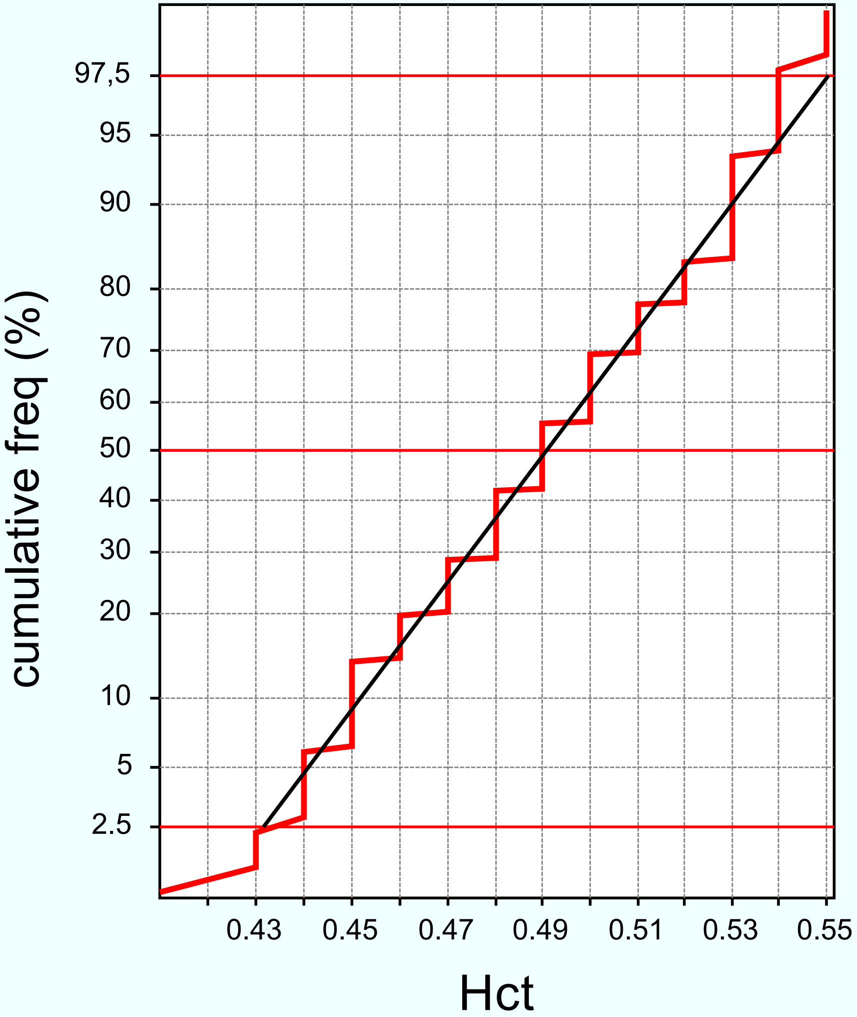

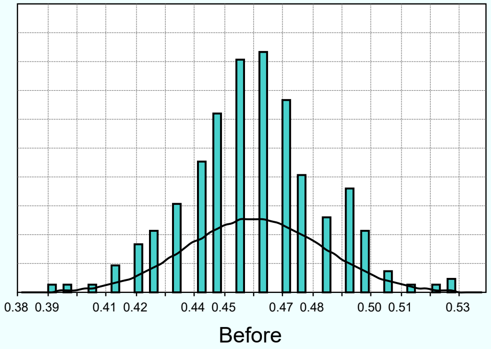

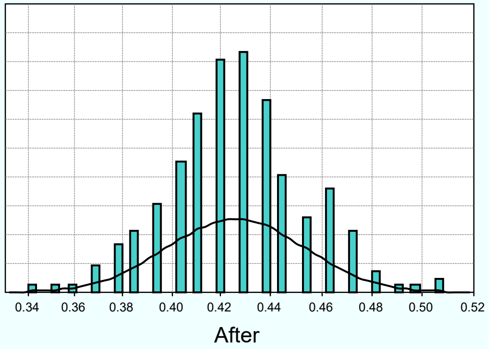

Hct F n=237
Para: 0.364 ~ 0.427 ~ 0.490
Nonpara: 0.363 ~ 0.430 ~ 0.488
Pow=1.061 TPos=0.297
Kurt=2.704 Skew=-0.11

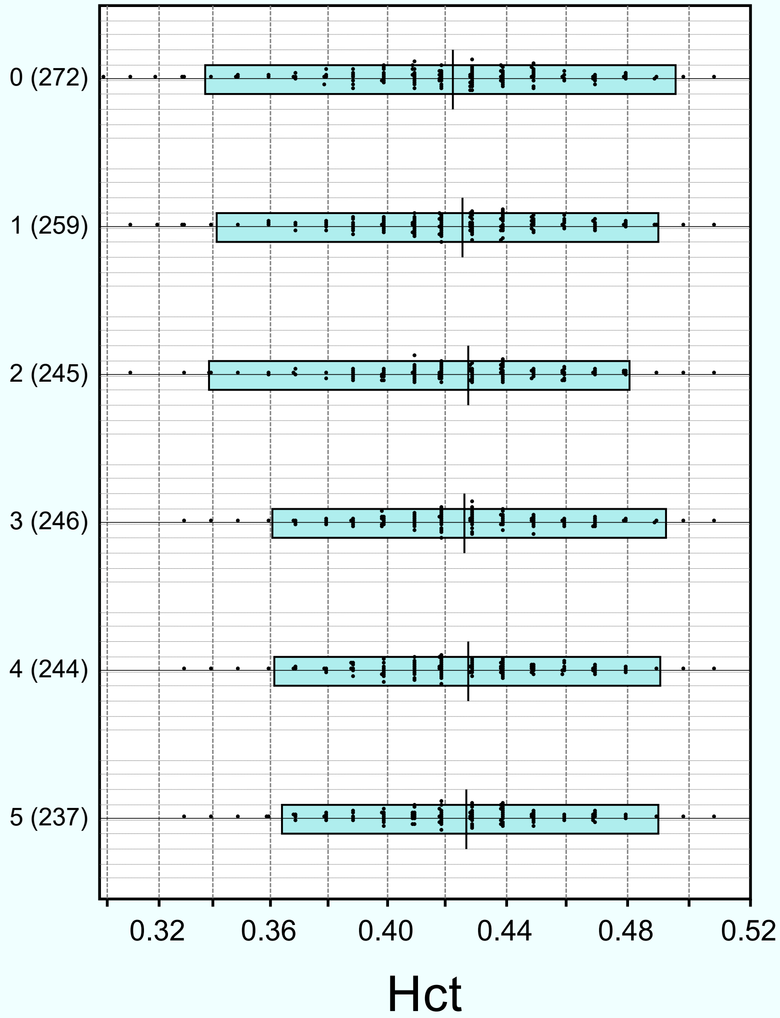

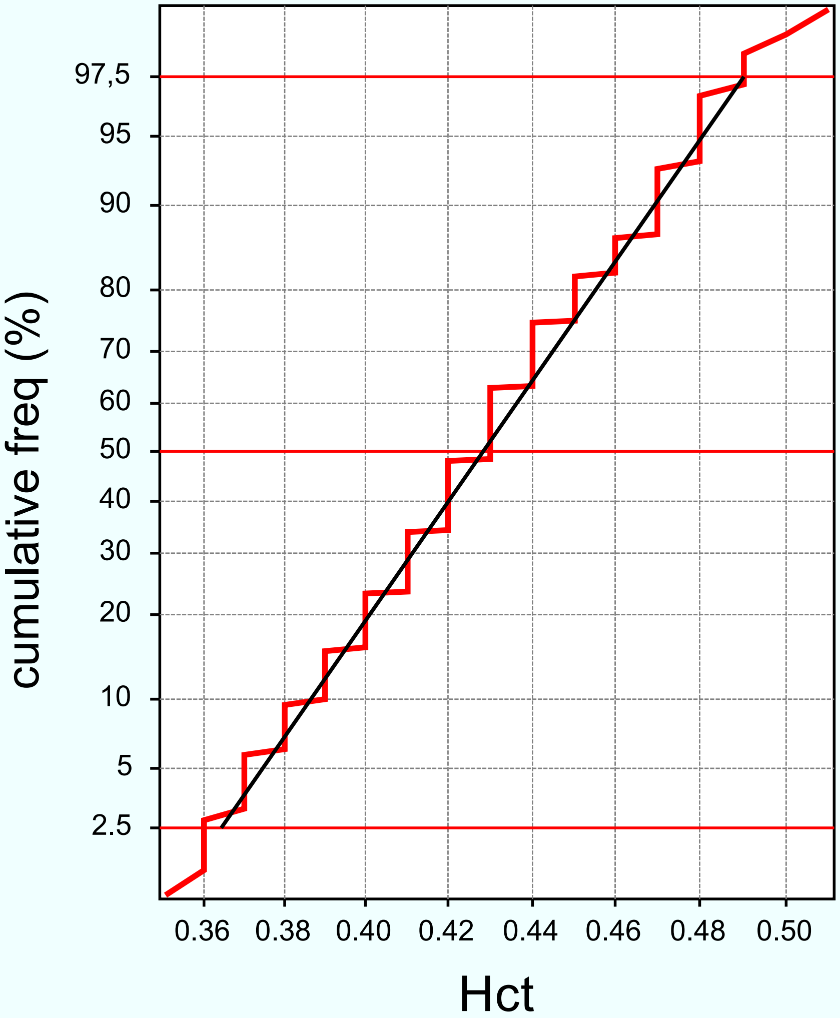

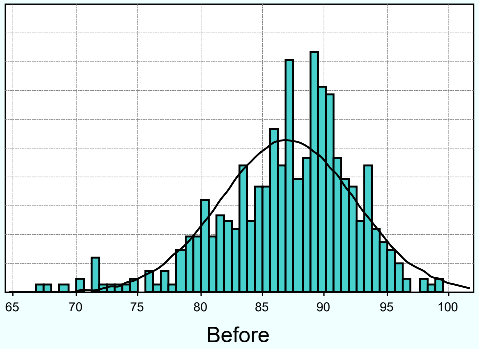

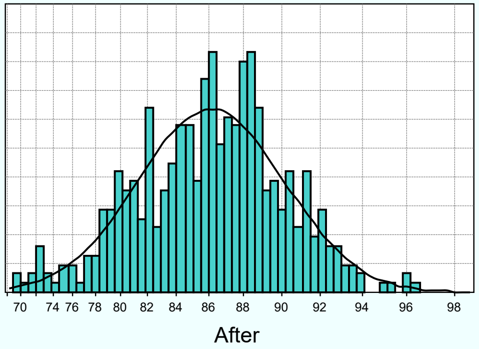

MCV MF n=466
Para: 75.7 ~ 87.4 ~ 95.6
Nonpara: 73.8 ~ 87.6 ~ 95.2
Pow=1.963 TPos=59.744
Kurt=2.753 Skew=-0.221

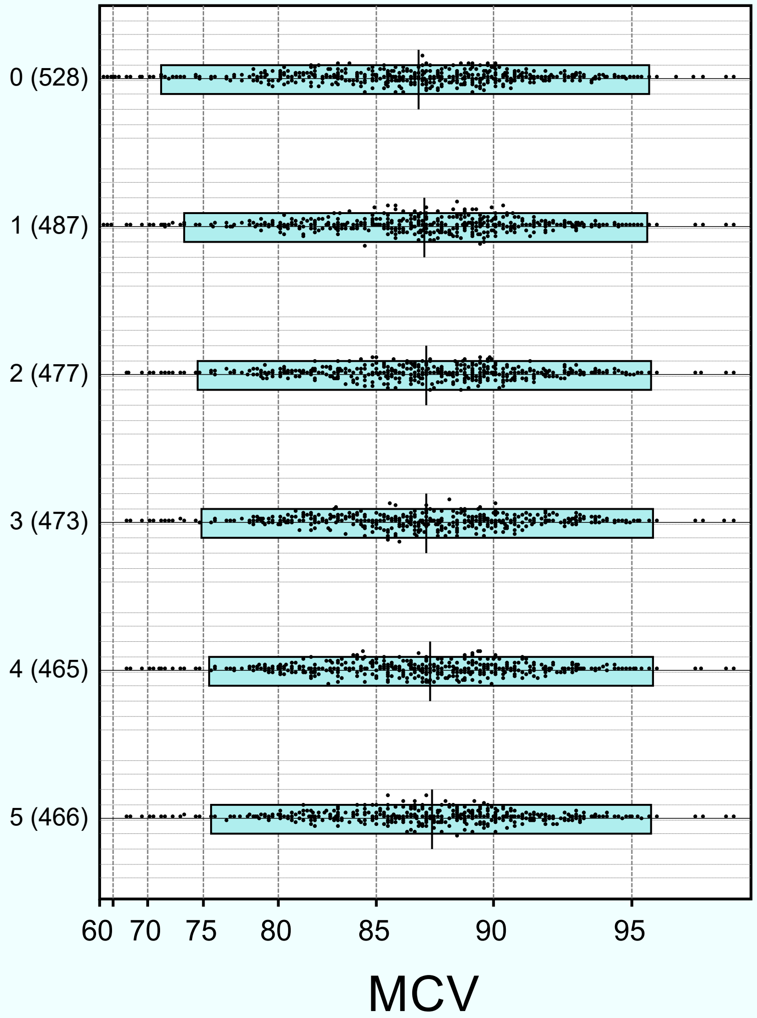

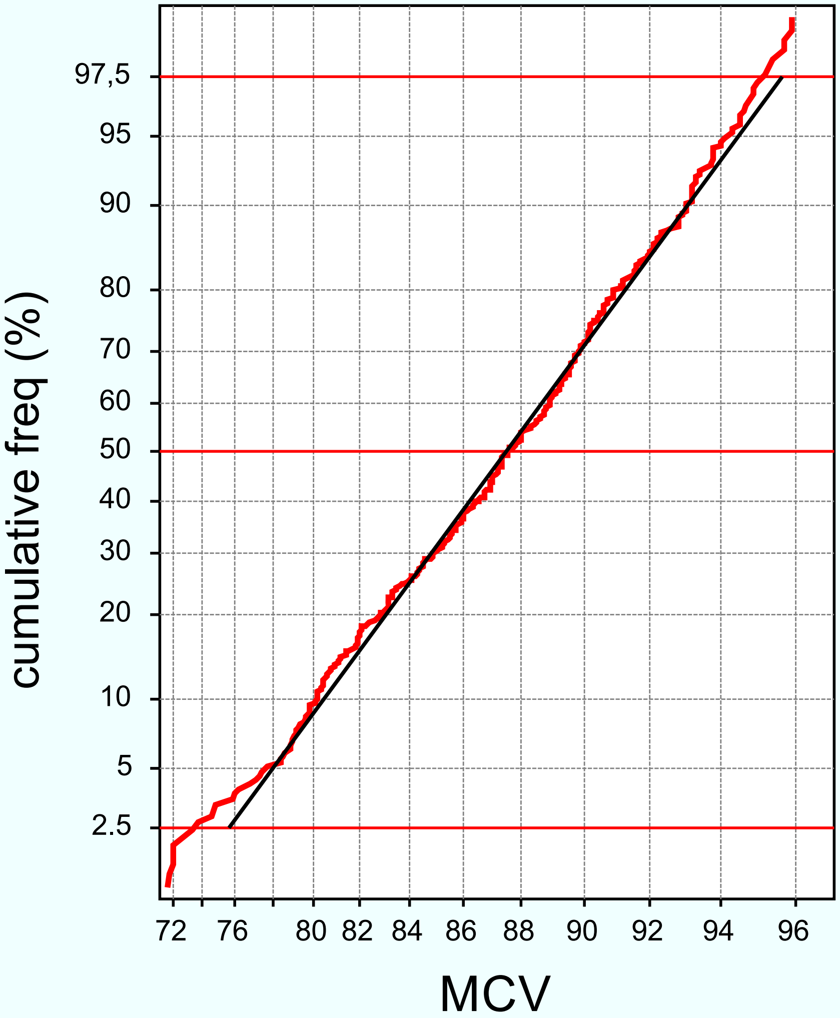

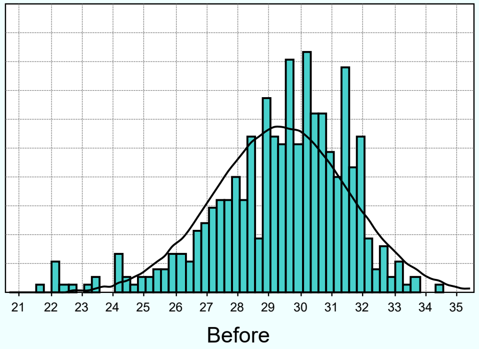

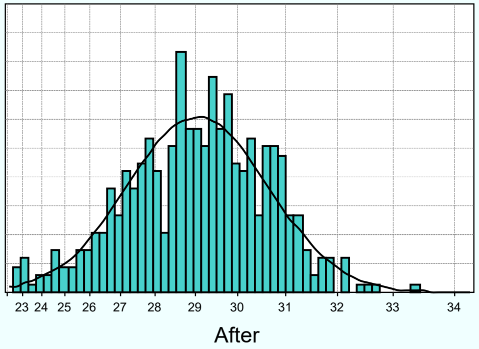

MCH MF n=462
Para: 24.79 ~ 29.58 ~ 32.75
Nonpara: 24.26 ~ 29.70 ~ 32.62
Pow=2.169 TPos=17.76
Kurt=2.706 Skew=-0.203

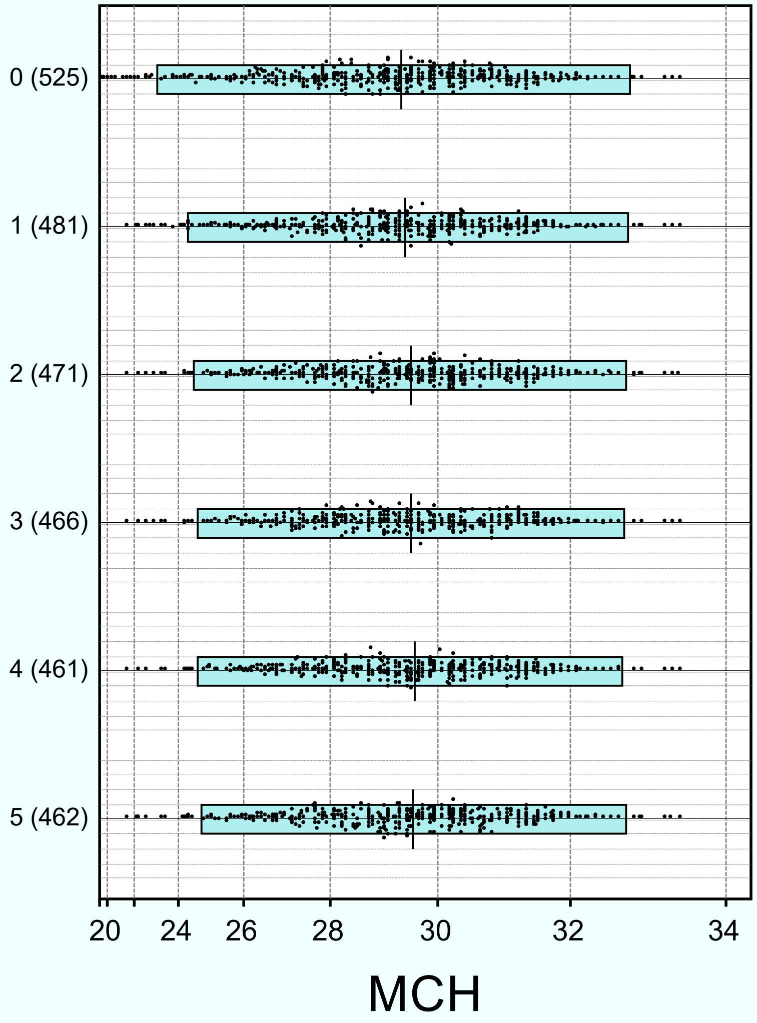

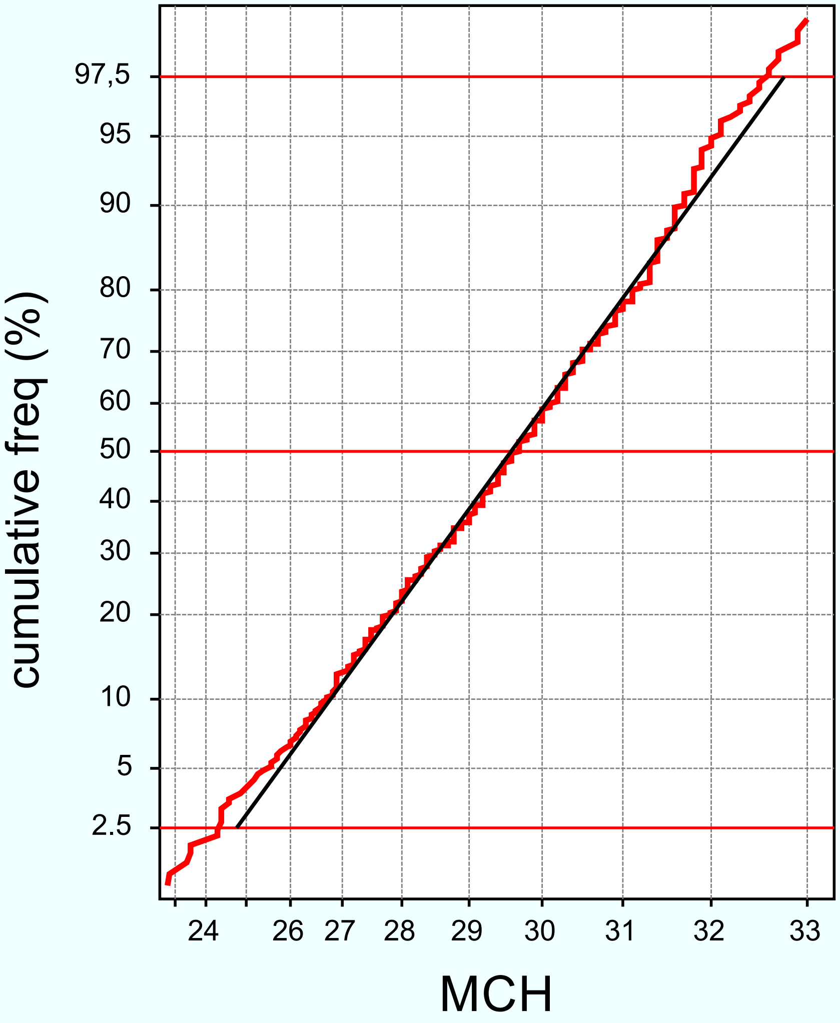

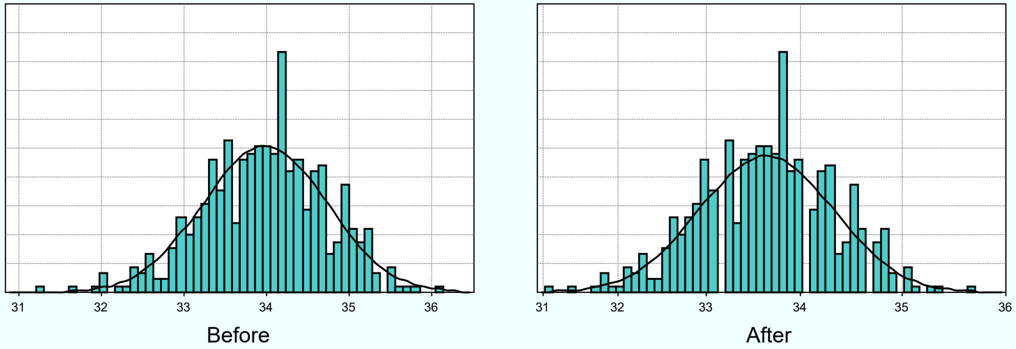

MCHC MF n=462
Para: 32.20 ~ 33.79 ~ 35.24
Nonpara: 32.14 ~ 33.80 ~ 35.19
Pow=1.186 TPos=30.629
Kurt=2.709 Skew=-0.092

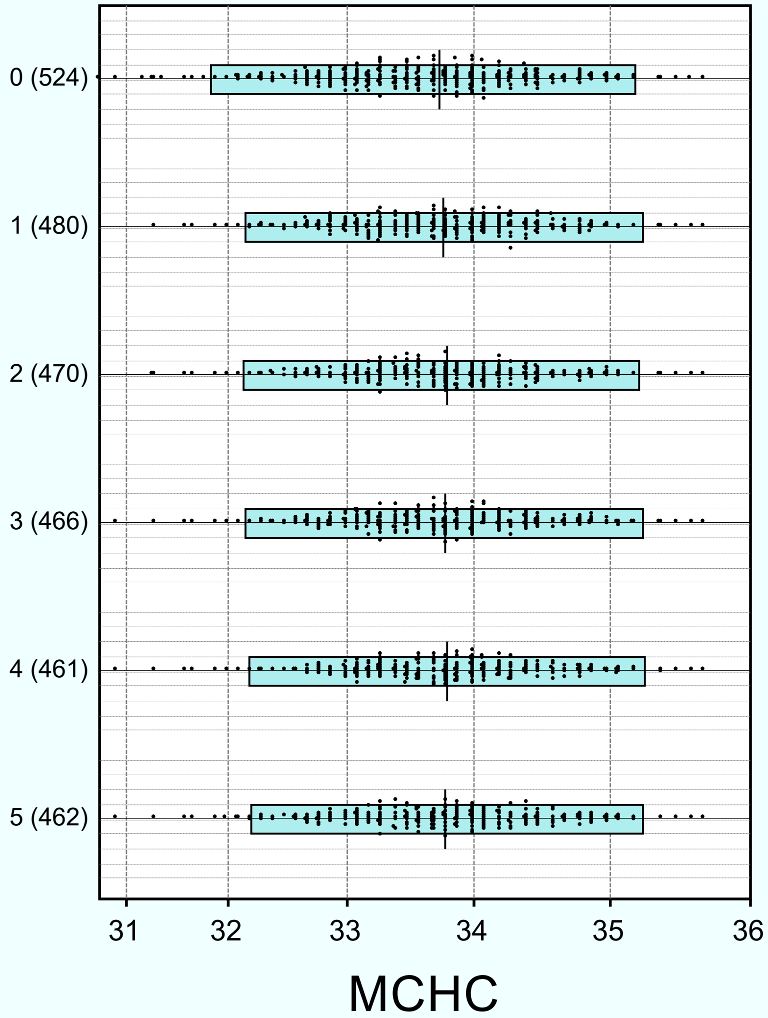

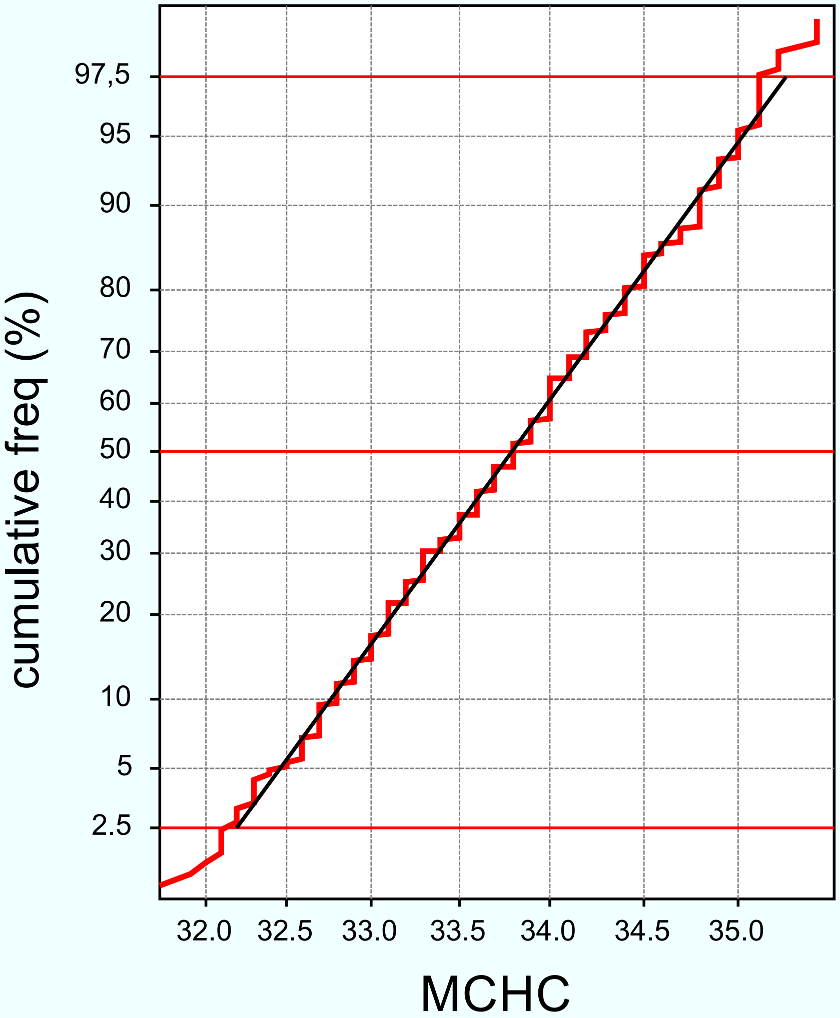

RDW MF n=459
Para: 11.29 ~ 12.97 ~ 15.22
Nonpara: 11.22 ~ 12.90 ~ 15.68
Pow=0.649 TPos=10.41
Kurt=2.955 Skew=0.188

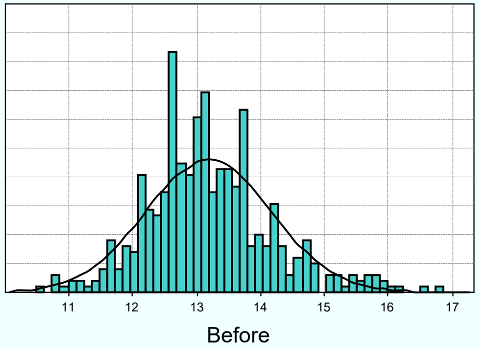

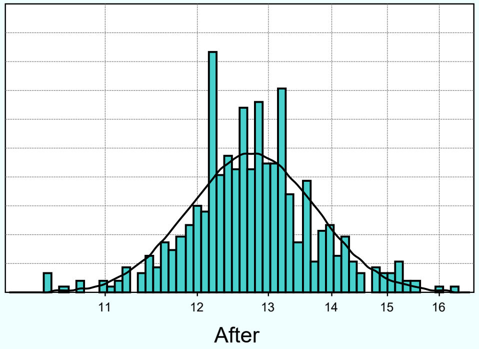

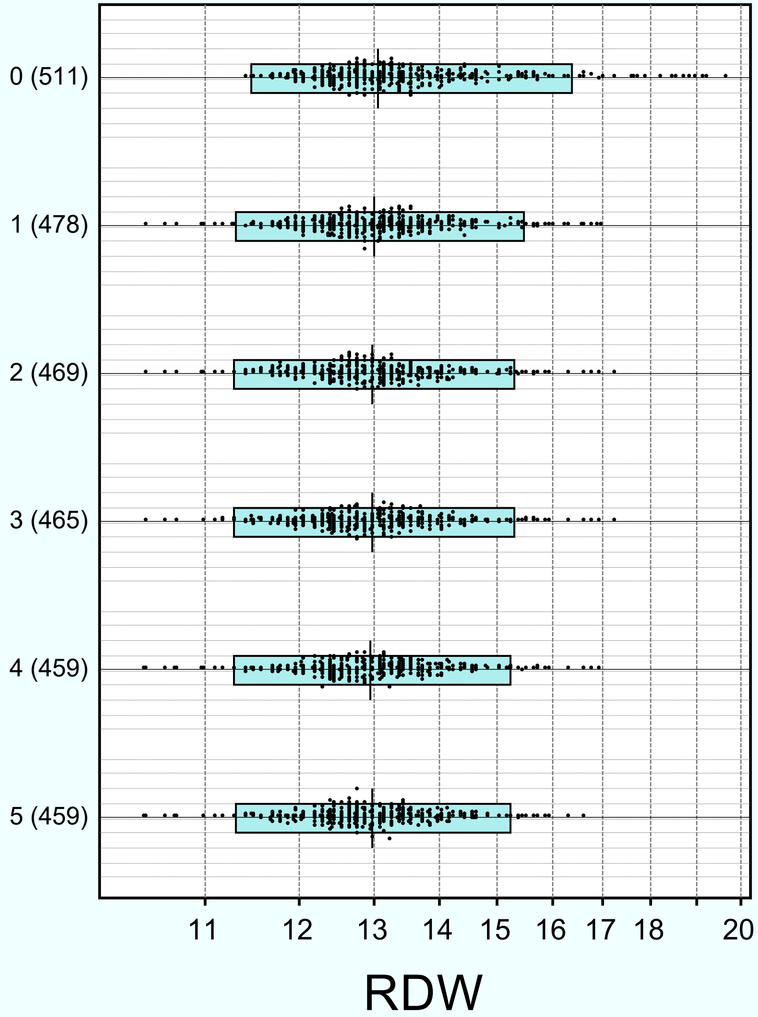

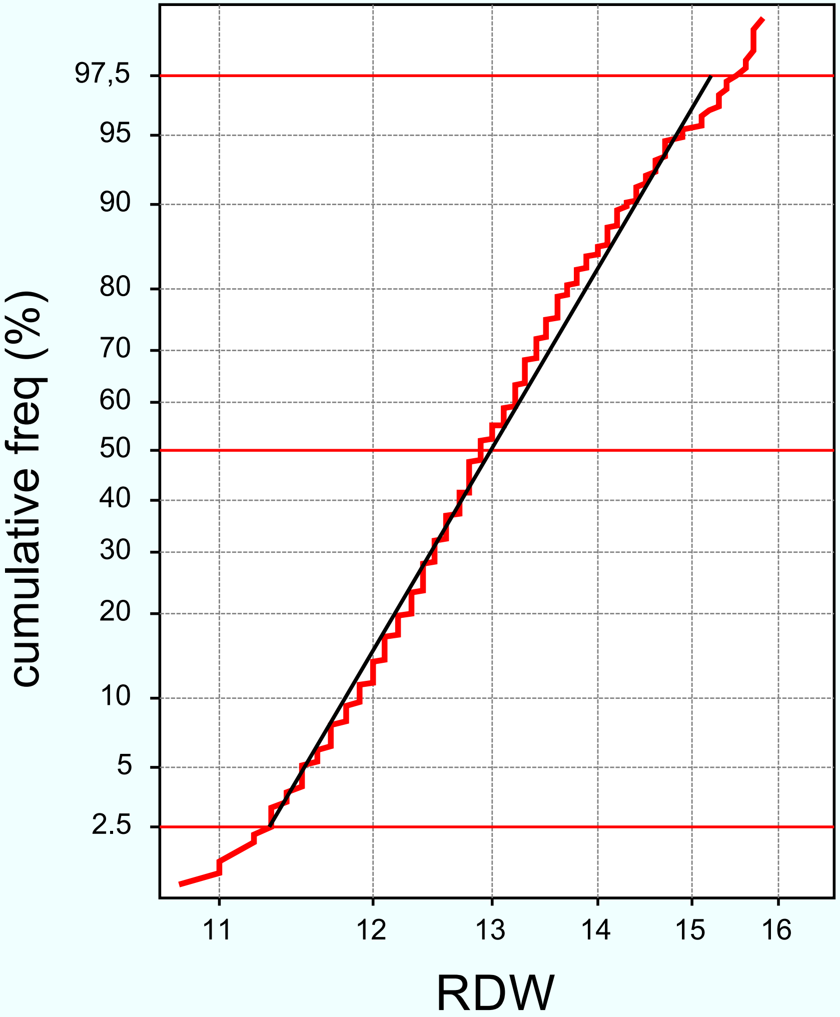

WBC MF n=465
Para: 3.08 ~ 4.92 ~ 7.83
Nonpara: 3.09 ~ 4.94 ~ 8.18
Pow=0.493 TPos=2.166
Kurt=2.739 Skew=-0.01

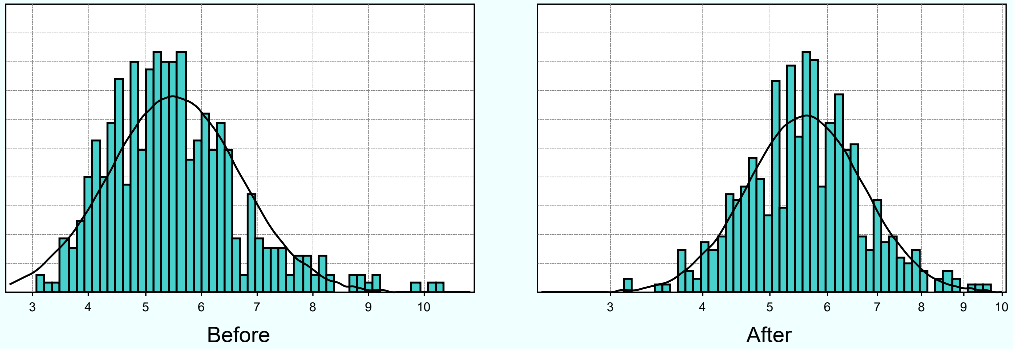

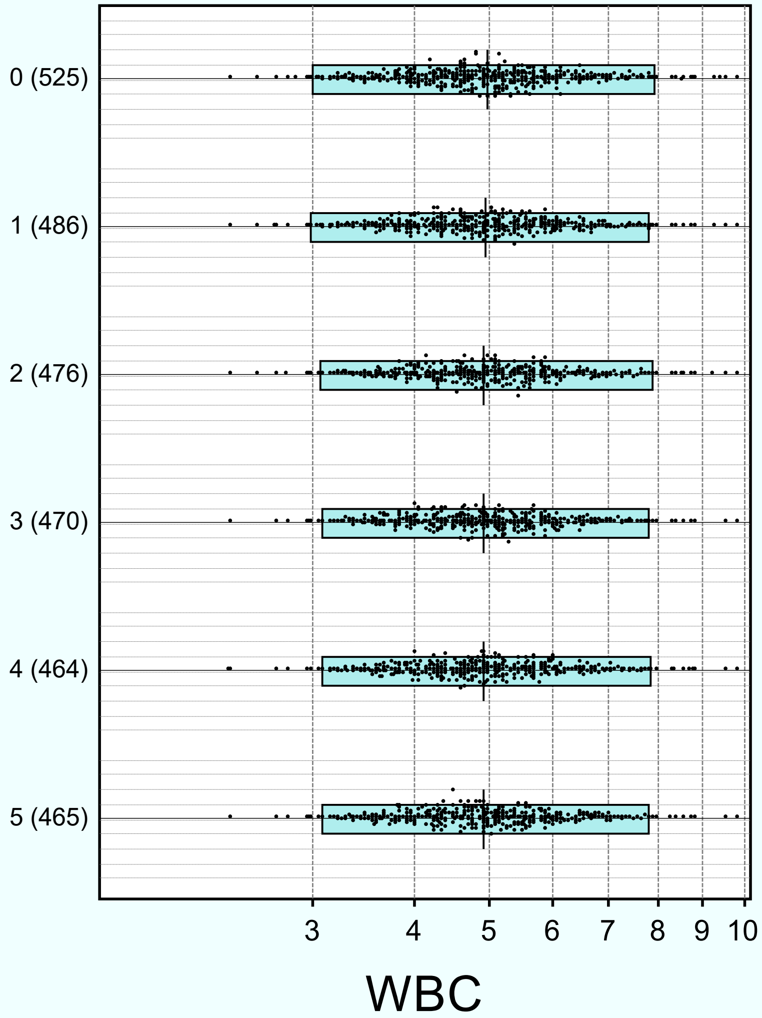

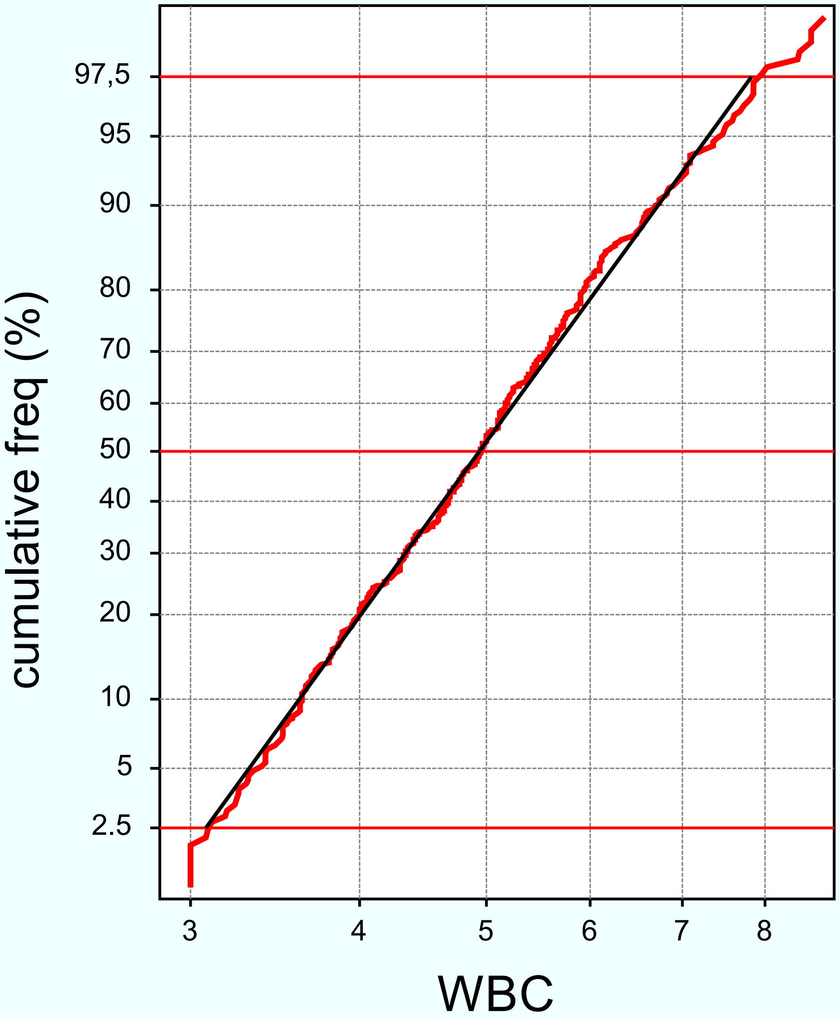

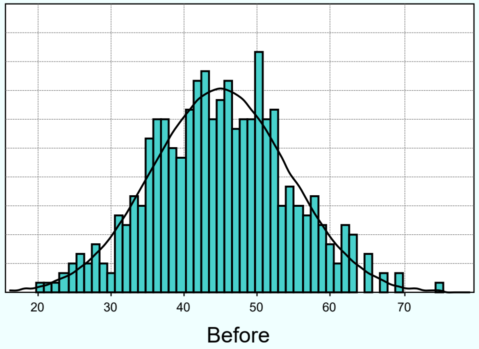

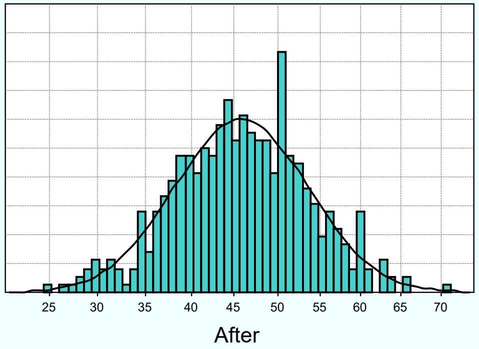

Neu MF n=463
Para: 27.96 ~ 44.71 ~ 63.32
Nonpara: 26.78 ~ 44.90 ~ 62.79
Pow=0.844 TPos=14.227
Kurt=2.769 Skew=-0.11

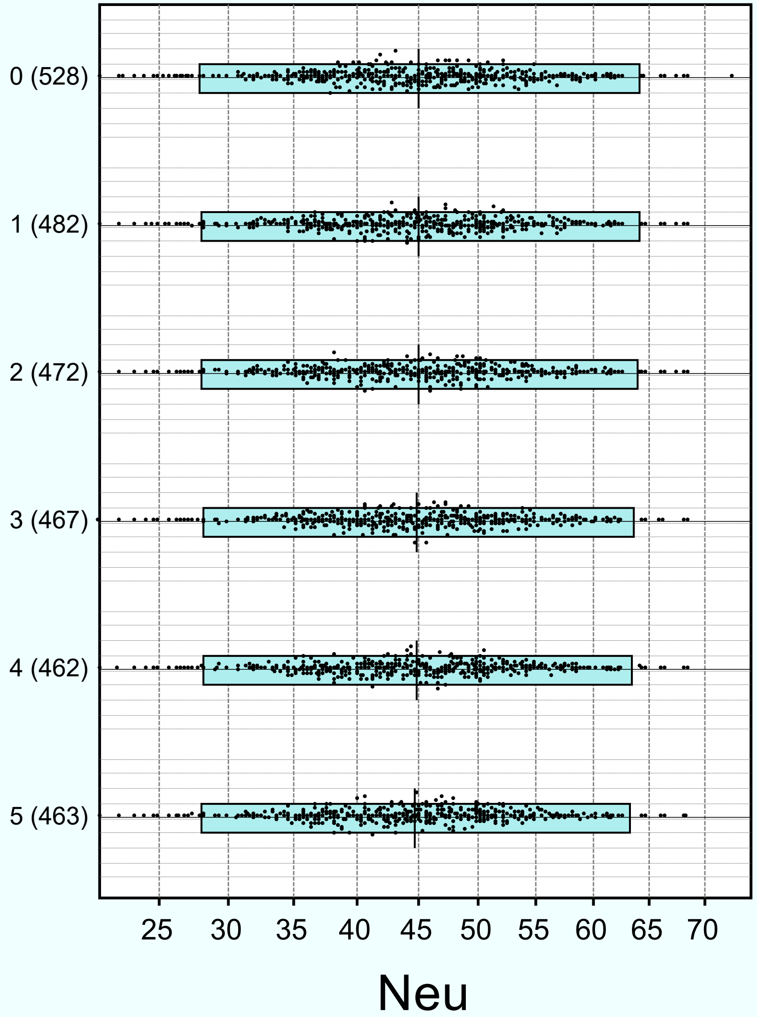

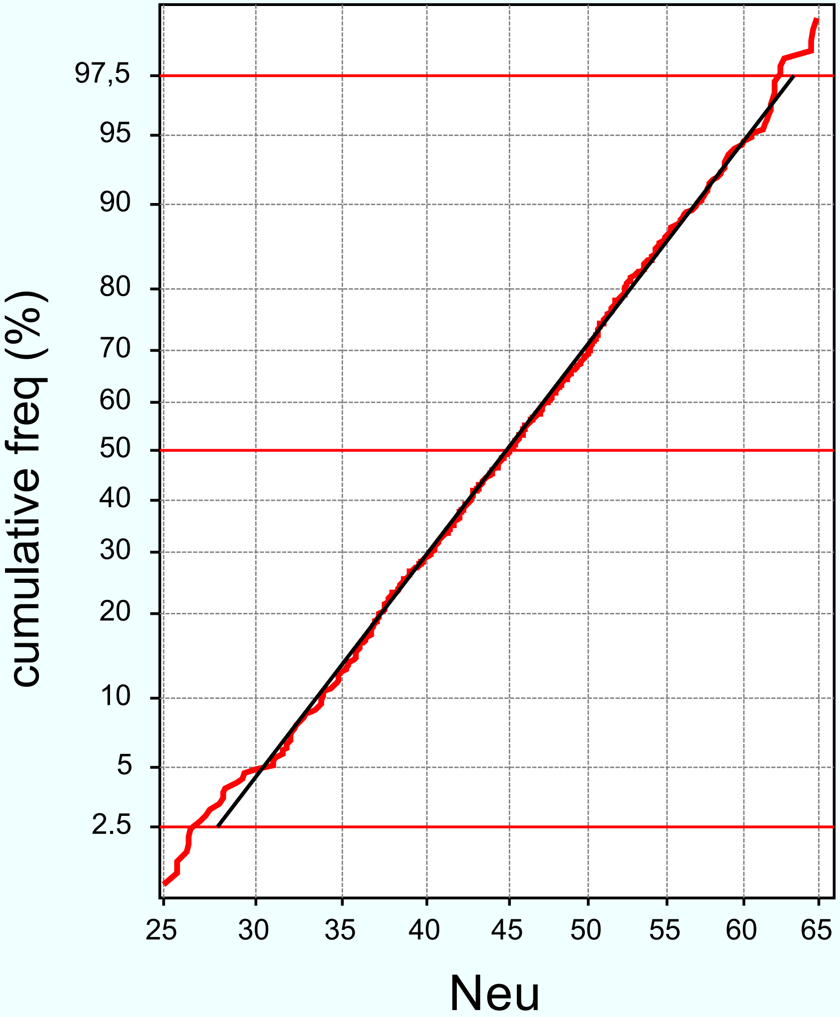

Lym MF n=463
Para: 27.20 ~ 44.16 ~ 60.04
Nonpara: 26.81 ~ 44.20 ~ 60.82
Pow=1.139 TPos=11.138
Kurt=2.806 Skew=-0.008

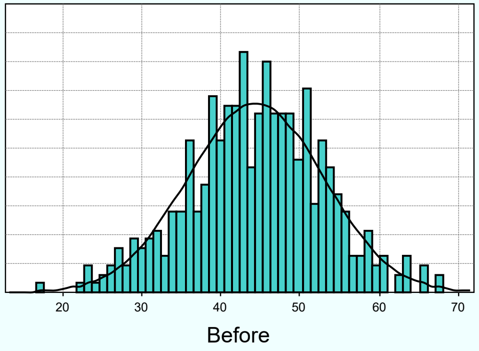

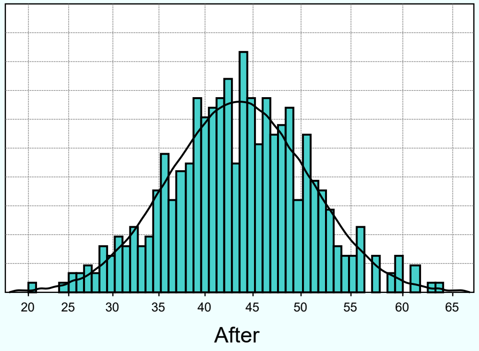

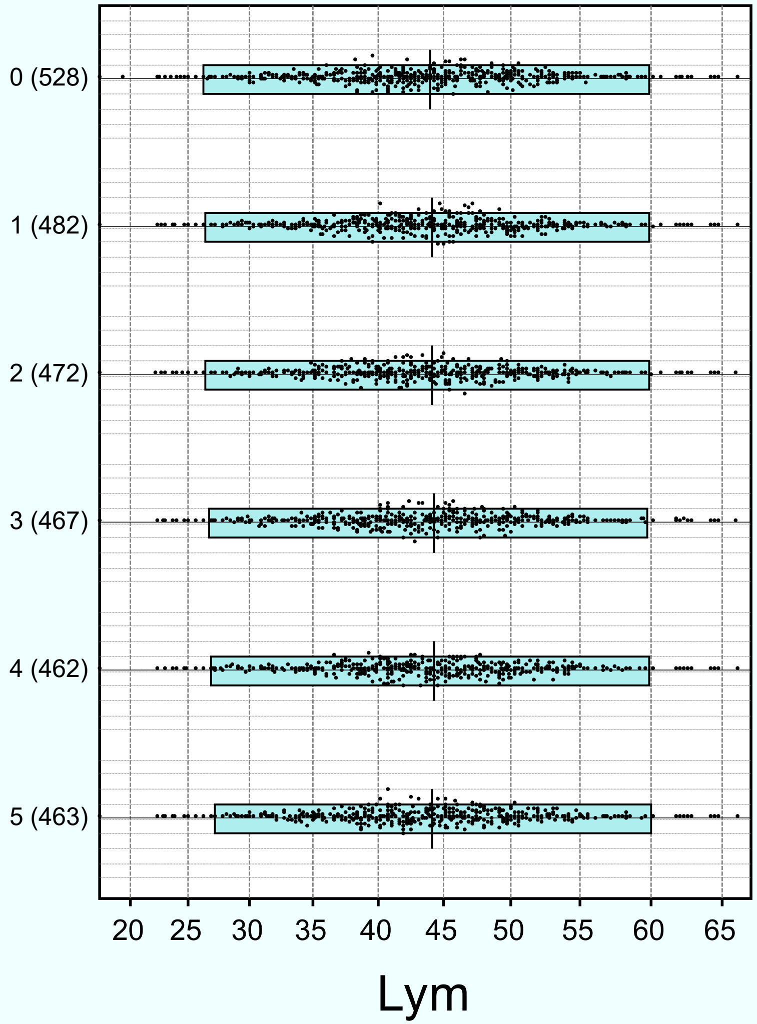

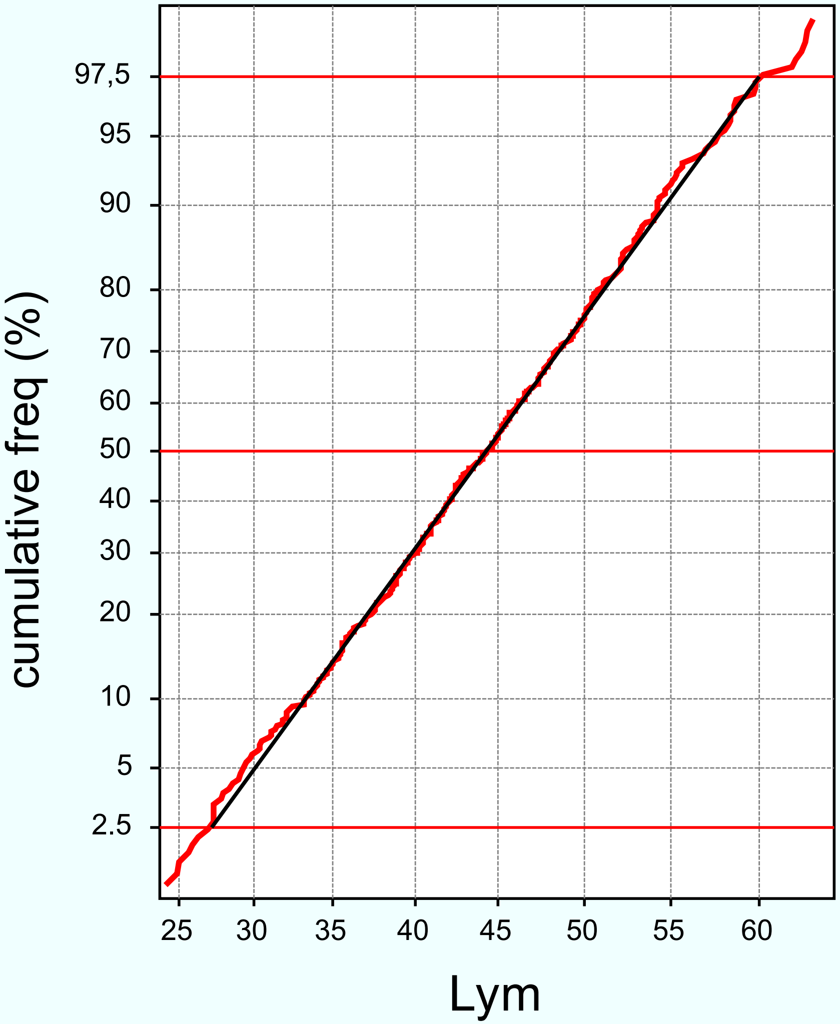

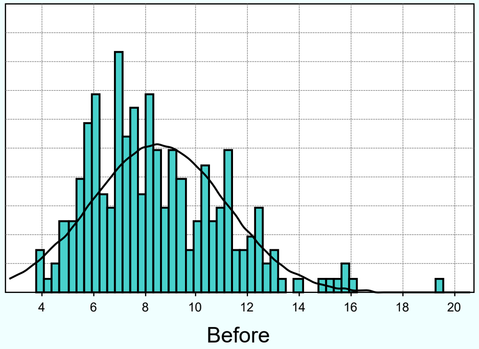

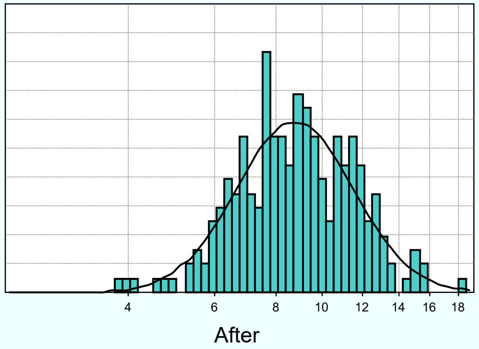

Mon M n=227
Para: 3.46 ~ 7.00 ~ 14.29
Nonpara: 3.29 ~ 7.10 ~ 14.00
Pow=0.354 TPos=2.253
Kurt=2.733 Skew=-0.048

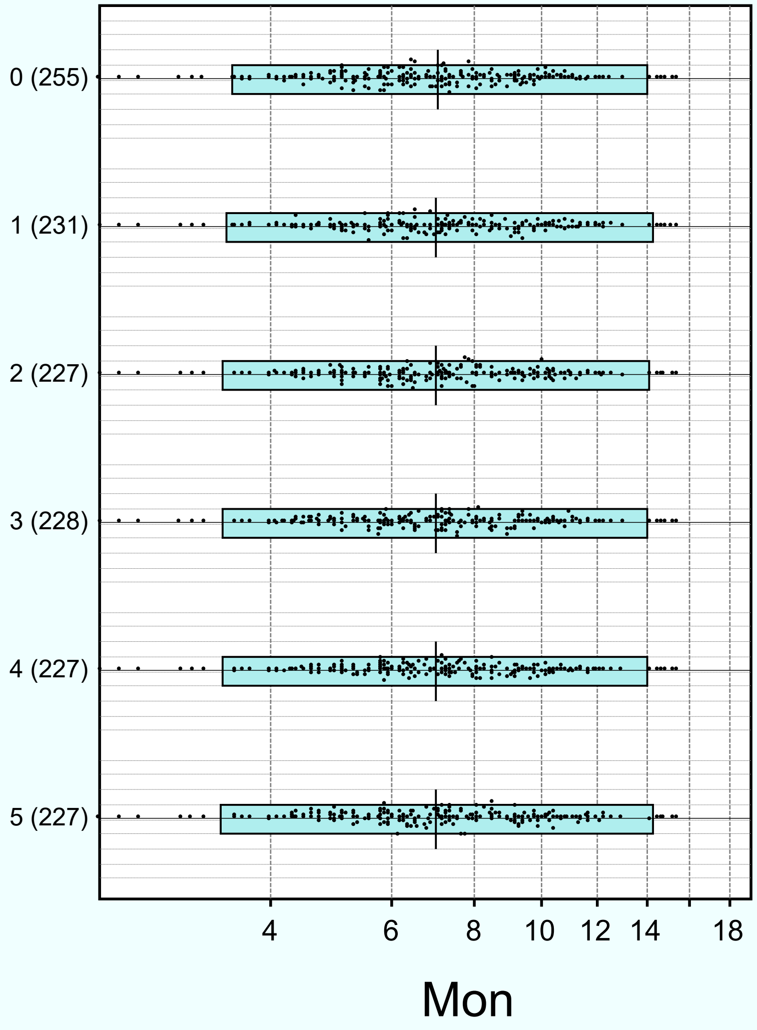

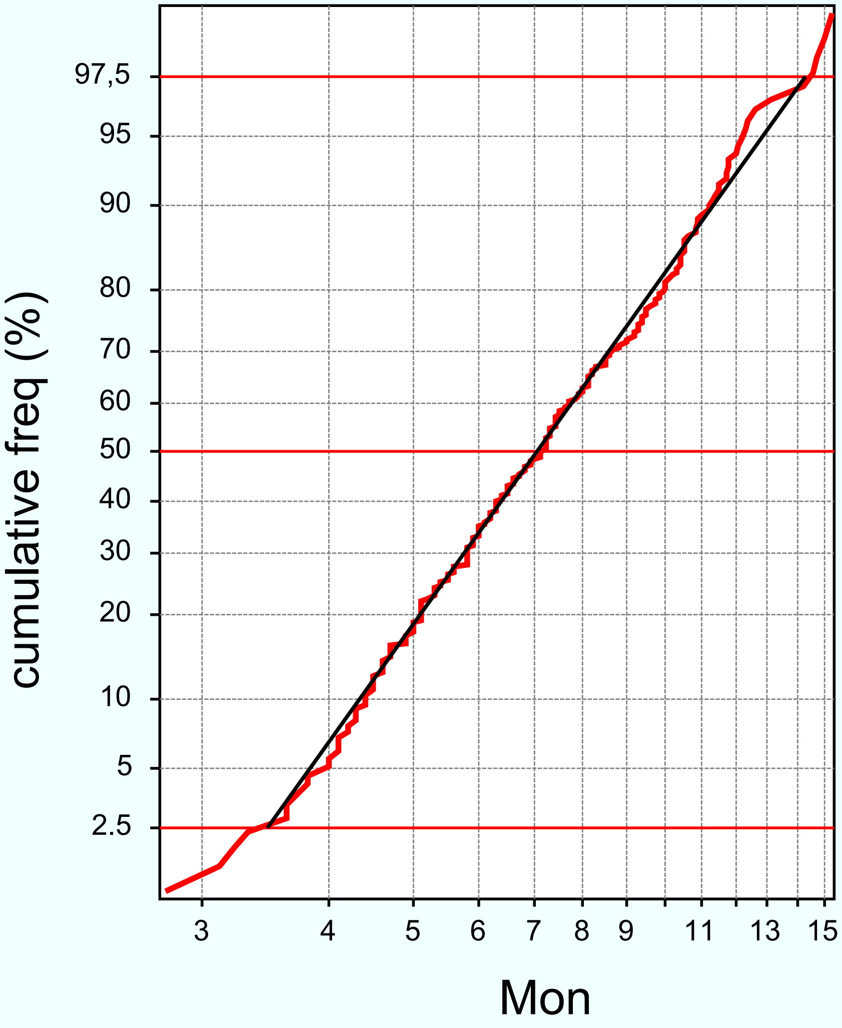

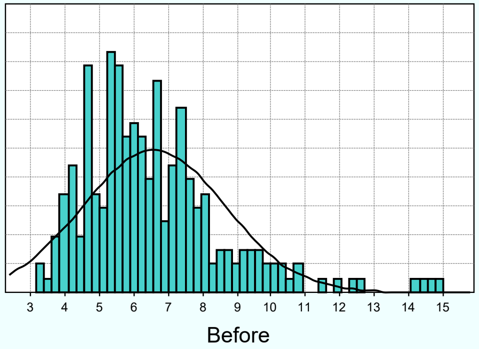

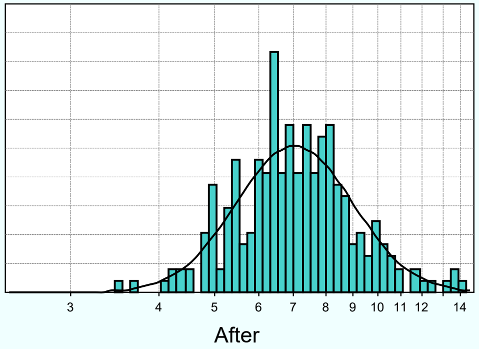

Mon F n=230
Para: 3.22 ~ 5.71 ~ 11.03
Nonpara: 3.24 ~ 5.70 ~ 11.82
Pow=0.342 TPos=2.362
Kurt=2.747 Skew=0.014

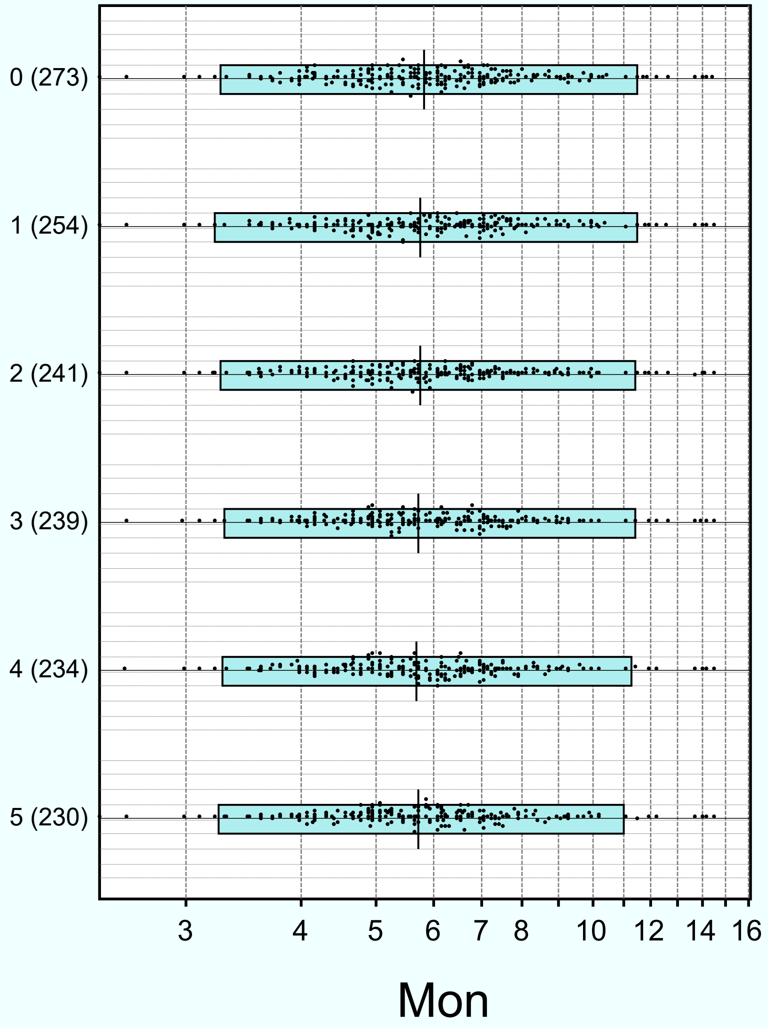

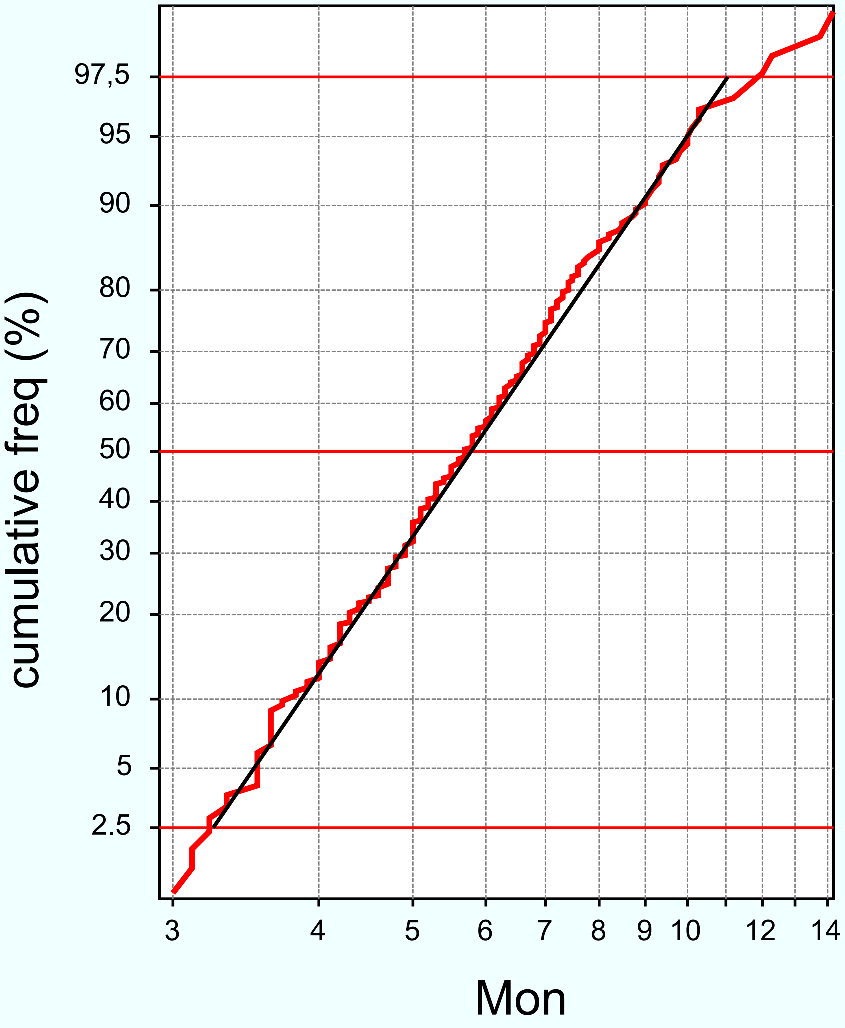

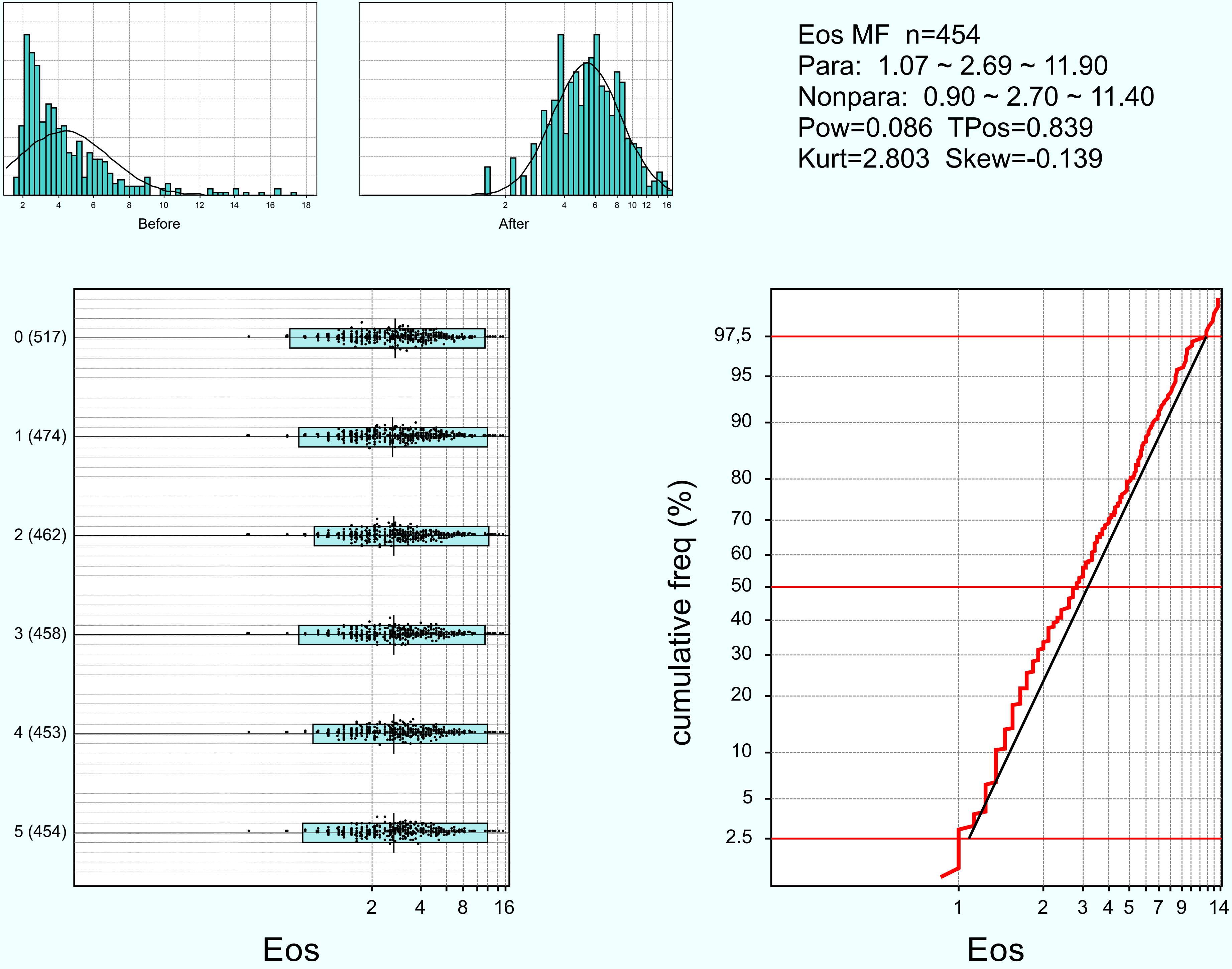

Eos MF n=454
Para: 1.07 ~ 2.69 ~ 11.90
Nonpara: 0.90 ~ 2.70 ~ 11.40
Pow=0.086 TPos=0.839
Kurt=2.803 Skew=-0.139

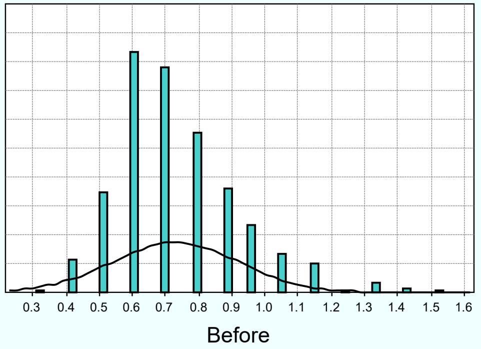

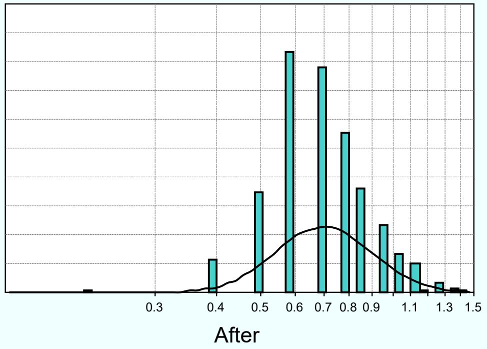

Bas MF n=457
Para: 0.337 ~ 0.613 ~ 1.141
Nonpara: 0.304 ~ 0.600 ~ 1.198
Pow=0.297 TPos=0.19
Kurt=2.721 Skew=0.132

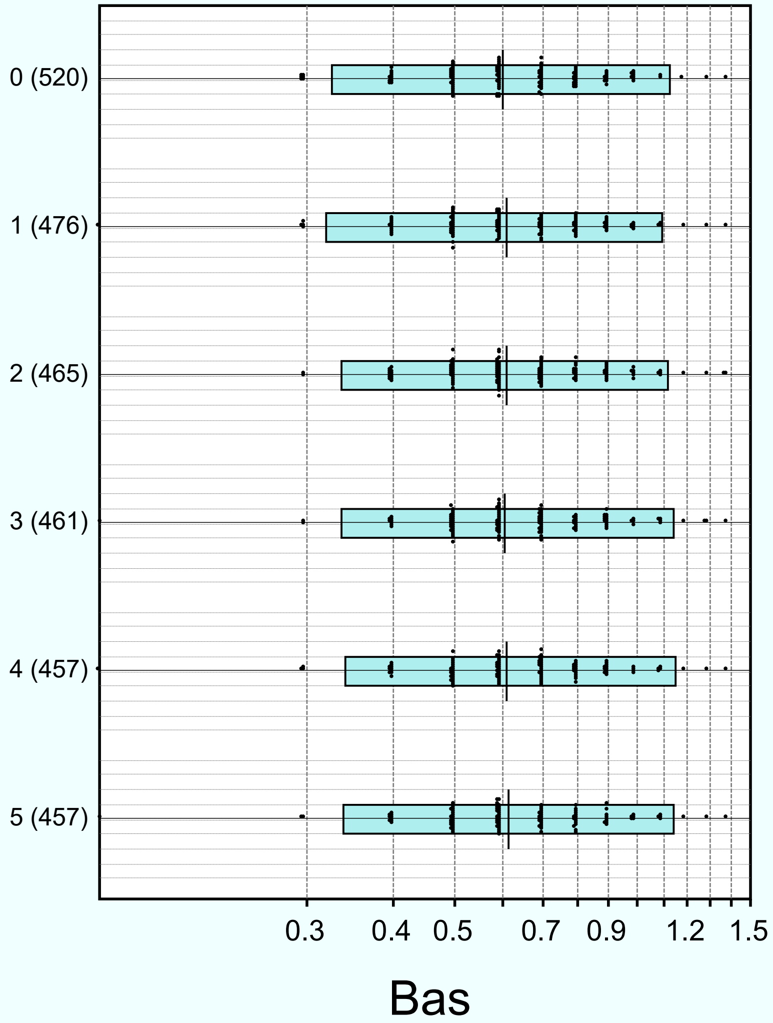

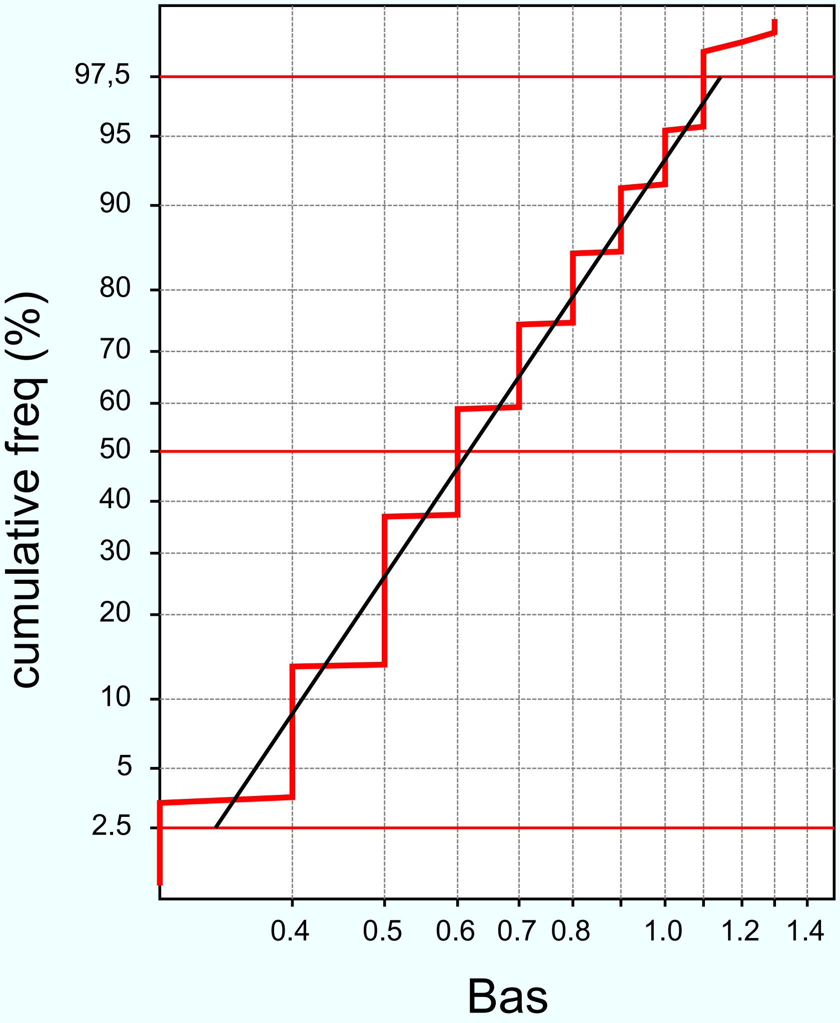

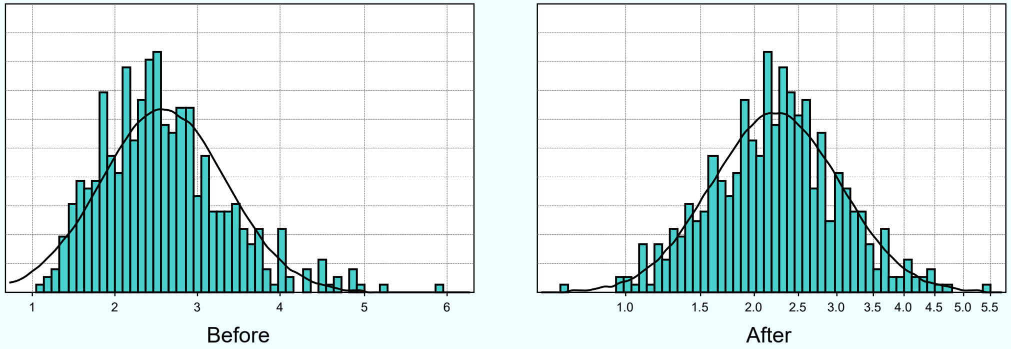

NeuAbs MF n=461
Para: 1.045 ~ 2.190 ~ 4.085
Nonpara: 1.042 ~ 2.220 ~ 4.357
Pow=0.518 TPos=0.677
Kurt=2.74 Skew=-0.061

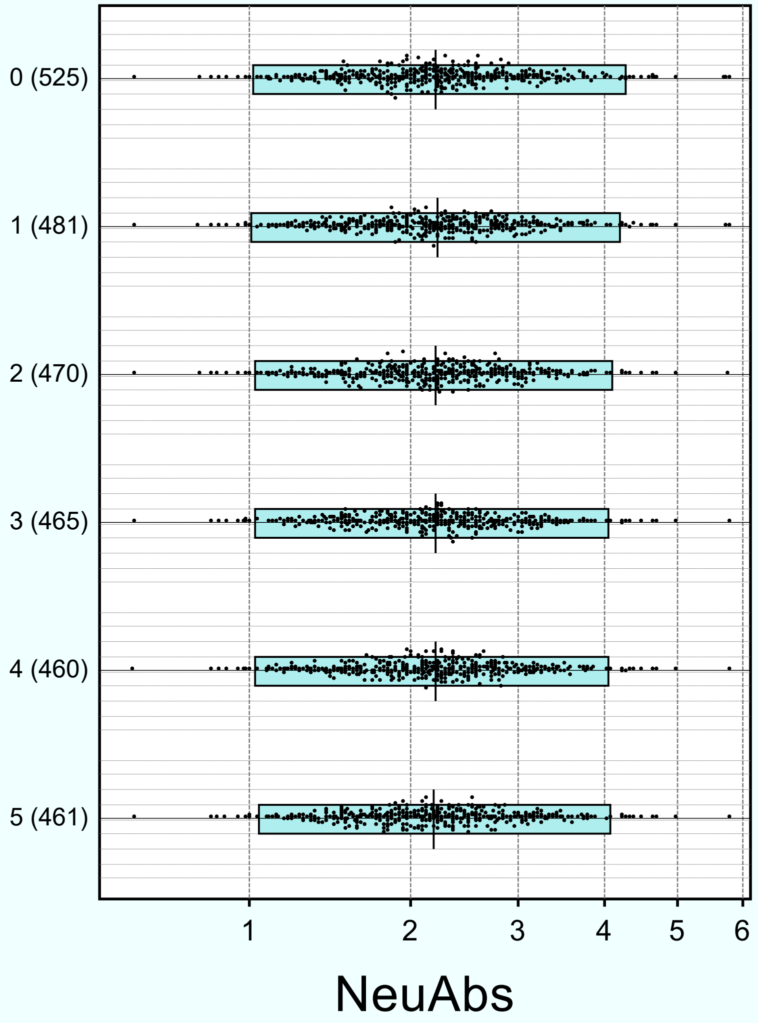

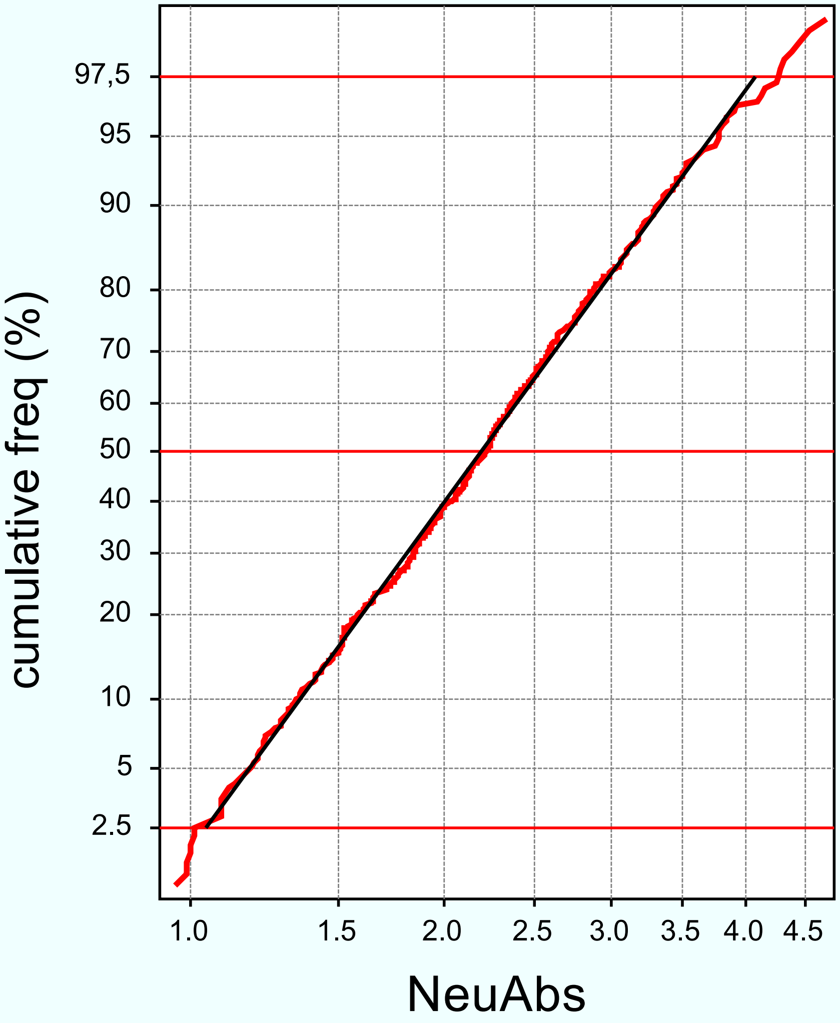

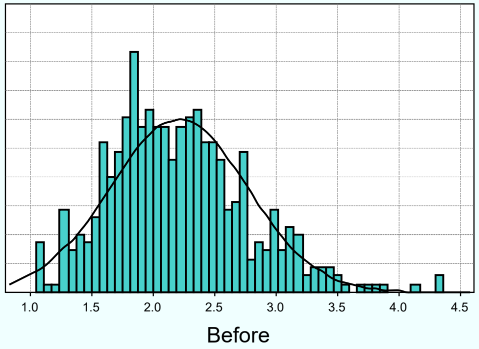

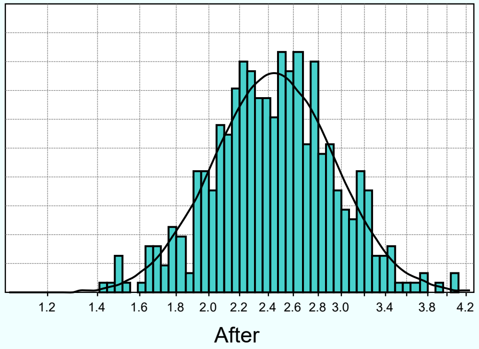

LymAbs MF n=461
Para: 1.292 ~ 2.127 ~ 3.402
Nonpara: 1.265 ~ 2.120 ~ 3.400
Pow=0.516 TPos=0.88
Kurt=2.716 Skew=-0.013

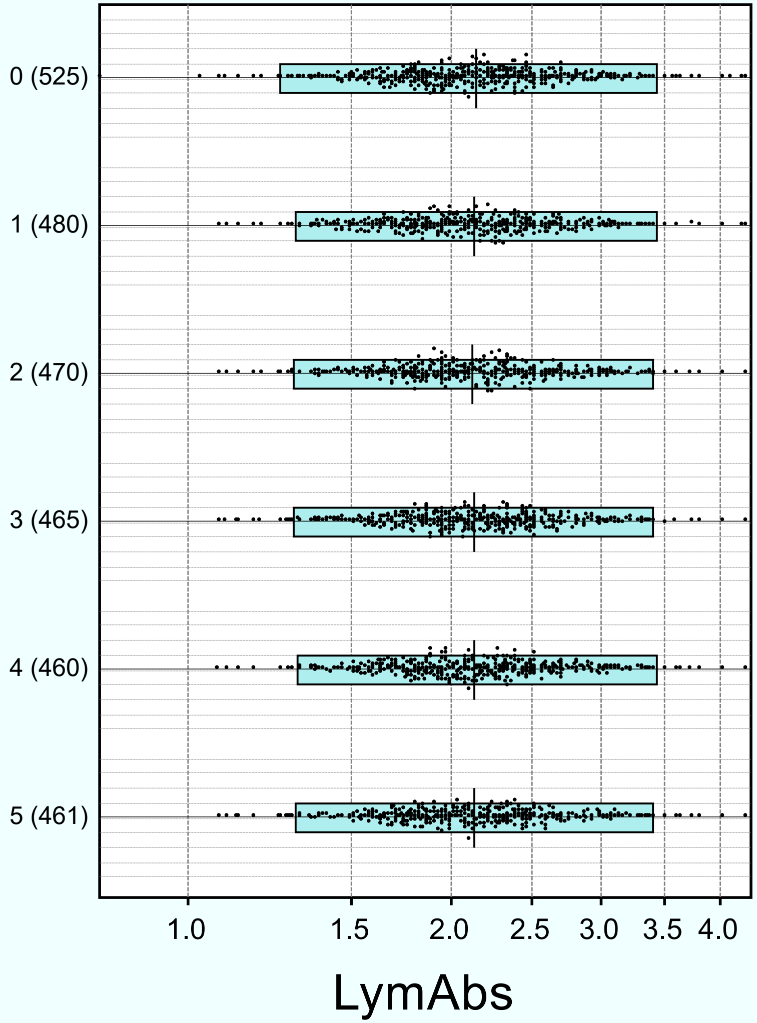

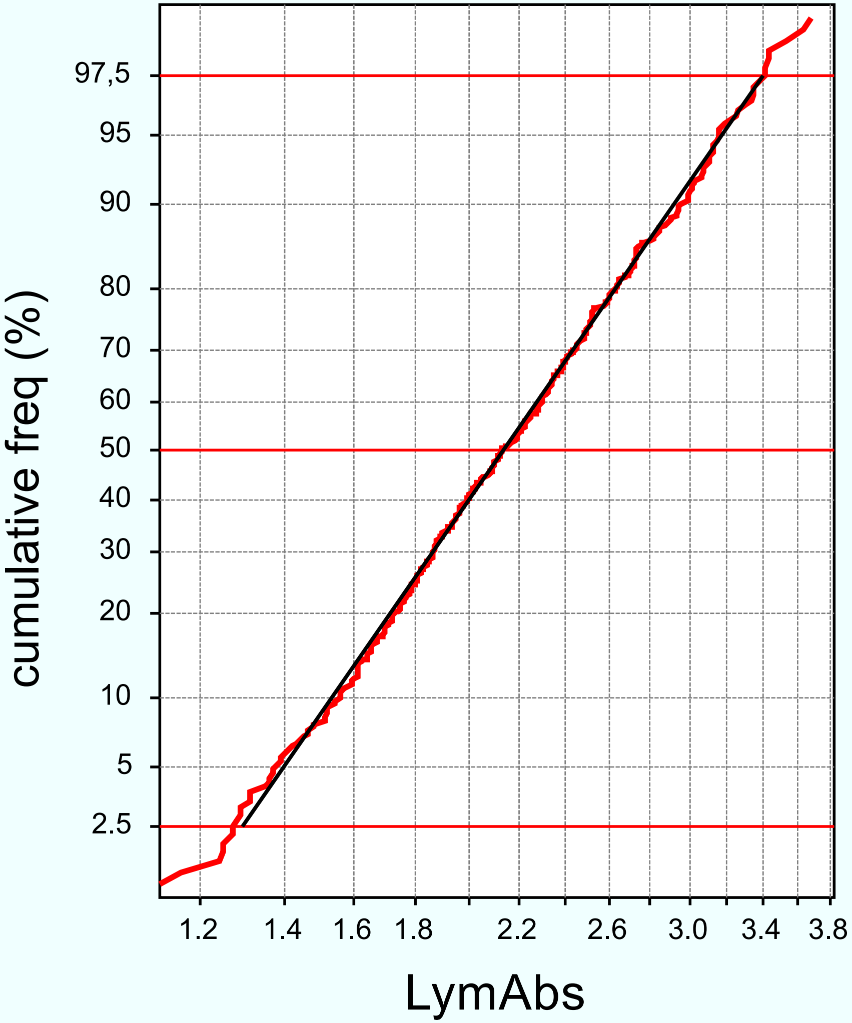

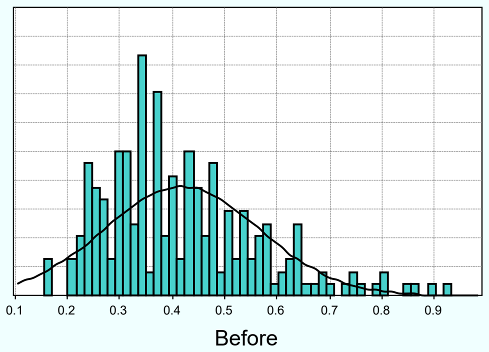

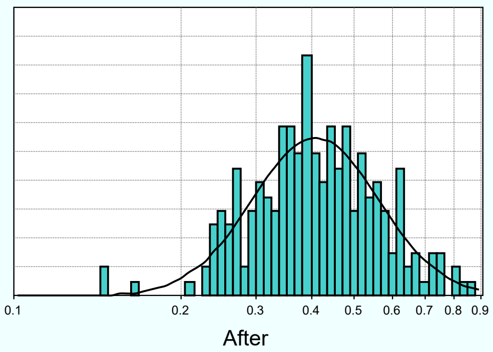

MonAbs M n=227
Para: 0.150 ~ 0.343 ~ 0.763
Nonpara: 0.159 ~ 0.340 ~ 0.765
Pow=0.335 TPos=0.088
Kurt=2.53 Skew=0.15

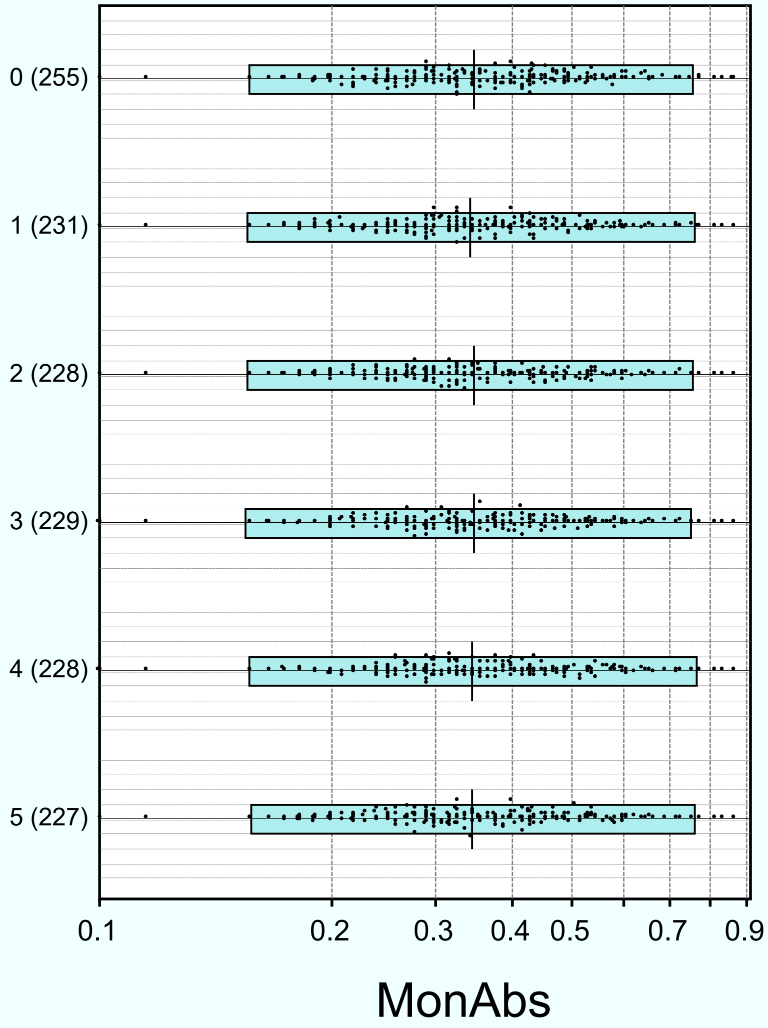

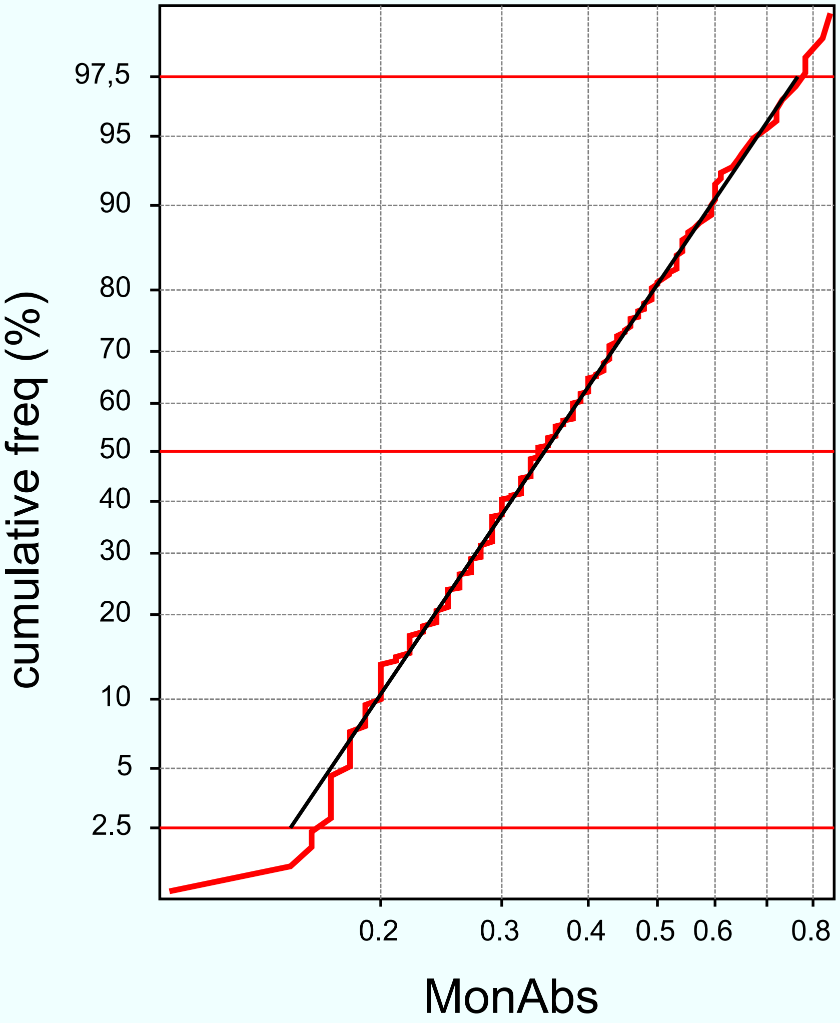

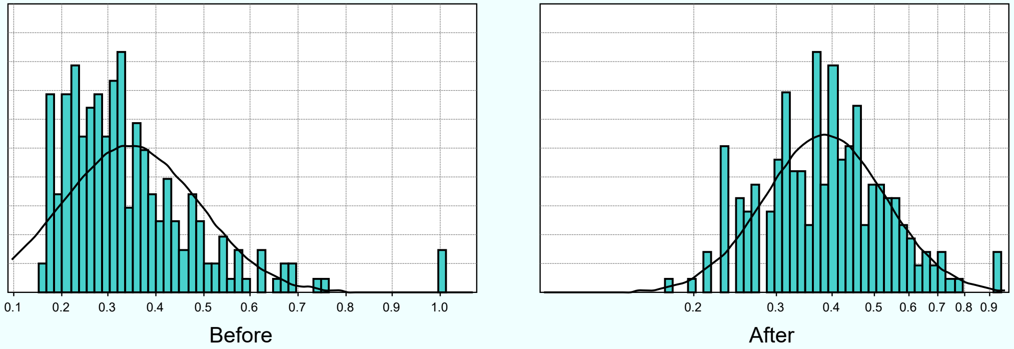

MonAbs F n=228
Para: 0.136 ~ 0.278 ~ 0.681
Nonpara: 0.141 ~ 0.280 ~ 0.682
Pow=0.26 TPos=0.105
Kurt=2.63 Skew=-0.062

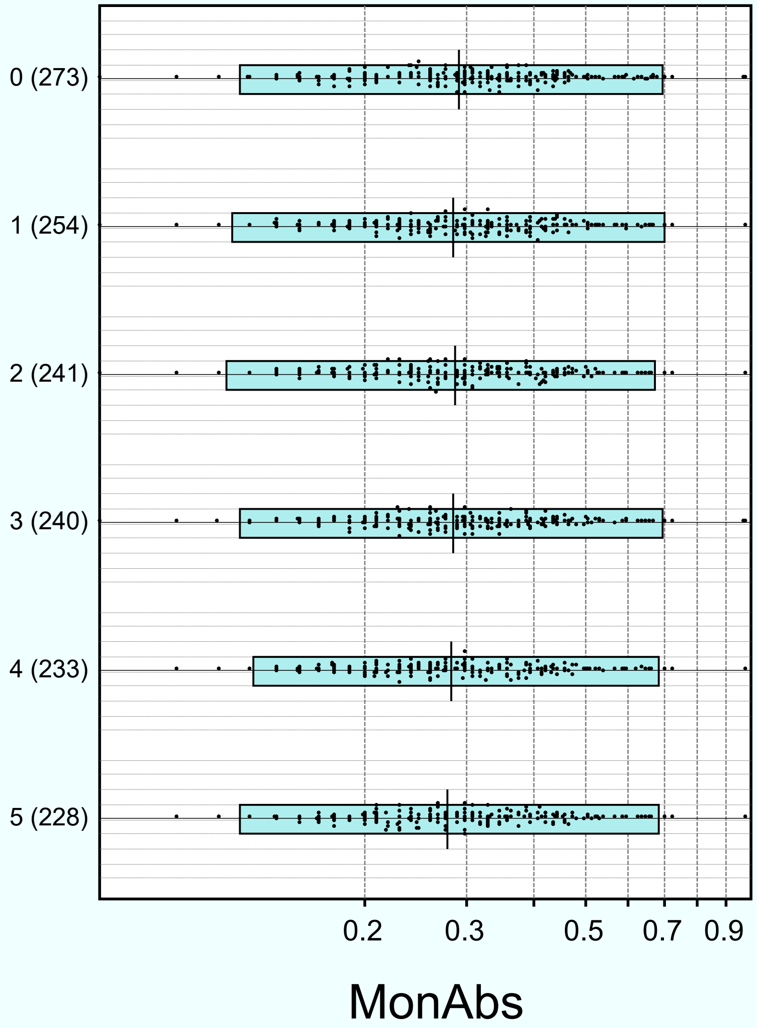

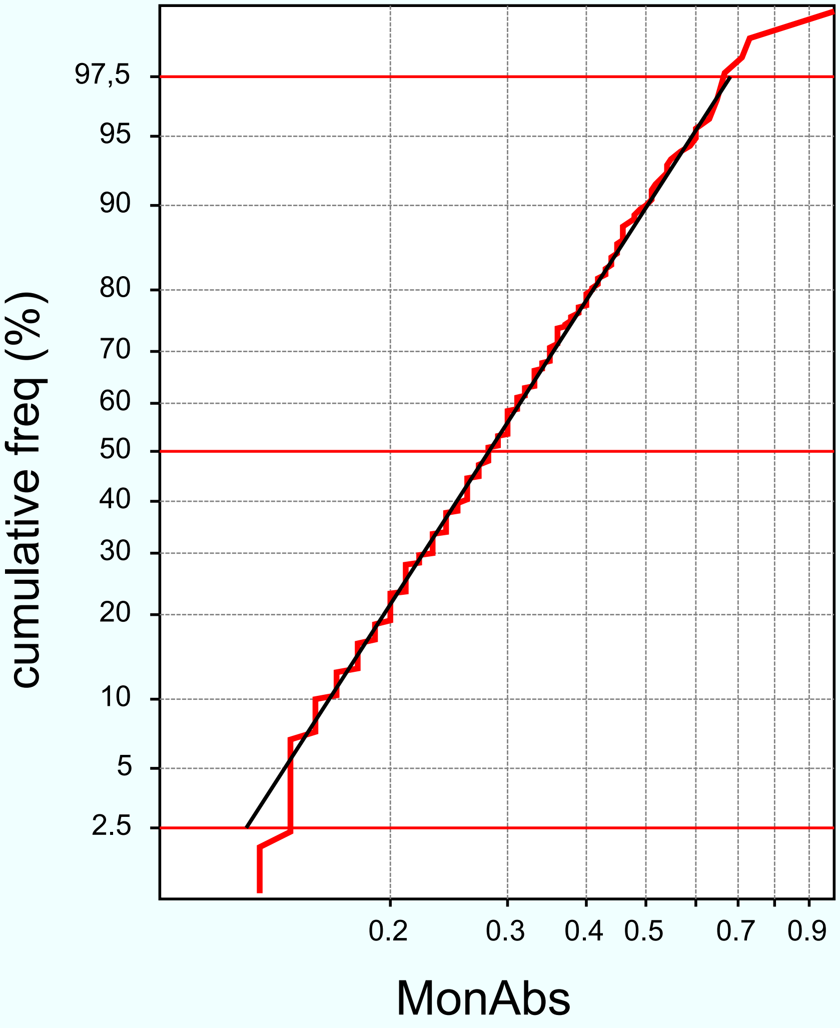

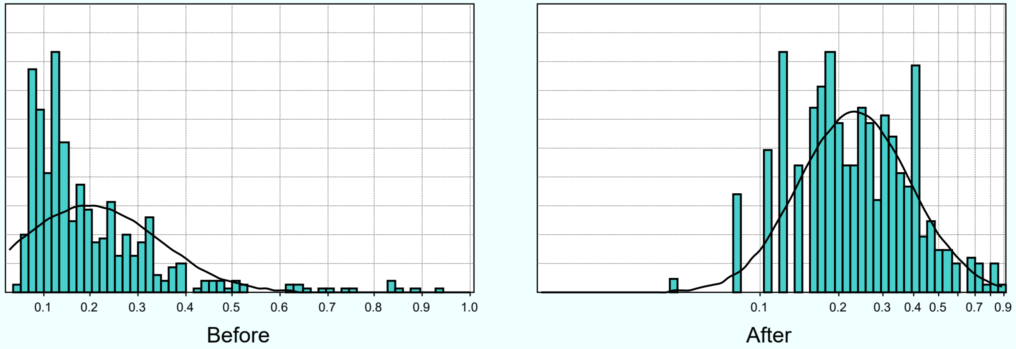

EosAbs MF n=460
Para: 0.042 ~ 0.133 ~ 0.595
Nonpara: 0.040 ~ 0.130 ~ 0.609
Pow=0.095 TPos=0.026
Kurt=2.599 Skew=0.081

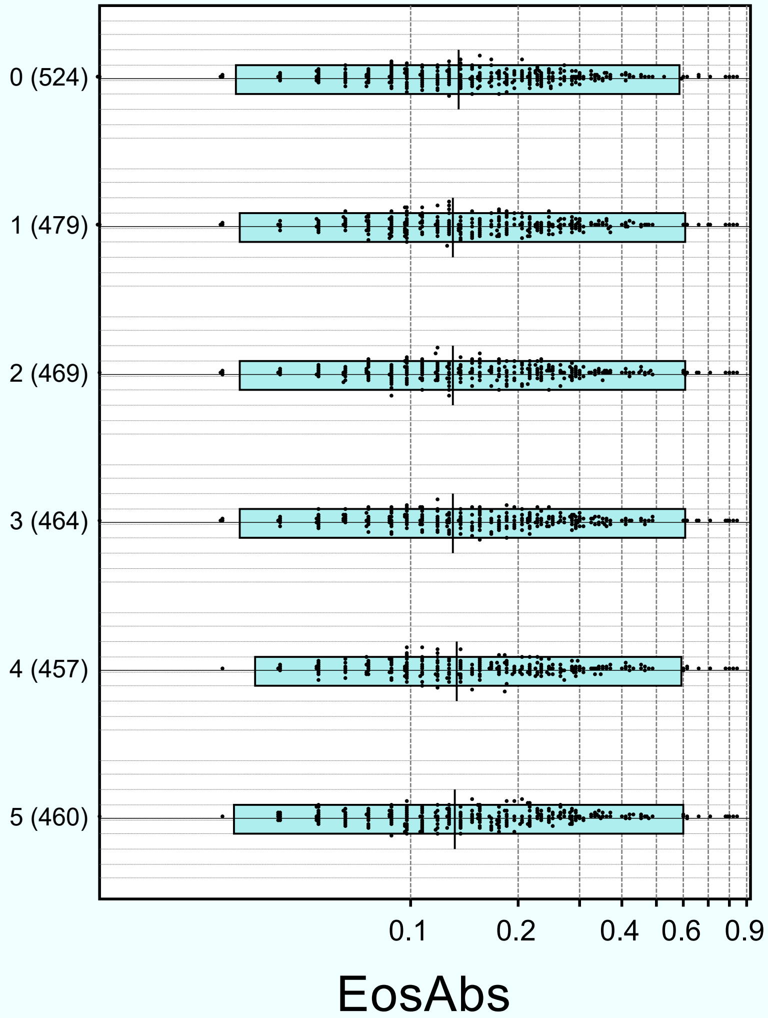

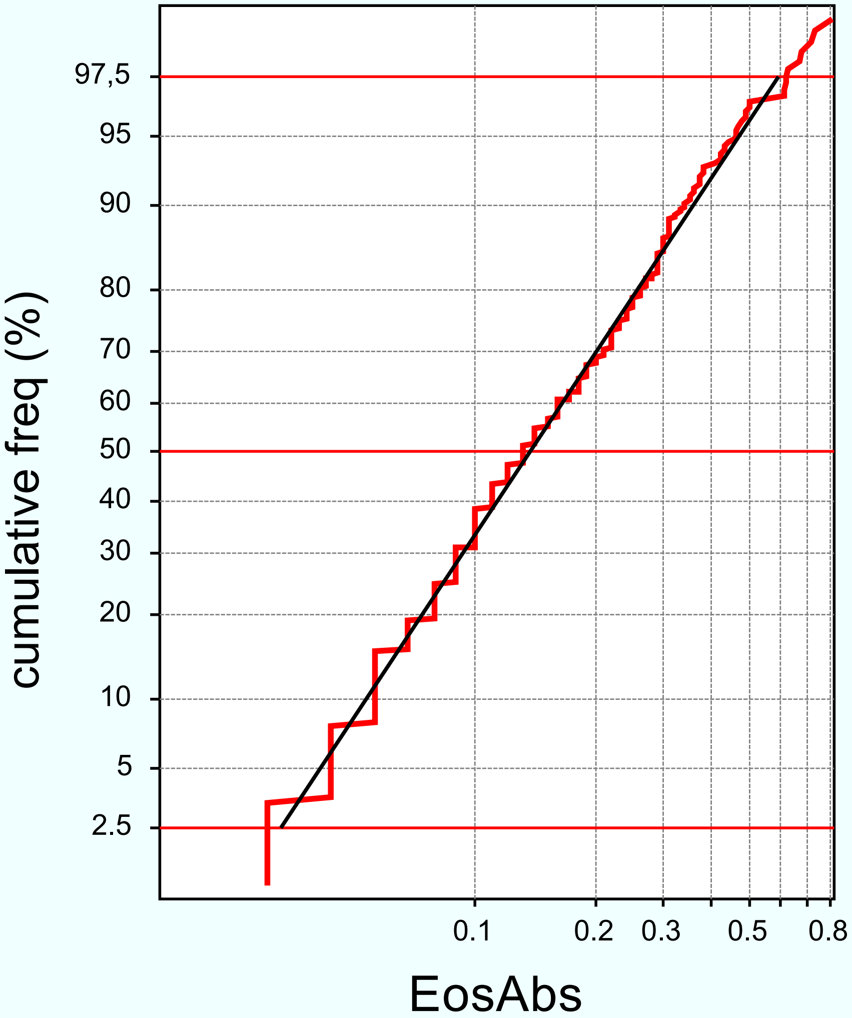

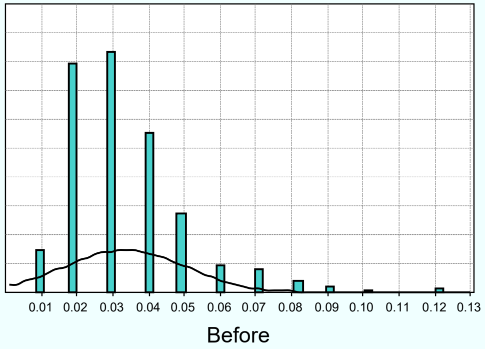

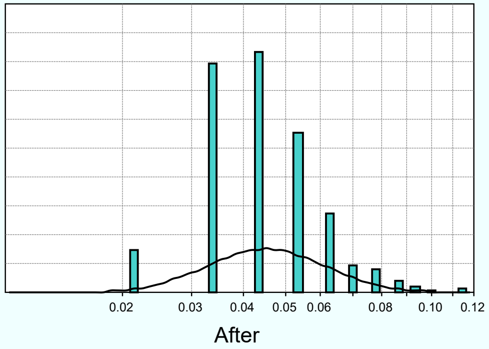

BasAbs MF n=461
Para: 0.012 ~ 0.030 ~ 0.073
Nonpara: 0.010 ~ 0.030 ~ 0.076
Pow=0.32 TPos=0.006
Kurt=3.453 Skew=-0.299

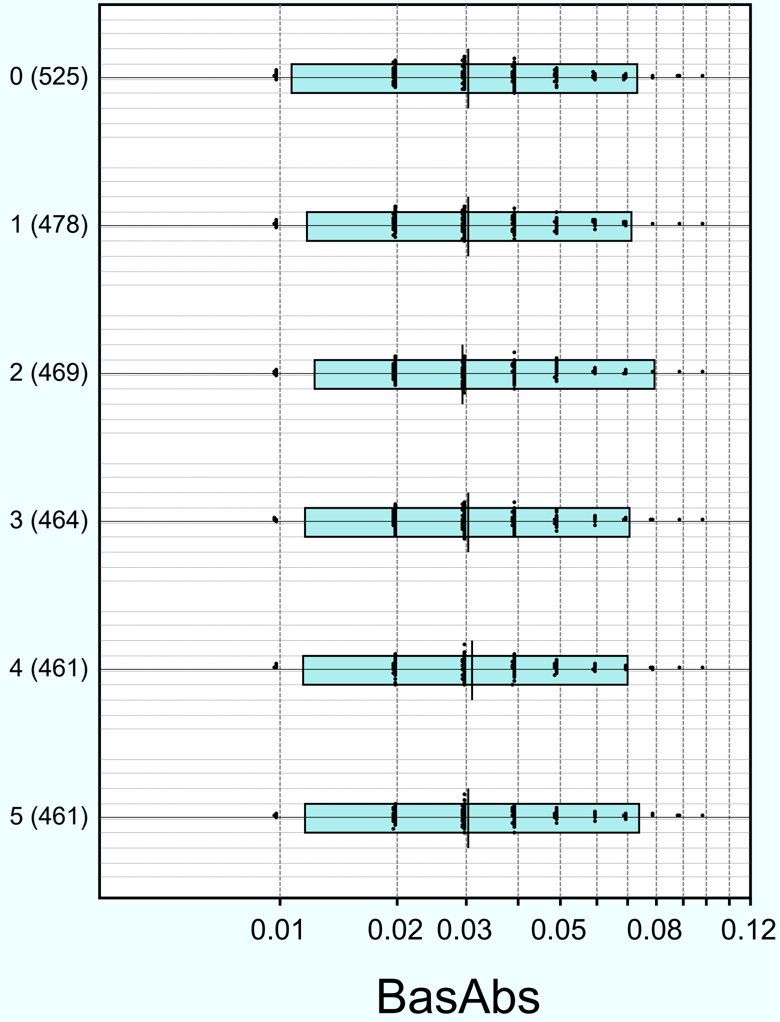

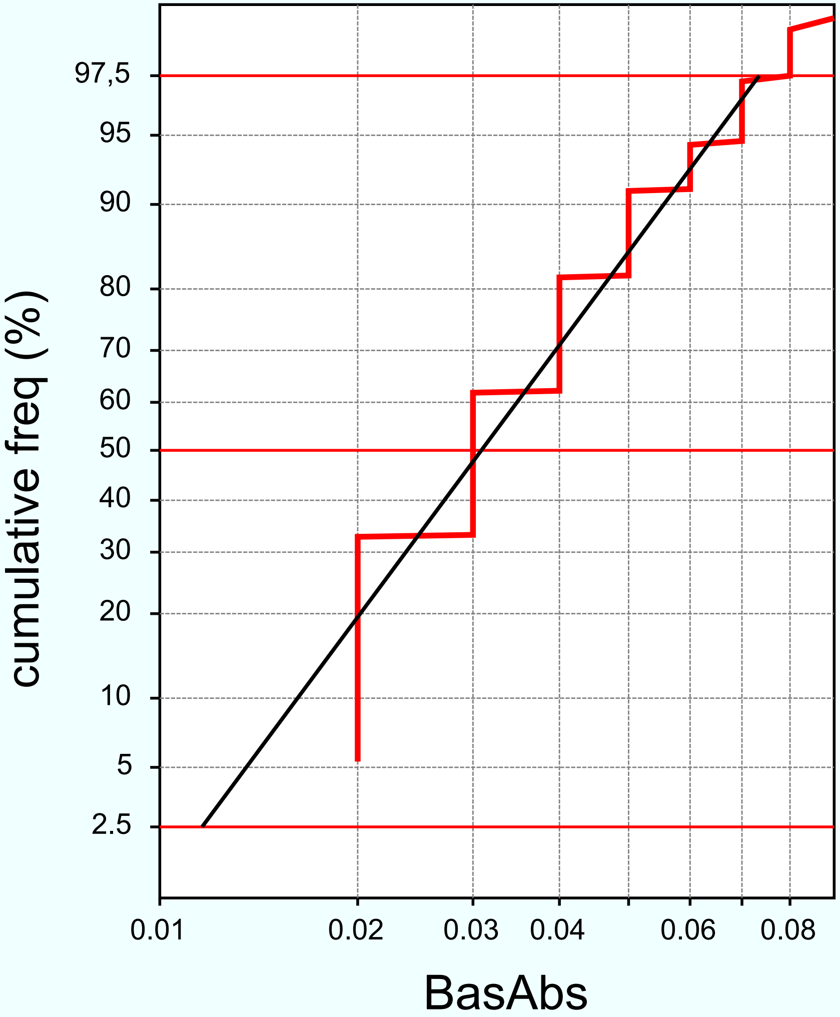

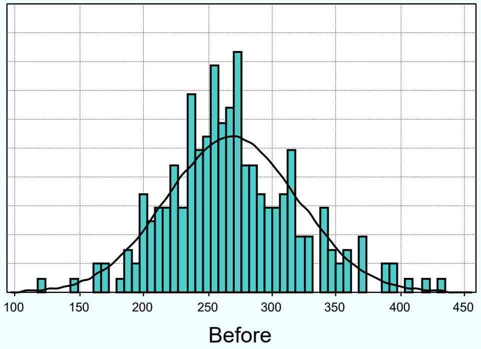

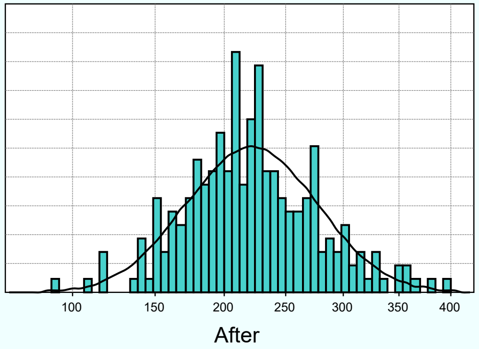

PLT M n=231
Para: 132.5 ~ 232.6 ~ 355.9
Nonpara: 126.3 ~ 230.0 ~ 368.4
Pow=0.744 TPos=74.54
Kurt=2.844 Skew=0.222

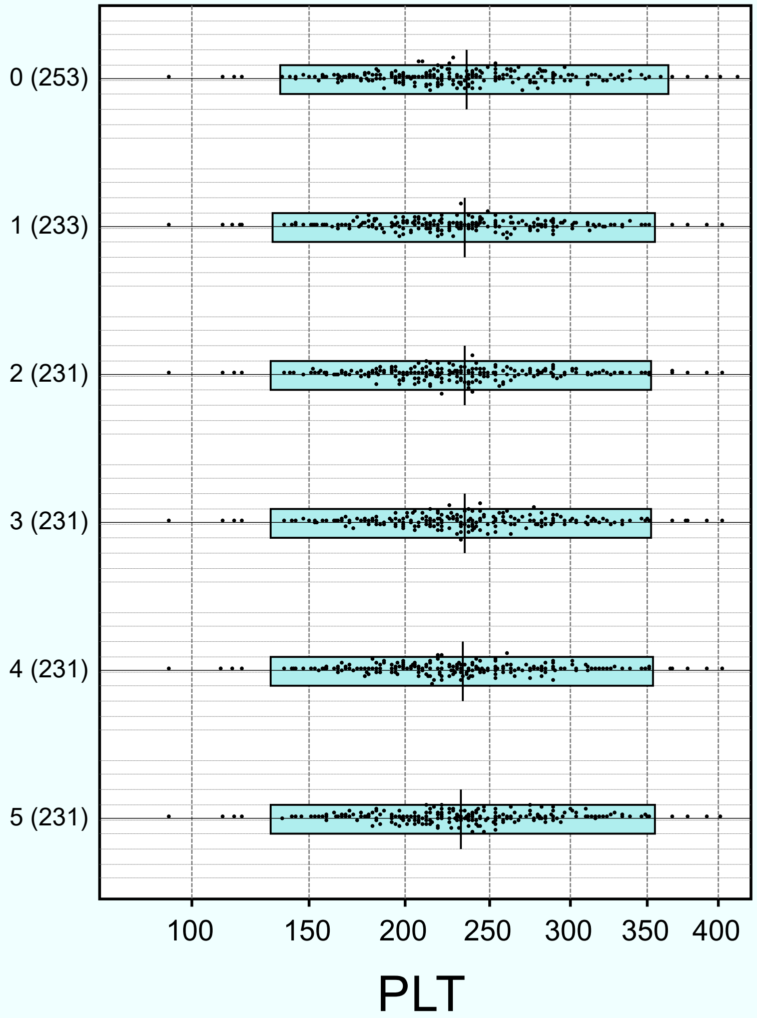

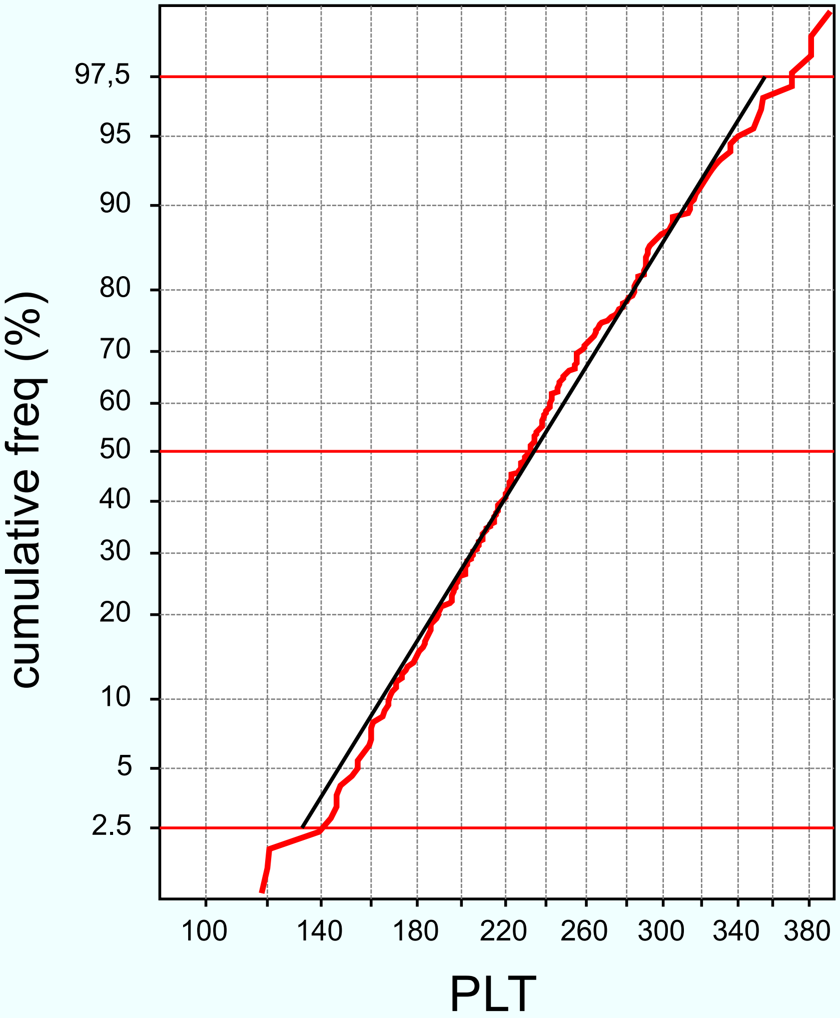

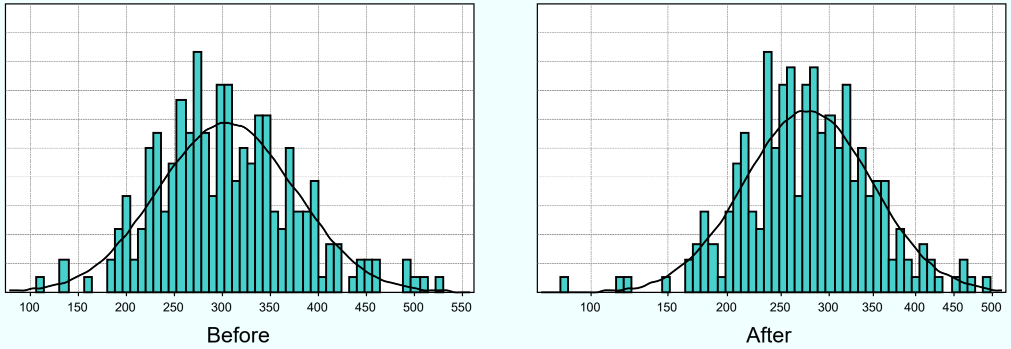

PLT F n=233
Para: 151.7 ~ 277.9 ~ 443.2
Nonpara: 150.4 ~ 276.0 ~ 459.3
Pow=0.653 TPos=68.48
Kurt=2.781 Skew=0.134

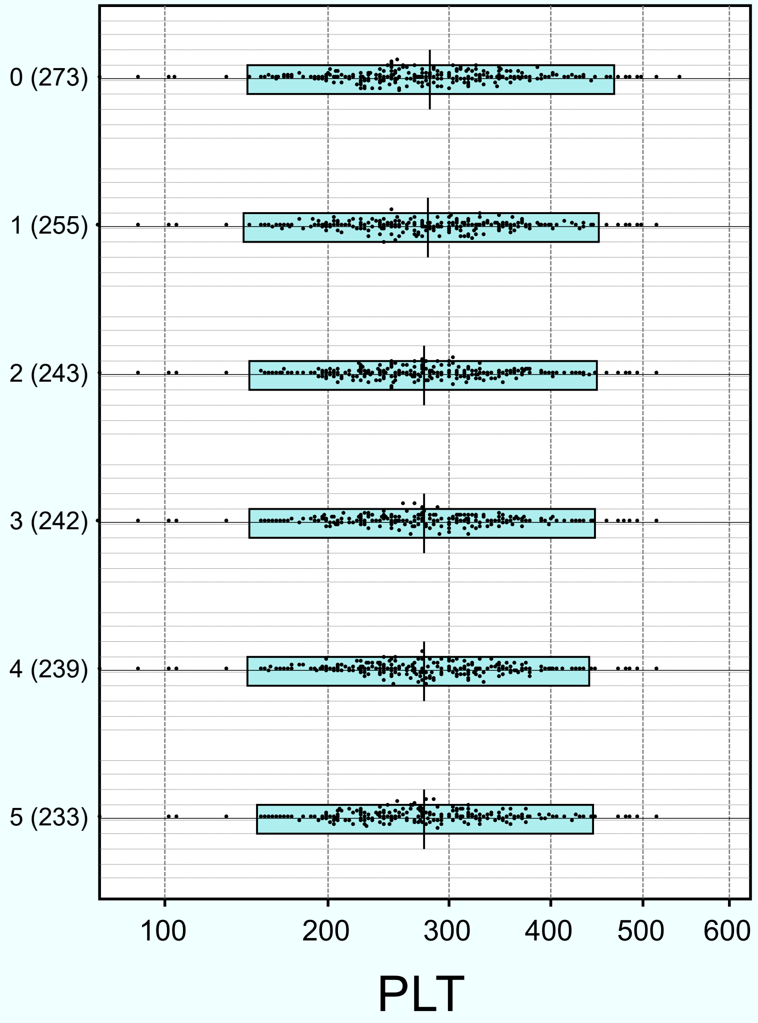

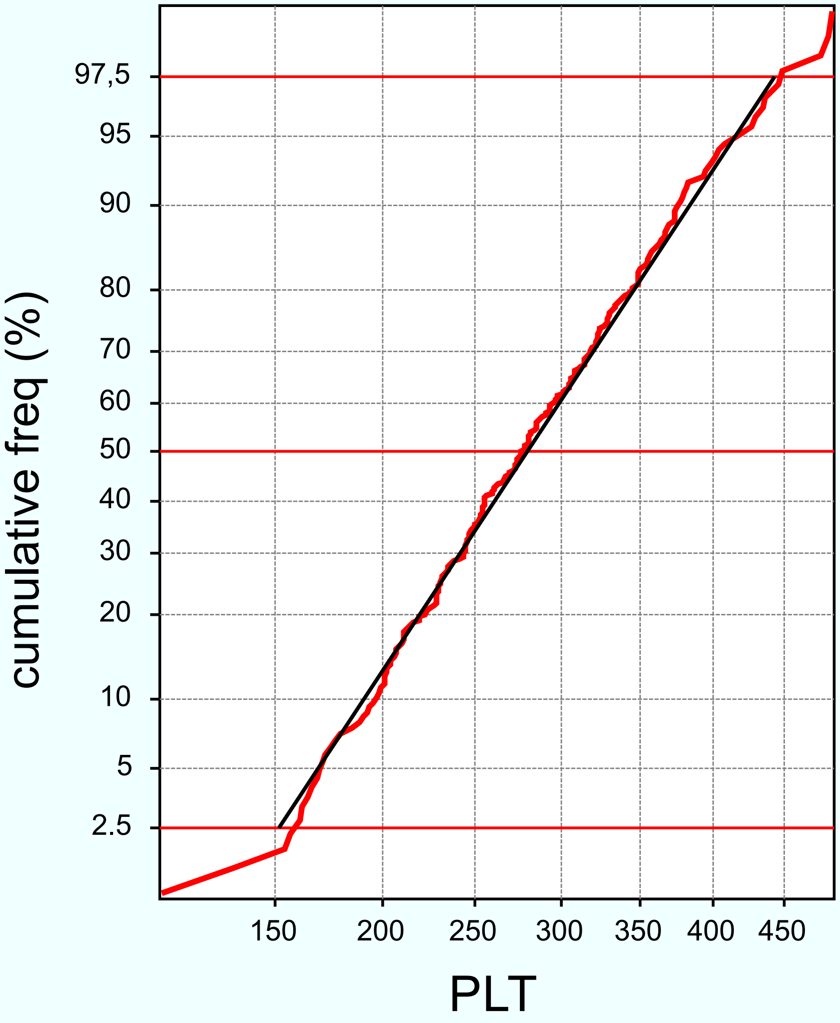

Supplement: S3 Fig — (PDF) [file pone.0198444.s003.pdf]
