# Supplementary material for: Complete blood count reference intervals from a healthy adult urban population in Kenya
Source: PLoS One. 2018 Jun 7;13(6):e0198444. doi: 10.1371/journal.pone.0198444 (PMC5991659; doi:10.1371/journal.pone.0198444)
Supplement: S1 Table — (PDF) [file pone.0198444.s004.pdf]

S1 Table: Standard deviation ratios for change in upper and lower limits of reference intervals by use of the LAVE method												
	**Parametric**						**Non-parametric**					
	**Male + Female**		**Male**		**Female**		**Male + Female**		**Male**		**Female**	
**Item**	**LL**	**UL**	**LL**	**UL**	**LL**	**UL**	**LL**	**UL**	**LL**	**UL**	**LL**	**UL**
RBC	0.25	0.09	**0.32**	0.00	**0.30**	**0.30**	0.22	0.06	0.28	0.02	**0.47**	0.23
Hb	**1.01**	0.32	0.28	0.00	**1.22**	0.00	**1.28**	0.00	0.37	0.00	**1.53**	0.09
Hct	**0.69**	0.00	0.00	0.00	**0.60**	**0.30**	**0.98**	0.00	**0.71**	0.00	**1.21**	0.00
MCV	**0.85**	0.00	0.19	0.02	**1.00**	0.04	**1.04**	0.00	0.18	0.04	**1.10**	0.03
MCH	**0.78**	0.00	**0.31**	0.05	**1.13**	0.00	**1.09**	0.00	0.28	0.00	**1.11**	0.04
MCHC	**0.52**	0.00	0.13	0.00	**0.65**	0.00	**0.63**	0.00	0.00	0.13	**0.91**	0.13
RDW	0.20	**1.21**	0.12	0.23	0.09	**1.87**	0.00	**1.92**	0.10	**0.83**	0.00	**2.25**
WBC	0.06	0.09	0.02	0.09	0.00	0.22	0.06	0.18	0.02	0.07	0.02	0.25
Neu %	0.02	0.09	0.05	0.01	0.02	0.05	0.01	0.03	0.02	0.01	0.10	0.03
Lym %	0.12	0.01	0.05	0.09	0.06	0.07	0.07	0.02	0.08	0.07	0.02	0.08
Mon %	0.04	0.08	0.04	0.07	0.00	0.25	0.04	0.04	0.07	0.11	0.05	0.27
Eos %	0.04	0.15	0.00	0.04	0.00	0.23	0.00	0.19	0.00	0.20	0.00	0.00
Bas %	0.00	0.00	0.00	0.00	0.00	0.00	0.00	0.00	0.00	0.00	0.00	0.00
Neu Abs	0.04	0.27	0.05	0.05	0.01	**0.33**	0.05	0.31	0.04	0.18	0.01	**0.47**
Lym Abs	0.09	0.07	0.04	0.12	0.14	0.10	0.07	0.13	0.02	0.29	0.07	0.04
Mon Abs	0.00	0.07	0.00	0.00	0.00	0.07	0.00	0.07	0.00	0.06	0.00	0.07
Eos Abs	0.00	0.07	0.07	0.13	0.00	0.09	0.00	0.00	0.00	0.21	0.00	0.16
Bas Abs	0.00	0.00	0.00	**0.56**	0.00	**0.78**	0.00	**0.56**	0.00	0.00	0.00	**0.78**
PLT	0.05	**0.33**	0.07	0.17	0.08	**0.33**	0.01	**0.51**	0.06	0.08	0.11	0.26

Key

%: percentage, LL: lower limit, Me: median, UL: upper limit, RBC: red blood cell count, Hb: haemoglobin, MCV: mean corpuscular volume, MCH: mean corpuscular haemoglobin, MCHC: mean corpuscular haemoglobin concentration, RDW: red cell distribution width, WBC: white blood cell count, Neu: neutrophil, Lym: lymphocyte, Mon: monocyte, Eos: eosinophil, Bas: basophil, Abs: absolute count, Plt: platelet count, SDRs > 0.3 are in bold
