# Supplementary material for: Complete blood count reference intervals from a healthy adult urban population in Kenya
Source: PLoS One. 2018 Jun 7;13(6):e0198444. doi: 10.1371/journal.pone.0198444 (PMC5991659; doi:10.1371/journal.pone.0198444)
Supplement: S2 Table — (PDF) [file pone.0198444.s005.pdf]

S2a Table: Parametric complete blood count reference intervals before and after latent abnormal value exclusion													
		Parametric											
		Male + Female				Male				Female			
Item	LAVE	N	LL	Me	UL	N	LL	Me	UL	N	LL	Me	UL
RBC (x10^12/L)	(−)	528	4.28	5.25	6.43	254	4.81	5.61	6.52	272	4.20	4.93	5.87
	(+)	463	4.41	5.29	6.48	226	4.94	5.62	6.52	229	4.31	4.93	5.76
Hb (g/dL)	(−)	527	11.2	15.4	18.5	254	14.2	16.6	18.7	272	10.6	14.2	16.5
	(+)	470	12.8	15.4	19.0	232	14.5	16.6	18.7	236	12.0	14.3	16.5
Hct (L/L)	(−)	528	0.35	0.45	0.55	253	0.43	0.49	0.55	272	0.34	0.42	0.50
	(+)	471	0.38	0.46	0.55	232	0.43	0.49	0.55	237	0.36	0.43	0.49
MCV (fl)	(−)	527	71.4	86.9	95.6	254	75.6	87.7	95.4	273	67.7	86.0	95.6
	(+)	466	75.7	87.4	95.6	228	76.5	87.9	95.5	232	73.4	86.7	95.8
MCH (pg)	(−)	526	23.2	29.4	32.8	255	24.5	29.8	32.9	272	22.0	28.9	32.7
	(+)	462	24.8	29.6	32.8	227	25.1	29.8	32.8	230	24.4	29.2	32.7
MCHC (g/dL)	(−)	524	31.8	33.7	35.2	254	32.3	33.9	35.4	271	31.5	33.5	35.0
	(+)	461	32.2	33.8	35.2	227	32.4	33.9	35.4	230	32.0	33.6	35.0
RDW (%)	(−)	517	11.5	13.0	16.4	251	11.2	13.0	14.9	270	11.5	13.1	17.9
	(+)	460	11.3	13.0	15.2	225	11.3	13.0	14.7	228	11.4	13.0	15.8
WBC (x10^9/L)	(−)	525	3.01	4.96	7.94	255	3.15	4.86	7.99	270	2.89	4.99	7.99
	(+)	464	3.08	4.92	7.83	229	3.13	4.83	8.10	232	2.89	4.95	7.72
Neu (%)	(−)	528	27.8	45.1	64.1	255	27.0	42.6	60.4	273	29.3	47.7	65.9
	(+)	463	28.0	44.7	63.3	227	27.4	42.6	60.3	230	29.5	47.4	65.4
Lym (%)	(−)	528	26.2	44.0	59.9	255	28.6	45.2	61.0	273	25.0	42.6	58.7
	(+)	463	27.2	44.2	60.0	227	28.2	45.1	60.3	230	25.5	42.9	59.3
Mon (%)	(−)	527	3.3	6.3	13.1	254	3.6	7.0	14.1	272	3.2	5.8	11.5
	(+)	461	3.4	6.3	13.3	226	3.5	7.0	14.3	229	3.2	5.7	11.0
Eos (%)	(−)	520	1.0	2.7	11.5	253	1.2	2.9	11.9	270	0.8	2.5	9.9
	(+)	455	1.1	2.7	11.9	225	1.2	3.0	11.8	227	0.8	2.4	9.4
Bas (%)	(−)	520	0.30	0.60	1.10	251	0.40	0.70	1.20	270	0.30	0.60	1.00
	(+)	456	0.30	0.60	1.10	224	0.40	0.70	1.20	228	0.30	0.60	1.00
Neu Abs (x10^9/L)	(−)	524	1.02	2.21	4.29	255	0.98	2.09	3.96	271	1.08	2.34	4.70
	(+)	460	1.05	2.19	4.08	227	1.02	2.06	3.92	229	1.07	2.31	4.42
Lym Abs (x10^9/L)	(−)	526	1.24	2.14	3.44	254	1.34	2.15	3.65	272	1.15	2.11	3.29
	(+)	461	1.29	2.13	3.40	226	1.36	2.14	3.58	230	1.22	2.10	3.24
Mon Abs (x10^9/L)	(−)	527	0.14	0.32	0.73	255	0.15	0.35	0.76	272	0.14	0.29	0.69
	(+)	462	0.14	0.31	0.74	227	0.15	0.34	0.76	229	0.14	0.28	0.68
Eos Abs (x10^9/L)	(−)	523	0.04	0.14	0.58	254	0.04	0.15	0.62	270	0.04	0.13	0.48
	(+)	460	0.04	0.13	0.59	226	0.05	0.15	0.64	228	0.04	0.12	0.49
Bas Abs (x10^9/L)	(−)	525	0.010	0.030	0.070	253	0.010	0.030	0.070	272	0.010	0.030	0.070
	(+)	461	0.010	0.030	0.070	226	0.010	0.030	0.080	224	0.010	0.030	0.060
PLT (x10^9/L)	(−)	525	141	259	431	254	137	235	365	272	146	284	468
	(+)	464	144	254	409	231	133	233	356	232	152	278	443

Key

%: percentage, LL: lower limit, Me: Median, UL: upper limit, RBC: Red blood cell count, Hb: Haemoglobin, MCV: Mean corpuscular volume, MCH: Mean corpuscular haemoglobin, MCHC: Mean corpuscular haemoglobin concentration, RDW: Red cell distribution width, WBC: White blood cell count, Neu: Neutrophil, Lym: Lymphocyte, Mon: Monocyte, Eos: Eosinophil, Bas: Basophil, Abs: absolute count, Plt: platelet count, LAVE: Latent abnormal values exclusion. (-): without LAVE, (+): with LAVE. Reference tests used in applying the LAVE method were Iron, Ferritin, Transferrin, Hb, Hct, MCV, Albumin, Globulins, CRP, WBC and PLT

S2b Table: Non-parametric complete blood count reference intervals before and after latent abnormal value exclusion

		Parametric											
		Male + Female				Male				Female			
Item	LAVE	N	LL	Me	UL	N	LL	Me	UL	N	LL	Me	UL
RBC (x10^12/L)	(−)	528	4.31	5.26	6.36	255	4.78	5.61	6.59	273	4.15	4.93	5.93
	(+)	463	4.42	5.31	6.39	227	4.90	5.61	6.60	230	4.33	4.94	5.84
Hb (g/dL)	(−)	528	10.8	15.4	18.5	255	14.0	16.6	18.6	273	10.2	14.2	16.5
	(+)	470	12.7	15.5	18.5	232	14.4	16.7	18.6	237	12.0	14.3	16.6
Hct (L/L)	(−)	528	0.34	0.45	0.54	255	0.41	0.49	0.54	273	0.32	0.42	0.49
	(+)	471	0.38	0.46	0.54	232	0.43	0.49	0.54	237	0.36	0.43	0.49
MCV (fl)	(−)	528	68.1	87.1	95.2	255	72.7	88.0	95.0	273	64.9	86.6	95.2
	(+)	466	73.8	87.6	95.2	228	73.7	88.2	95.2	232	71.6	87.1	95.4
MCH (pg)	(−)	528	22.0	29.5	32.6	255	23.5	30.0	32.6	273	20.7	29.1	32.6
	(+)	463	24.3	29.7	32.6	227	24.1	30.0	32.6	230	23.3	29.3	32.5
MCHC (g/dL)	(−)	528	31.6	33.7	35.2	255	32.3	34.0	35.3	273	31.2	33.6	35.0
	(+)	463	32.1	33.8	35.2	227	32.3	34.0	35.4	230	31.9	33.7	34.9
RDW (%)	(−)	528	11.2	13.0	17.9	255	11.2	13.0	15.9	273	11.3	13.0	18.7
	(+)	463	11.2	12.9	15.7	227	11.3	13.0	15.1	230	11.3	12.9	16.0
WBC (x10^9/L)	(−)	528	3.01	4.98	8.42	255	3.18	4.91	7.87	273	2.92	5.01	9.10
	(+)	467	3.09	4.94	8.18	230	3.15	4.87	7.96	234	2.95	4.97	8.73
Neu (%)	(−)	528	26.7	45.3	63.1	255	26.2	42.5	59.9	273	28.0	47.9	66.0
	(+)	463	26.8	44.9	62.8	227	26.4	42.5	60.0	230	28.9	47.8	65.7
Lym (%)	(−)	528	26.2	44.1	60.6	255	27.7	45.1	61.2	273	25.0	42.8	59.7
	(+)	463	26.8	44.2	60.8	227	27.0	45.1	60.6	230	25.2	43.0	60.4
Mon (%)	(−)	528	3.3	6.3	13.3	255	3.5	7.1	13.7	273	3.3	5.8	12.4
	(+)	463	3.4	6.3	13.2	227	3.3	7.1	14.0	230	3.2	5.7	11.8
Eos (%)	(−)	528	0.9	2.7	10.9	255	1.1	3.0	11.2	273	0.7	2.4	9.5
	(+)	463	0.9	2.7	11.4	227	1.1	3.0	10.7	230	0.7	2.3	9.5
Bas (%)	(−)	528	0.30	0.60	1.20	255	0.30	0.70	1.30	273	0.30	0.60	1.00
	(+)	463	0.30	0.60	1.20	227	0.30	0.70	1.30	230	0.30	0.60	1.00
Neu Abs (x10^9/L)	(−)	528	1.00	2.23	4.62	255	0.99	2.11	4.05	273	1.03	2.34	5.18
	(+)	463	1.04	2.22	4.36	227	1.02	2.08	3.92	230	1.04	2.32	4.74
Lym Abs (x10^9/L)	(−)	528	1.23	2.14	3.47	255	1.29	2.16	3.73	273	1.18	2.12	3.34
	(+)	463	1.27	2.12	3.40	227	1.28	2.15	3.56	230	1.22	2.11	3.32
Mon Abs (x10^9/L)	(−)	528	0.15	0.32	0.73	255	0.16	0.34	0.78	273	0.14	0.30	0.69
	(+)	463	0.15	0.31	0.74	227	0.16	0.34	0.77	230	0.14	0.28	0.68
Eos Abs (x10^9/L)	(−)	528	0.04	0.13	0.61	255	0.04	0.15	0.64	273	0.04	0.12	0.52
	(+)	463	0.04	0.13	0.61	227	0.04	0.14	0.61	230	0.04	0.12	0.54
Bas Abs (x10^9/L)	(−)	528	0.010	0.030	0.070	255	0.010	0.030	0.080	273	0.010	0.030	0.070
	(+)	463	0.010	0.030	0.080	227	0.010	0.030	0.080	230	0.010	0.030	0.060
PLT (x10^9/L)	(−)	528	141	253	450	255	130	232	373	273	142	280	480
	(+)	467	142	248	414	232	126	230	368	233	150	276	459

Key

%: percentage, LL: lower limit, Me: Median, UL: upper limit, RBC: Red blood cell count, Hb: Haemoglobin, MCV: Mean corpuscular volume, MCH: Mean corpuscular haemoglobin, MCHC: Mean corpuscular haemoglobin concentration, RDW: Red cell distribution width, WBC: White blood cell count, Neu: Neutrophil, Lym: Lymphocyte, Mon: Monocyte, Eos: Eosinophil, Bas: Basophil, Abs: absolute count, Plt: platelet count, LAVE: Latent abnormal values exclusion. Reference tests used in applying the LAVE method were Iron, Ferritin, Transferrin, Hb, Hct, MCV, Albumin, Globulins, CRP, WBC and PLT
