# Supplementary material for: Complete blood count reference intervals from a healthy adult urban population in Kenya
Source: PLoS One. 2018 Jun 7;13(6):e0198444. doi: 10.1371/journal.pone.0198444 (PMC5991659; doi:10.1371/journal.pone.0198444)
Supplement: S3 Table — (PDF) [file pone.0198444.s006.pdf]

S3 Table: Summary of selected reference interval studies for erythrocyte related parameters in sub-Saharan Africa

Study	Inclusion criteria	Statistical method	Sex	RBC ($x10^{12}$/L)			Hb (g/dL)			Hct (L/L)			MCV (fl)			MCH (pg)			MCHC (g/dL)			RDW (%)		
				n	LL	UL	n	LL	UL	n	LL	UL	n	LL	UL	n	LL	UL	n	LL	UL	n	LL	UL
[1]Kenya, Jan-Oct 2015, (This study)	Healthy volunteers, 18-65 yo	Parametric + LAVE method	M+F	463	4.41	6.48	470	12.8	19.0	471	0.38	0.55	466	75.7	95.6	462	24.8	32.8	461	32.2	35.2	460	11.3	15.2
			M	226	4.94	6.52	232	14.5	18.7	232	0.43	0.55	228	76.5	95.5	227	25.1	32.8	227	32.4	35.4	225	11.3	14.7
			F	229	4.31	5.76	236	12.0	16.5	237	0.36	0.49	232	73.4	95.8	230	24.4	32.7	230	32.0	35.0	228	11.4	15.8
[2]Kenya, Jul-Dec 2004, Kibaya *et al*	Healthy participants, 18-55 yo	Parametric or non-parametric (mid 95%)	M+F	1541	4.00	6.20	1541	6.7	11.1	1541	0.30	0.50	1541	68.8	97.2	1541	22.4	33.5	1541	32.2	35.3			
			M	1020	4.40	6.30	1020	8.3	11.3	1020	0.40	0.50	1020	71.4	98.2	1020	23.3	33.8	1020	32.4	35.3			
			F	521	3.70	5.60	521	5.9	10.0	521	0.30	0.50	521	66.0	95.7	521	21.3	33.0	521	32.2	35.2			
[3]Kenya, Jan 2007-Jun 2010 (Results for 18-34 yo), Odhiambo *et al*	Healthy sexually active individuals, 6-34 yo	Non-parametric (mid 95%)	M+F																					
			M	389	4.60	6.60	389	12.6	17.2	389	0.38	0.52	389	67.4	93.6									
			F	322	4.00	5.80	322	9.0	14.9	322	0.29	0.44	322	59.3	93.2									
[4]Uganda, Jan-Sep 2002 (Results for > 24 yo), Lugada *et al*	Healthy individuals, 1 week-92 yo	Non-parametric (mid 90%)	M+F																					
			M	410	3.80	6.00	410	11.1	16.8	410	0.32	0.48	410	69.9	95.2									
			F	435	3.70	5.30	435	10.1	14.3	435	0.30	0.41	435	67.7	92.6									
[5]Kenya, Uganda, Zambia, Rwanda, Dec 2004-Oct 2006, Karita *et al*	Healthy volunteers, 18–60 yo	Non-parametric (mid 95%)	M+F										2105	68.0	98.0									
			M	1083	4.00	6.40	1083	12.2	17.7	799	0.35	0.51												
			F	846	3.80	5.60	1022	9.5	15.8	846	0.29	0.45												
[6]Ethiopia, Published in 1998, Tsegaye *et al*	Healthy factory workers, 15-45 yo	Non-parametric (mid 95%)	M+F																					
			M	280	4.30	5.90	280	13.9	18.3	280	0.42	0.55												
			F	205	3.70	5.20	205	12.2	16.6	205	0.35	0.49												
[7]Tanzania, Dec 2004-Jul 2005 Saathof *et al*	Healthy volunteers, 19-48 yo	Non-parametric (mid 95%)	M+F	272	4.01	6.12	276	11.7	17.2	276	0.37	0.53	274	77.6	98.1	272	23.6	33.1	275	30.6	34.9			
			M	145	4.41	6.27	148	13.7	17.7	149	0.40	0.54	149	76.4	98.8	147	23.1	33.2	148	30.6	35.1			
			F	127	3.84	5.59	128	11.1	15.7	127	0.36	0.47	125	77.7	97.9	125	24.2	33.1	127	30.4	34.8			
[8]Rwanda, Aug-Dec 2011, Gahutu *et al*	Blood donors, 18-40 yo	Non-parametric (mid 95%)	M+F										296	77.0	95.0	296	26.0	33.0	296	32.0	37.0			
			M	206	4.20	6.30	206	12.7	17.5	206	0.38	0.54												
			F	90	3.40	5.50	90	11.0	16.0	90	0.36	0.48												
[9]Ghana, published in 2012, Dosoo *et al*	Healthy individuals, 18-59 yo	Non-parametric (mid 95%)	M+F	623	3.39	5.83	624	9.8	16.0	625	0.29	0.49	625	72.0	97.0	623	22.6	33.5	620	30.5	36.2	625	11.5	16.7
			M	316	3.79	5.96	316	11.3	16.4	316	0.33	0.51	316	70.0	98.0	316	22.7	33.5	315	30.6	36.0	316	11.5	16.7
			F	307	3.09	5.30	308	8.8	14.4	309	0.26	0.45	309	73.0	96.0	307	22.3	33.6	305	30.4	36.5	309	11.4	16.8
[10]Togo, Apr-Sep 2008, Kueviakoe *et al*	Blood donors, 17-58 yo	Non-parametric (mid 95%)	M+F	1349	3.10	6.40	1349	10.0	18.4	1349	0.28	0.54	1349	80.0	99.0	1349	25.0	37.0	1349	29.0	41.0			
			M	1047	3.30	6.40	1047	10.0	18.4	1047	0.28	0.54	1047	80.0	99.0	1047	25.0	36.0	1047	29.0	39.0			
			F	302	3.10	6.00	302	10.3	17.1	302	0.28	0.47	302	80.0	95.0	302	25.0	37.0	302	30.0	41.0			
[11]South Africa (Gauteng), Published in 2009, Lawrie *et al*	Healthy volunteers , 18-60 yo	Non-parametric (mid 95%)	M+F																					
			M	88	4.19	5.85	88	13.4	17.5	88	0.39	0.51	88	83.1	101.6	88	27.8	34.8	88	33.0	35.0	88	12.1	16.3
			F	631	3.93	5.40	631	11.6	16.4	631	0.34	0.48	631	78.9	98.5	631	26.1	33.5	631	32.7	34.9	631	12.4	17.3

Key

LL: lower limit, UL: upper limit, RBC: red blood cell count, Hb: haemoglobin, MCV: mean corpuscular volume, MCH: mean corpuscular haemoglobin, MCHC: mean corpuscular haemoglobin concentration, RDW: red cell distribution width, LAVE: latent abnormal values exclusion

[1] **Exclusion criteria**: BMI > 35 kg/m^2, consumption of ethanol ≥ 70 g per day, smoking more than 20 tobacco cigarettes per day, chronic illness, recent recovery from acute illness, injury or surgery requiring hospitalization, known carrier state of HBV, HCV or HIV, pregnant or within 1 year after child birth. **CBC analyser**: Beckman Coulter ACT 5 DIFF CP analyser (Brea, California, US).

[2] **Exclusion criteria**: Febrile, pregnant, HIV seropositive, screen positive for syphilis and malaria. **CBC analyser**: ACT 5Diff CP instrument (Beckman Coulter, Fullerton, CA, USA) [7].

[3] **Exclusion criteri**a: HIV positive, pregnant. **CBC analyser**: Coulter ACT 5Diff CP analyser (Beckman Coulter, France) [18].

[4] **Exclusion criteria**: HIV positive, moribund, mentally ill, institutionalized persons, missing personal or laboratory data. **CBC analyser**: Act 5 Diff instrument (Beckman Coulter) [19].

[5] **Exclusion criteria**: Acutely ill, significant findings on physical examination or if laboratory tests revealed that they were pregnant, HIV antibody positive, had evidence of hepatitis B or C infection or suspected syphilis. **CBC analyser**: Beckman Coulter AcT 5 diff CP (Beckman Coulter, USA) [6].

[6] **Exclusion criteria**: HIV positive, presence of any illness as defined by the World Health Organization staging systems for HIV infection and disease. **CBC analyser**: Coulter counter T540 [20].

[7] **Exclusion criteria**: HIV positive, pregnant or on medication, body temperature ≥37.5^{0}C or if clinical assessment revealed other signs or symptoms of disease that could influence the laboratory parameters of interest. **CBC analyser**: Sysmex KX-21N analyser (Sysmex Corp., Kobe, Japan) [21].

[8] **Exclusion criteria**: Alcohol abuse, medication, smoking, pregnant, breastfeeding, on oral contraception, on menses. **CBC analyser**: Coulter AcT 5diff and Sysmex KX-21N (Sysmex Corporation, Kobe, Japan) [22].

[9] **Exclusion criteria**: Acute or chronic respiratory, cardiovascular, gastrointestinal, hepatic or genitourinary conditions, blood donation or transfusion within the past 3 months, hospitalisation within past 1 month, any findings that would compromise laboratory parameters, pregnant or lactating mothers. **CBC analyser**: Micros 60 analysers (Horiba-ABX, Montpellier, France) [23].

[10] **Exclusion criteria**: HIV, HBV, and HCV viral infection, malaria, abnormal haemoglobin electrophoresis screening, presence of hypochromia. **CBC analyser**: Sysmex SF-3000 (Sysmex, Kobe, Japan) [24].

[11] **Exclusion criteria**: HIV, current clinical symptoms, immunosuppressive or corticosteroid medication, chemotherapy, hospitalizations, surgery or blood transfusions in the six months prior to screening, splenomegaly, pregnant, Hb < 12 g/dL. Menstruating women returned in 2 weeks. **CBC analyser**: Beckman Coulter LH 750 (Beckman Coulter, Fullerton, CA, USA) [25]
