# Supplementary material for: Complete blood count reference intervals from a healthy adult urban population in Kenya
Source: PLoS One. 2018 Jun 7;13(6):e0198444. doi: 10.1371/journal.pone.0198444 (PMC5991659; doi:10.1371/journal.pone.0198444)
Supplement: S4 Table — (PDF) [file pone.0198444.s007.pdf]

**S4 Table: Summary of selected reference interval studies for platelets and white blood cell related parameters in sub-Saharan Africa**

| Study | Inclusion criteria | Statistical method | Sex | WBC (x10$^9$/L) | | | Neu Abs (x10$^9$/L) | | | Lym Abs (x10$^9$/L) | | | Mon Abs (x10$^9$/L) | | | Eos Abs (x10$^9$/L) | | | Bas Abs (x10$^9$/L) | | | PLT (x10$^9$/L) | | |
|---|---|---|---|---|---|---|---|---|---|---|---|---|---|---|---|---|---|---|---|---|---|---|---|---|
| | | | | n | LL | UL | n | LL | UL | n | LL | UL | n | LL | UL | n | LL | UL | n | LL | UL | n | LL | UL |
| [1]Kenya, Jan-Oct 2015, (This study) | Healthy volunteers, 18-65 yo | Parametric + LAVE method | M+F | 464 | 3.08 | 7.83 | 460 | 1.05 | 4.08 | 461 | 1.29 | 3.40 | 462 | 0.14 | 0.74 | 460 | 0.04 | 0.59 | 461 | 0.01 | 0.07 | 464 | 144 | 409 |
| | | | M | 229 | 3.13 | 8.10 | 227 | 1.02 | 3.92 | 226 | 1.36 | 3.58 | 227 | 0.15 | 0.76 | 226 | 0.05 | 0.64 | 226 | 0.01 | 0.08 | 231 | 133 | 356 |
| | | | F | 232 | 2.89 | 7.72 | 229 | 1.07 | 4.42 | 230 | 1.22 | 3.24 | 229 | 0.14 | 0.68 | 228 | 0.04 | 0.49 | 224 | 0.01 | 0.06 | 232 | 152 | 443 |
| [2]Kenya, Jul-Dec 2004, Kibaya *et al* | Healthy participants, 18-55 yo | Parametric or non-parametric (mid 95%) | M+F | 1541 | 2.80 | 8.20 | 1541 | 0.91 | 4.72 | 1541 | 1.14 | 3.45 | 1541 | 0.13 | 0.60 | 1541 | 0.03 | 1.14 | 1541 | 0.01 | 0.08 | 1541 | 120 | 411 |
| | | | M | 1020 | 2.70 | 7.50 | 1020 | 0.87 | 4.32 | 1020 | 1.12 | 3.16 | 1020 | 0.13 | 0.59 | 1020 | 0.03 | 1.08 | 1020 | 0.01 | 0.09 | 1020 | 115 | 366 |
| | | | F | 521 | 3.00 | 9.10 | 521 | 0.99 | 5.56 | 521 | 1.29 | 3.96 | 521 | 0.16 | 0.64 | 521 | 0.03 | 1.22 | 521 | 0.01 | 0.07 | 521 | 124 | 444 |
| [3]Kenya, Jan 2007-Jun 2010 (Results for 18-34 yo), Odhiambo *et al* | Healthy sexually active individuals, 6-34 yo | Non-parametric (mid 95%) | M+F | | | | | | | | | | | | | | | | | | | | | |
| | | | M | 389 | 3.30 | 9.60 | 389 | 1.30 | 5.20 | 389 | 1.20 | 3.40 | 389 | 0.20 | 0.70 | 389 | 0.04 | 1.60 | 389 | 0.01 | 0.14 | 389 | 126 | 356 |
| | | | F | 322 | 3.70 | 9.10 | 322 | 1.30 | 5.00 | 322 | 1.40 | 3.80 | 322 | 0.20 | 0.80 | 322 | 0.04 | 1.20 | 322 | 0.02 | 0.09 | 322 | 147 | 454 |
| [4]Uganda, Jan-Sep 2002 (Results for > 24 yo), Lugada *et al* | Healthy individuals, 1 week-92 yo | Non-parametric (mid 90%) | M+F | 845 | 3.40 | 8.70 | 845 | 0.84 | 3.37 | 845 | 1.40 | 4.20 | 845 | 0.17 | 0.59 | 845 | 0.13 | 2.11 | 845 | 0.01 | 0.07 | | | |
| | | | M | | | | | | | | | | | | | | | | | | | 410 | 80 | 288 |
| | | | F | | | | | | | | | | | | | | | | | | | 435 | 100 | 297 |
| [5]Kenya, Uganda, Zambia, Rwanda, Dec 2004-Oct 2006, Karita *et al* | Healthy volunteers, 18–60 yo | Non-parametric (mid 95%) | M+F | 2105 | 3.10 | 9.10 | 2103 | 1.00 | 5.30 | 2105 | 1.20 | 3.70 | 2103 | 0.20 | 0.78 | 2104 | 0.04 | 1.53 | 1750 | 0.01 | 0.15 | 2105 | 126 | 438 |
| | | | M | | | | | | | | | | | | | | | | | | | | | |
| | | | F | | | | | | | | | | | | | | | | | | | | | |
| [6]Ethiopia, Published in 1998, Tsegaye *et al* | Healthy factory workers, 15-45 yo | Non-parametric (mid 95%) | M+F | 485 | 3.00 | 10.20 | | | | | | | | | | | | | | | | 485 | 98 | 337 |
| | | | M | | | | | | | | | | | | | | | | | | | 280 | 97 | 324 |
| | | | F | | | | | | | | | | | | | | | | | | | 205 | 98 | 352 |
| [7]Tanzania, Dec 2004-Jul 2005 Saathof *et al* | Healthy volunteers, 19-48 yo | Non-parametric (mid 95%) | M+F | 279 | 3.00 | 7.90 | 276 | 1.10 | 4.70 | 278 | 1.10 | 3.00 | | | | | | | | | | 274 | 150 | 395 |
| | | | M | 150 | 2.80 | 7.90 | 150 | 1.10 | 4.80 | 149 | 1.10 | 2.80 | | | | | | | | | | 147 | 147 | 356 |
| | | | F | 129 | 3.20 | 8.00 | 126 | 1.20 | 5.40 | 129 | 1.10 | 3.10 | | | | | | | | | | 127 | 151 | 425 |
| [8]Rwanda, Aug-Dec 2011, Gahutu *et al* | Blood donors, 18-40 yo | Non-parametric (mid 95%) | M+F | 296 | 2.90 | 7.11 | 296 | 1.00 | 4.10 | 296 | 1.10 | 3.20 | 296 | 0.20 | 0.60 | 296 | 0.05 | 0.74 | 296 | 0.01 | 0.08 | | | |
| | | | M | | | | | | | | | | | | | | | | | | | 206 | 115 | 336 |
| | | | F | | | | | | | | | | | | | | | | | | | 90 | 134 | 408 |
| [9]Ghana, published in 2012, Dosoo *et al* | Healthy individuals, 18-59 yo | Non-parametric (mid 95%) | M+F | 620 | 3.40 | 9.20 | | | | 624 | 1.20 | 4.40 | 624 | 0.20 | 1.00 | | | | | | | 625 | 89 | 380 |
| | | | M | 311 | 3.50 | 9.20 | | | | 316 | 1.20 | 5.20 | 316 | 0.20 | 1.40 | | | | | | | 316 | 88 | 352 |
| | | | F | 309 | 3.40 | 9.30 | | | | 308 | 1.20 | 4.40 | 308 | 0.20 | 0.90 | | | | | | | 309 | 89 | 403 |
| [10]Togo, Apr-Sep 2008, Kueviakoe *et al* | Blood donors, 17-58 yo | Non-parametric (mid 95%) | M+F | 1349 | 1.90 | 10.10 | 1349 | 0.50 | 5.40 | 1349 | 1.10 | 4.30 | 1349 | 0.05 | 0.80 | 1349 | 0.00 | 0.50 | 1349 | | | 1349 | 120 | 443 |
| | | | M | 1047 | 1.90 | 10.10 | 1047 | 0.50 | 5.40 | 1047 | 1.10 | 4.30 | 1047 | 0.05 | 0.80 | 1047 | 0.00 | 0.50 | | | | 1047 | 120 | 443 |
| | | | F | 302 | 2.20 | 7.80 | 302 | 0.50 | 4.40 | 302 | 1.20 | 4.30 | 302 | 0.05 | 0.80 | 302 | 0.00 | 0.50 | | | | 302 | 150 | 436 |
| [11]South Africa (Gauteng), Published in 2009, Lawrie *et al* | Healthy volunteers , 18-60 yo | Non-parametric (mid 95%) | M+F | | | | | | | | | | | | | | | | | | | | | |
| | | | M | 88 | 3.92 | 10.40 | 88 | 1.60 | 6.98 | 88 | 1.40 | 4.20 | 88 | 0.30 | 0.80 | 88 | 0.00 | 0.95 | 88 | 0.00 | 0.10 | 88 | 171 | 388 |
| | | | F | 631 | 3.90 | 12.60 | 631 | 1.60 | 8.30 | 631 | 1.40 | 4.50 | 631 | 0.20 | 0.80 | 631 | 0.00 | 0.40 | 631 | 0.00 | 0.10 | 631 | 186 | 454 |

Key

LL: lower limit,UL: upper limit, WBC: white blood cell count, Neu: Neutrophil, Lym: lymphocyte, Mon: monocyte, Eos: eosinophil, Bas: basophil, abs-absolute count, Plt-platelet count, LAVE: latent abnormal values exclusion

[1]**Exclusion criteria**: BMI > 35 $kg/m^2$, consumption of ethanol ≥ 70 g per day, smoking more than 20 tobacco cigarettes per day, chronic illness, recent recovery from acute illness, injury or surgery requiring hospitalization, known carrier state of HBV, HCV or HIV, pregnant or within 1 year after child birth. **CBC analyser**: Beckman Coulter ACT 5 DIFF CP analyser (Brea, California, US).

[2]**Exclusion criteria**: Febrile, pregnant, HIV seropositive, screen positive for syphilis and malaria. **CBC analyser**: ACT 5Diff CP instrument (Beckman Coulter, Fullerton, CA, USA) [7].

[3]**Exclusion criteri**a: HIV positive, pregnant. **CBC analyser**: Coulter ACT 5Diff CP analyser (Beckman Coulter, France) [18].

[4] **Exclusion criteria**: HIV positive, moribund, mentally ill, institutionalized persons, missing personal or laboratory data. **CBC analyser**: Act 5 Diff instrument (Beckman Coulter) [19].

[5] **Exclusion criteria**: Acutely ill, significant findings on physical examination or if laboratory tests revealed that they were pregnant, HIV antibody positive, had evidence of hepatitis B or C infection or suspected syphilis. **CBC analyser**: Beckman Coulter AcT 5 diff CP (Beckman Coulter, USA) [6].

[6] **Exclusion criteria**: HIV positive, presence of any illness as defined by the World Health Organization staging systems for HIV infection and disease. **CBC analyser**: Coulter counter T540 [20].

[7] **Exclusion criteria**: HIV positive, pregnant or on medication, body temperature ≥37.5$^0$C or if clinical assessment revealed other signs or symptoms of disease that could influence the laboratory parameters of interest. **CBC analyser**: Sysmex KX-21N analyser (Sysmex Corp., Kobe, Japan) [21].

[8] **Exclusion criteria**: Alcohol abuse, medication, smoking, pregnant, breastfeeding, on oral contraception, on menses. **CBC analyser**: Coulter AcT 5diff and Sysmex KX-21N (Sysmex Corporation, Kobe, Japan) [22].

[9] **Exclusion criteria**: Acute or chronic respiratory, cardiovascular, gastrointestinal, hepatic or genitourinary conditions, blood donation or transfusion within the past 3 months, hospitalisation within past 1 month, any findings that would compromise laboratory parameters, pregnant or lactating mothers. **CBC analyser**: Micros 60 analysers (Horiba-ABX, Montpellier, France) [23].

[10] **Exclusion criteria**: HIV, HBV, and HCV viral infection, malaria, abnormal haemoglobin electrophoresis screening, presence of hypochromia. **CBC analyser**: Sysmex SF-3000 (Sysmex, Kobe, Japan) [24].

[11] **Exclusion criteria**: HIV, current clinical symptoms, immunosuppressive or corticosteroid medication, chemotherapy, hospitalizations, surgery or blood transfusions in the six months prior to screening, splenomegaly, pregnant, Hb < 12 g/dL. Menstruating women returned in 2 weeks. **CBC analyser**: Beckman Coulter LH 750 (Beckman Coulter, Fullerton, CA, USA) [25].
